# Analysis of the global burden of neonatal disorders and risk factors from 1990 to 2021: findings from the global burden of disease study 2021

**DOI:** 10.3389/fpubh.2025.1618334

**Published:** 2025-10-24

**Authors:** Huilin Wang, Jun Xiong, Fuyu Yang, Wenzhen Wang, Peng Yu, Runze Chen

**Affiliations:** ^1^School of Public Administration, Hangzhou Normal University, Hangzhou, Zhejiang, China; ^2^Engineering Research Center of Mobile Health Management System, Ministry of Education, Hangzhou, Zhejiang, China

**Keywords:** neonatal disorders, disease burden, GBD, prevalence, death, disability-adjusted life years

## Abstract

**Background:**

Neonatal health represents a critical global issue, encapsulating both the advancements and challenges faced by health systems at both global and national levels. Neonatal disorders not only impose a significant disease burden, diminishing the quality of life for affected individuals, but they also contribute to substantial economic strain.

**Methods:**

This article utilizes the Global Burden of Disease 2021 database to describe the global burden of neonatal disorders in 2021 through age-standardized incidence rate, age-standardized prevalence rate, age-standardized death rate, and age-standardized disability-adjusted life years rate. Additionally, it employs the estimated annual percentage change to delineate the trends in the global burden of neonatal disorders from 1990 to 2021.

**Results:**

The global age-standardized incidence rate, age-standardized prevalence rate, age-standardized death rate, and age-standardized disability-adjusted life years rate for neonatal disorders were 437.43 (95% UI = 433.20 to 441.95) per 100,000 population, 437.43 (433.20 to 441.95), 29.57 (25.37 to 34.26), and 2941.98 (2547.76 to 3384.20), respectively.

**Conclusion:**

From 1990 to 2021, the global burden of neonatal disorders exhibited a general downward trend, with a particularly pronounced burden in South Asia and Southern Sub-Saharan Africa. Countries characterized by low socio-demographic index and low-middle socio-demographic index faced a disproportionately higher burden of neonatal disorders. Low birth weight emerged as a significant contributor to both the age-standardized death rate and the age-standardized disability-adjusted life years rate associated with global neonatal disorders. Therefore, health departments across various nations should prioritize the enhancement of healthcare systems and the quality of care to alleviate the burden of neonatal disorders.

## 1 Introduction

Neonatal health is a global issue that reflects both the progress and challenges faced by health systems at both the global and national levels (1). Neonatal disorders mean disturbance of normal state of body, organs and abnormal function of a newborn (2). The incidence and prevalence of neonatal disorders (NDs) are influenced by various factors, including air pollution, short gestation, and low birth weight (3). The Global Burden of Disease database quantifies the burden of NDs. Over 25 and 75% of mortality of neonates take place before the completion of the first 24 h of birth and the 1st week of life, respectively, globally (4–7). More than two-thirds of all infants as well as about 50% of all children aged <5 years died as neonates (5, 8). According to the Global Burden of Disease (GBD) database in 2021, the global number of ND cases was approximately 152.55 million, resulting in about 1.83 million deaths and approximately 186.36 million disability-adjusted life years. NDs not only impose a disease burden and reduce the quality of life for patients, but they also create a substantial economic burden. Previous studies have shown that in Spain, the treatment costs associated with preterm birth and bronchopulmonary dysplasia are significant (9). Neonatal systemic infections can lead to both immediate and long-term health impacts (10). Therefore, it is crucial to study the burden of NDs both globally and regionally, and to mitigate the health impacts and economic burdens associated with these diseases.

Previous studies have demonstrated that the mortality rate of NDs in China decreased by 84.3% from 1990 to 2019, while the incidence rate declined by 30.3% (3). Premature birth remains the leading cause of neonatal death, accounting for 18% of total deaths among children under 5 years old (11, 12). Over the past three decades, the burden of PM2.5-related neonatal encephalopathy has intensified (13). Additionally, from 1990 to 2019, global cases of neonatal sepsis rose by 14.33%, with an annualized ASIR increasing by 14.34% (14). In 2021, Pakistan reported the highest hemolytic disease mortality rate in the South Asia region (15). However, comprehensive studies addressing the global and regional disease burden of NDs and their various etiologies in 2021 are scarce.

This study aims to leverage the GBD 2021 database to investigate the global burden of neonatal diseases across four world regions, 21 regions, 204 countries, and five distinct socio-demographic index (SDI) categories. The analysis encompasses neonatal preterm birth (NPB), neonatal encephalopathy due to birth asphyxia and trauma (NE), neonatal sepsis and other neonatal infections (NS), hemolytic disease and other neonatal jaundice (HD), as well as other neonatal disorders. Furthermore, the study seeks to estimate the annual percentage change in the burden of NDs from 1990 to 2021, explore gender disparities in the burden of NDs globally in 2021, and assess the global burden of NDs attributable to three risk factors: particulate matter pollution, short gestation, and low birth weight. The findings aim to provide valuable references for health departments in formulating policies to mitigate the global burden of NDs.

## 2 Methods

### 2.1 Data source

GBD represents the largest and most comprehensive global observational epidemiological study to date, assessing the burden of 371 diseases and the impact of 88 risk factors across 204 countries and 21 regions (16). Utilizing the GBD 2021 data tools,^1^ this article aims to compile data on the burden of NDs, NPB, NE, NS, HD, and other neonatal disorders. The analysis encompasses four world regions, 21 regions, 204 countries, and five different socio-demographic index (SDI) categories for the year 2021, along with the estimated annual percentage change in the burden of NDs from 1990 to 2021. The study specifically examines the global burden of NDs in 2021 attributed to three risk factors: particulate matter pollution, short gestation, and low birth weight, while also stratifying the burden by gender.

### 2.2 Statistical analysis

This study employed statistical indicators, including age-standardized incidence rate (ASIR), age-standardized prevalence rate (ASPR), age-standardized mortality rate (ASDR), age-standardized disability-adjusted life years (DALYs) rate, and a 95% uncertainty interval (UI), to describe the global, national, and regional burden of NDs in 2021. The estimated annual percentage change (EAPC) was utilized to delineate trends in the burden of NDs globally, nationally, and regionally from 1990 to 2021. A linear regression model was established, represented by the equation y = *α* + *β*x + *ε*, where y = ln(rate), x denotes the year, ε signifies the error term, and *β* indicates the trend direction of the age-standardized rate (ASR) (17). The EAPC was calculated using the formula EAPC = 100 × (℮β - 1) along with its 95% confidence interval (CI) (18). A 95% CI is a range with an upper and lower number that is calculated from a sample where the true value is unknown (19). This study applies the Concentration Index (CI) and the Slope Index of Inequality (SII) to assess the relative and absolute socioeconomic inequalities associated with the global burden of NDs, respectively. Additionally, the Das Gupta decomposition method is employed to analyze the effects of population growth, aging, and epidemiological changes on the global burden of NDs. According to the GBD 2021 database, this article quantifies the global and national/regional burden of NDs in 2021 attributable to three risk factors: particulate matter pollution, short gestation, and low birth weight, utilizing ASDR and age-standardized DALYs rates. The SDI is categorized into five levels: low SDI, low-middle SDI, middle SDI, high-middle SDI, and high SDI. The SDI employs data on fertility rates, education levels, and per capita income to quantify the developmental status of a country or region (20).

### 2.3 Inequality analysis

This paper utilizes the Concentration Index (CI) and the Slope Index of Inequality (SII) to assess relative and absolute socioeconomic inequalities in the burden of NDs, respectively. The Concentration Index (CI) is determined by the area under the Lorenz concentration curve and is defined as the cumulative fraction of DALYs against the cumulative relative distribution of the population ranked by SDI (21). The Slope Index of Inequality (SII) is calculated by regressing the age-standardized DALY rates of the national all-age population on the SDI-related relative position scale (22).

### 2.4 Decomposition analysis

The decomposition analysis evaluated the impacts of population growth, population aging, and epidemiological changes (23). This study utilizes the Das Gupta decomposition method to examine the effects of these demographic factors on the burden of NDs globally, across four world regions, 21 regions, and 204 countries.

### 2.5 Data visualization

This paper employs the JD_GBDR_V2.36_packed statistical analysis tool to generate statistical charts and tables that illustrate the global burden of NDs in 2021. It further analyzes the trends in the global burden of NDs from 1990 to 2021, examines the burden of NDs across five different countries categorized by SDI, and assesses the global and national/regional burden of NDs in 2021 attributable to three risk factors: particulate matter pollution, short gestation, and low birth weight.

## 3 Results

### 3.1 Global level

#### 3.1.1 Incidence and prevalence

The global ASIR of NDs decreased from 466.94 (95% UI = 461.65 to 473.62) per 100,000 population in 1990 to 437.43 (433.20 to 441.95) in 2021, with an EAPC of −0.36 (95% CI = −0.44 to −0.28) (Table 1). In 2021, among the global NPB, NE, NS, HD, the global ASIR of NPB was the highest, at 348.41 (345.58 to 351.26). From 1990 to 2021, the global HD ASIR EAPC was 0.02 (−0.05 to 0.08), while the global ASIRs for NPB, NE, and NS exhibited a downward trend, with the global NS EAPC being the lowest at −0.80 (−0.98 to −0.62) (Table 1). The global ASPR of NDs increased from 1691.50 (1516.96 to 1853.00) in 1990 to 437.43 (433.20 to 441.95) in 2021, with an EAPC of 0.60 (0.58 to 0.62) (Table 1). In 2021, among NPB, NE, NS, and HD, the global NPB ASPR was the highest at 1633.61 (1453.78 to 1818.24). From 1990 to 2021, the global ASPRs for NPB, NE, NS, and HD all demonstrated an upward trend, with the global NE ASPR EAPC being the highest at 2.16 (2.11 to 2.21), and the global NPB ASPR EAPC being the lowest at 0.39 (0.37 to 0.41; Table 1).

**Table 1:** Global burden of neonatal disorders and five neonatal disorder etiologies in 1990 and 2021, and the EAPC trends of global neonatal disorders and five neonatal disorder etiologies from 1990 to 2021.

Disease	Incidence			Prevalence			Deaths			DALYs		
	1990 ASR	2021 ASR	1990–2021 EAPC	1990 ASR	2021 ASR	1990–2021 EAPC	1990 ASR	2021 ASR	1990–2021 EAPC	1990 ASR	2021 ASR	1990–2021 EAPC
NDs	466.94 (461.65 to 473.62)	437.43 (433.20 to 441.95)	−0.36 (−0.44 to −0.28)	1691.50 (1516.96 to 1853.00)	2018.39 (1854.60 to 2185.48)	0.60 (0.58 to 0.62)	46.06 (43.66 to 48.81)	29.57 (25.37 to 34.26)	−1.49 (−1.57 to −1.40)	4343.25 (4121.18 to 4595.48)	2941.98 (2547.76 to 3384.20)	−1.31 (−1.38 to −1.24)
NPB	358.94 (356.24 to 361.84)	348.41 (345.58 to 351.26)	−0.27 (−0.34 to −0.20)	1455.98 (1265.59 to 1628.81)	1633.61 (1453.78 to 1818.24)	0.39 (0.37 to 0.41)	20.27 (18.65 to 21.90)	11.94 (10.05 to 14.16)	−1.67 (−1.72 to −1.62)	1962.26 (1814.42 to 2111.62)	1254.24 (1088.18 to 1465.54)	−1.40 (−1.44 to −1.36)
NE	20.22 (19.92 to 20.51)	17.16 (16.94 to 17.41)	−0.56 (−0.62 to −0.50)	129.73 (91.46 to 182.41)	242.03 (210.30 to 273.72)	2.16 (2.11 to 2.21)	13.81 (12.65 to 15.71)	9.75 (8.26 to 11.71)	−1.24 (−1.37 to −1.10)	1270.67 (1164.59 to 1443.71)	932.14 (796.29 to 1101.54)	−1.11 (−1.24 to −0.99)
NS	78.98 (75.39 to 84.88)	62.7 (61.18 to 64.48)	−0.80 (−0.98 to −0.62)	93.55 (62.62 to 138.38)	125.86 (97.03 to 156.32)	0.96 (0.89 to 1.03)	4.77 (4.23 to 5.31)	3.76 (3.18 to 4.40)	−0.93 (−1.06 to −0.80)	450.01 (396.86 to 503.96)	370.55 (315.48 to 425.82)	−0.79 (−0.91 to −0.67)
HD	8.80 (6.64 to 11.65)	9.16 (6.94 to 12.07)	0.02 (−0.05 to 0.08)	17.75 (16.07 to 19.74)	26.12 (23.96 to 28.77)	1.35 (1.32 to 1.38)	1.61 (1.40 to 1.99)	0.54 (0.42 to 0.69)	−3.44(−3.57 to −3.31)	152.39 (132.46 to 186.20)	58.33 (46.55 to 71.69)	−3.06 (−3.16 to −2.96)
Other neonatal disorders	_	_	_	_	_	_	5.6 (4.18 to 6.75)	3.58 (2.58 to 4.34)	−1.63 (−1.76 to −1.50)	507.92 (379.35 to 610.35)	326.72 (236.53 to 394.4)	−1.61 (−1.75 to −1.48)

#### 3.1.2 Deaths

The global ASDR for NDs decreased from 46.06 (43.66 to 48.81) in 1990 to 29.57 (25.37 to 34.26) in 2021, reflecting an EAPC of −1.49 (−1.57 to −1.40; Table 1). In 2021, the global ASDR for NPB was the highest among neonatal diseases, recorded at 11.94 (10.05 to 14.16), surpassing that of NE, NS, and other neonatal disorders. Between 1990 and 2021, the global ASDR for NPB, NE, NS, HD, and other neonatal disorders exhibited a declining trend. Notably, the global EAPC for NS ASDR was the highest at −0.93 (−1.06 to −0.80), whereas the global EAPC for HD ASDR was the lowest at −3.44 (−3.57 to −3.31; Table 1).

#### 3.1.3 DALYs

The global age-standardized rate of DALYs for NDs decreased from 4343.25 (4121.18 to 4595.48) in 1990 to 2941.98 (2547.76 to 3384.20) in 2021, yielding an EAPC of −1.31 (−1.38 to −1.24; Table 1). In 2021, the global age-standardized DALYs rate for NPB was the highest among neonatal diseases, recorded at 1254.24 (1088.18 to 1465.54), surpassing those for NE, NS, and other neonatal disorders. From 1990 to 2021, the global age-standardized DALYs rates for NPB, NE, NS, HD, and other neonatal disorders exhibited a declining trend. Notably, the EAPC for the age-standardized DALYs rate of NS was the highest globally at −0.79 (−0.91 to −0.67), while the global EAPC for the age-standardized DALYs rate of HD was the lowest at −3.06 (−3.16 to −2.96; Table 1).

### 3.2 African, American, Asian, European level

#### 3.2.1 Incidence and prevalence

**Figure 1:**
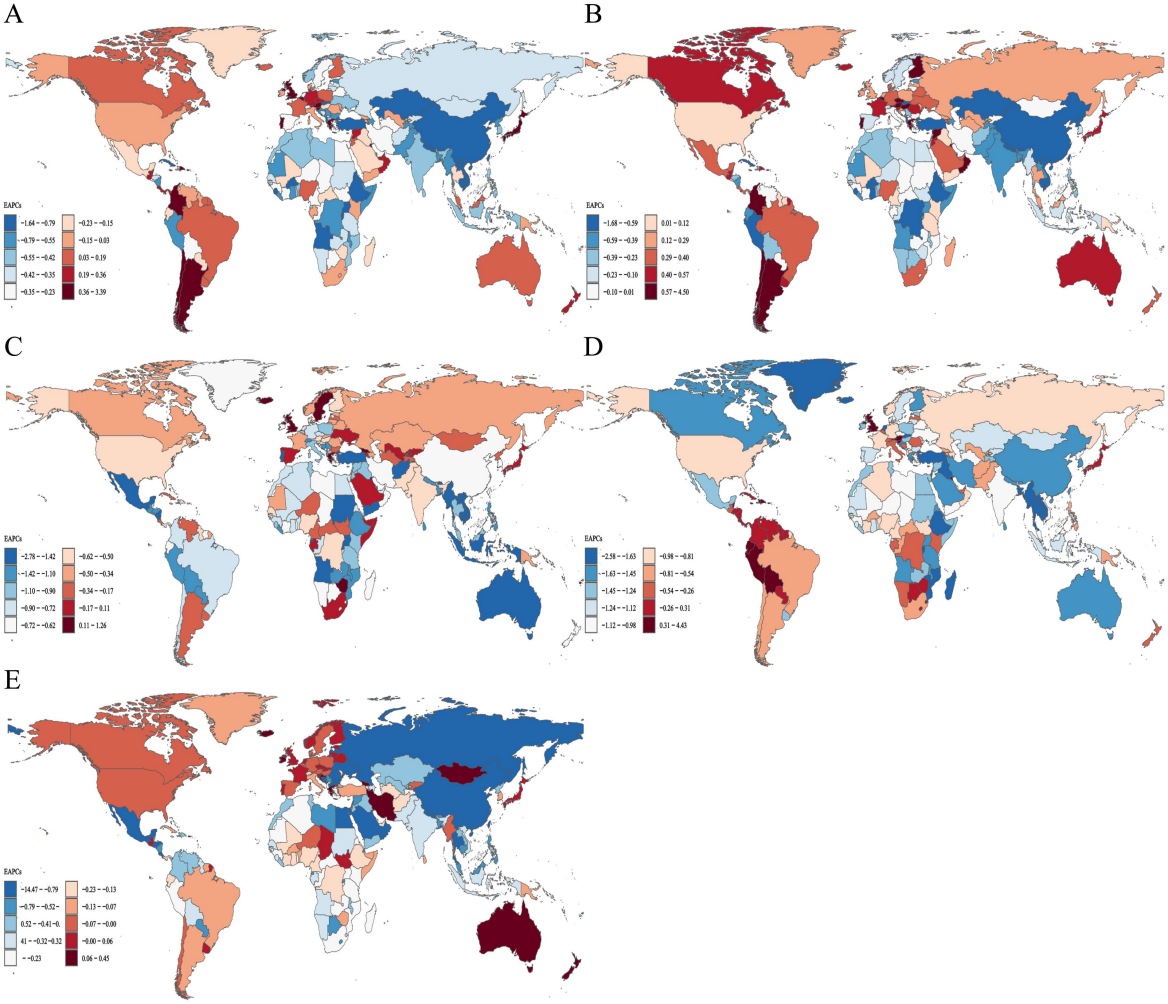
Distribution of ASIR EAPC for neonatal disorders and four etiologies of neonatal disorders globally from 1990 to 2021: **(A)** NDs, **(B)** NPB, **(C)** NE, **(D)** NS, **(E)** HD.

**Figure 2:**
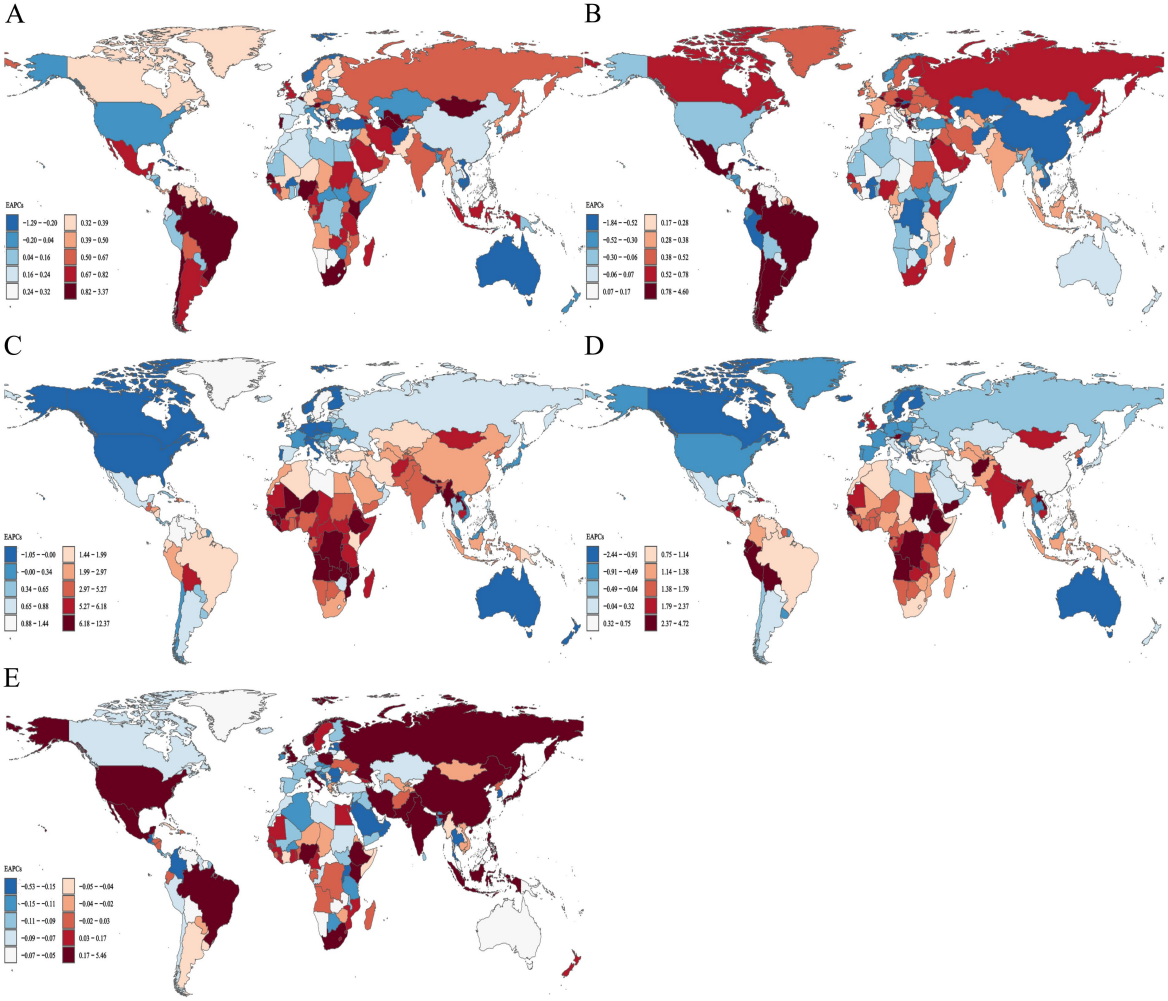
Distribution of ASPR EAPC for neonatal disorders and four etiologies of neonatal disorders globally from 1990 to 2021: **(A)** NDs, **(B)** NPB, **(C)** NE, **(D)** NS, **(E)** HD.

Among the four world regions in 2021, Asia recorded the highest rates of NDs ASIR and ASPR, with values of 468.19 (463.00 to 473.69) and 2280.51 (2081.20 to 2479.06), respectively (Tables 2, 3). From 1990 to 2021, the Americas exhibited an upward trend in NDs ASIR (EAPC = 0.06, 95% CI = 0.04 to 0.08), while Africa, Asia, and Europe showed downward trends, with Asia experiencing the most significant decline in NDs ASIR (EAPC = −0.48, −0.59 to −0.37; Table 2; Figure 1A). During the same period, NDs ASPR in Africa, the Americas, Asia, and Europe demonstrated an upward trend, with Asia showing the largest increase (EAPC = 0.71,0.68 to 0.74) and Europe the smallest (EAPC = 0.31,0.28 to 0.34; Table 3; Figure 2A). In 2021, Africa had the highest ASIR for NE, NS, and HD, with rates of 25.06 (24.68 to 25.47), 80.85 (78.16 to 83.78), and 12.00 (9.24 to 15.50), respectively (Table 2). The ASIR for HD was also highest in Africa, at 27.91 (25.61 to 30.49). In the same year, Asia had the highest ASIR for NPB, at 388.08 (383.59 to 393.39). Additionally, the ASPR for NPB and NE in Asia were the highest recorded, at 1886.30 (1670.58 to 2110.78) and 256.92 (222.41 to 291.73), respectively. Furthermore, in 2021, the ASPR for NS in Europe reached its peak at 173.55 (133.91 to 217.58). From 1990 to 2021, the NPB ASPR in Africa, the Americas, Asia, and Europe exhibited an upward trend, while the NE ASIR in these regions showed a downward trend, although the ASPR demonstrated an upward trend (Tables 2, 3; Figures 1C, 2B,C).

**Table 2:** The ASIR of neonatal disorders and four etiologies of neonatal disorders globally, in four major regions, 21 regions, and five different SDI countries in 2021, and the EAPC trends of ASIR for neonatal disorders and four etiologies of neonatal disorders globally, in four major regions, 21 regions, and five different SDI countries from 1990 to 2021.

Location	2021 ASR					1990–2021 EAPC				
	NDs	NPB	NE	NS	HD	NDs	NPB	NE	NS	HD
Global	437.43 (433.20 to 441.95)	348.41 (345.58 to 351.26)	17.16 (16.94 to 17.41)	62.70 (61.18 to 64.48)	9.16 (6.94 to 12.07)	−0.36 (−0.44 to −0.28)	−0.27 (−0.34 to −0.20)	−0.56 (−0.62 to −0.50)	−0.80 (−0.98 to −0.62)	0.02 (−0.05 to 0.08)
Four world regions										
Africa	455.16 (448.92 to 461.54)	337.25 (333.21 to 341.40)	25.06 (24.68 to 25.47)	80.85 (78.16 to 83.78)	12.00 (9.24 to 15.50)	−0.39 (−0.46 to −0.32)	−0.12 (−0.15 to −0.10)	−0.84 (−0.98 to −0.70)	−1.18 (−1.39 to −0.96)	−0.20 (−0.23 to −0.16)
America	316.02 (312.39 to 320.3)	266.3 (263.28 to 269.63)	12.2 (12.06 to 12.33)	29.28 (28.57 to 30.06)	8.24 (5.94 to 11.02)	0.06 (0.04 to 0.08)	0.22 (0.20 to 0.24)	−1.01 (−1.04 to −0.99)	−0.56 (−0.71 to −0.41)	−0.48 (−0.51 to −0.45)
Asia	468.19 (463.00 to 473.69)	388.08 (383.59 to 393.39)	14.08 (13.73 to 14.44)	57.74 (55.97 to 59.49)	8.30 (6.26 to 11.03)	−0.48 (−0.59 to −0.37)	−0.40 (−0.51 to −0.29)	−0.86 (−0.89 to −0.83)	−0.94 (−1.11 to −0.78)	0.13 (0.03 to 0.24)
Europe	313.04 (306.12 to 319.58)	238.45 (232.69 to 243.87)	7.95 (7.83 to 8.08)	64.19 (61.77 to 66.86)	2.45 (1.34 to 4.41)	−0.24 (−0.27 to −0.20)	0.06 (−0.00 to 0.13)	−0.62 (−0.69 to −0.55)	−0.87 (−0.97 to −0.77)	−3.84 (−4.38 to −3.29)
SDI										
High SDl	281.79 (277.77 to 286.03)	244.32 (240.68 to 247.79)	7.18 (7.02 to 7.33)	29.3 (28.53 to 30.05)	0.99 (0.23 to 2.75)	0.13 (0.06 to 0.19)	0.23 (0.16 to 0.31)	−0.35 (−0.39 to −0.31)	−0.47 (−0.53 to −0.41)	−1.58 (−1.88 to −1.29)
High-middle SDl	279.56 (275.02 to 284.6)	216.68 (212.93 to 221.01)	11.72 (11.42 to 12.04)	48.19 (46.48 to 49.94)	2.97 (1.81 to 4.87)	−0.59 (−0.64 to −0.54)	−0.42 (−0.49 to −0.35)	−0.53 (−0.59 to −0.47)	−1.13 (−1.32 to −0.94)	−2.47 (−2.91 to −2.03)
Middle SDl	364.1 (360.14 to 368.65)	291.08 (288.12 to 293.93)	15.05 (14.77 to 15.33)	50.44 (49.00 to 52.15)	7.52 (5.67 to 10.03)	−0.17 (−0.22 to −0.13)	−0.06 (−0.10 to −0.02)	−0.75 (−0.80 to −0.71)	−0.58 (−0.77 to −0.39)	−0.11 (−0.20 to −0.02)
Low-middle SDl	513.18(505.56 to 521.31)	416.03(409.36 to 423.17)	15.14(14.82 to 15.45)	70.4(68.10 to 72.92)	11.62(8.81 to 15.16)	−0.72(−0.79 to −0.66)	−0.66(−0.73 to −0.59)	−0.95 (−0.97 to −0.93)	−1.09 (−1.22 to −0.96)	−0.03 (−0.05 to −0.02)
Low SDI	516.75(509.51 to 524.01)	398.92(393.65 to 404.21)	25.84(25.47 to 26.22)	79.67(77.16 to 82.49)	12.32(9.50 to 16.09)	−0.61(−0.67 to −0.54)	−0.47(−0.51 to −0.43)	−0.87 (−1.01 to −0.74)	−1.19 (−1.39 to −1.00)	−0.01 (−0.02 to 0.01)
Regions										
Andean Latin America	280.99(268.76 to 293.10)	220.67(209.00 to 233.23)	14.96(14.41 to 15.53)	28.46(26.68 to 30.31)	16.91(12.92 to 21.95)	−0.48(−0.58 to −0.38)	−0.63(−0.76 to −0.51)	−1.09(−1.15 to −1.03)	1.05 (0.78 to 1.32)	−0.29 (−0.31 to −0.27)
Australasia	236.18(219.57 to 252.42)	204.7(188.78 to 220.59)	5.26(5.05 to 5.48)	25.3(22.87 to 28.29)	0.92(0.16 to 2.68)	0.13(0.08 to 0.18)	0.42 (0.37 to 0.47)	−1.29(−1.39 to −1.20)	−1.35 (−1.48 to −1.21)	0.16 (0.14 to 0.18)
Caribbean	459.9(441.51 to 474.92)	387.28(370.42 to 402.29)	23.49 (22.71 to 24.33)	38.87 (36.64 to 41.26)	10.26(7.70 to 13.63)	0.18(0.14 to 0.21)	0.18(0.13 to 0.24)	−0.23(−0.31 to −0.16)	0.36 (0.14 to 0.58)	0.11 (0.07 to 0.15)
Central Asia	282.39(272.10 to 293.7)	184.81(176.31 to 194.05)	17.35(16.89 to 17.83)	63.98(60.56 to 67.30)	16.25(12.05 to 22.10)	−0.40(−0.45 to −0.34)	−0.21(−0.24 to −0.18)	−0.15(−0.22 to −0.07)	−0.95 (−1.15 to −0.76)	−0.29 (−0.38 to −0.20)
Central Europe	329.03(322.22 to 336.57)	222.41(216.78 to 228.47)	11.28(11.09 to 11.48)	93.32(89.57 to 97.67)	2.02(1.19 to 3.83)	−0.30(−0.35 to −0.24)	0.22(0.19 to 0.25)	−0.80(−0.83 to −0.77)	−1.02 (−1.13 to −0.92)	−5.25 (−6.02 to −4.47)
Central Latin America	298.53(292.11 to 305.71)	235.71(230.02 to 241.72)	18.12(17.91 to 18.35)	33.61(32.54 to 34.75)	11.09(8.19 to 14.78)	0.07(0.04 to 0.10)	0.40(0.35 to 0.44)	−1.26(−1.30 to −1.22)	−0.74 (−0.93 to −0.56)	−0.88 (−0.90 to −0.87)
Central Sub-Saharan Africa	358.43(342.51 to 376.44)	256.58(242.96 to 270.62)	21.27(20.42 to 22.24)	69.54(64.14 to 75.74)	11.05(8.43 to 14.30)	−0.63 (−0.71 to −0.56)	−0.63 (−0.67 to −0.58)	−0.91 (−1.16 to −0.66)	−0.64 (−0.95 to −0.32)	−0.26 (−0.29 to −0.22)
East Asia	180.32(176.72 to 184.05)	147.31(144.22 to 150.85)	14.01(13.25 to 14.75)	17.76(17.22 to 18.31)	1.23(0.48 to 2.98)	−1.40 (−1.51 to −1.29)	−1.45 (−1.60 to −1.30)	−0.63 (−0.71 to −0.55)	−1.50 (−1.64 to −1.36)	−1.56 (−1.83 to −1.30)
Eastern Europe	293.51(285.08 to 303.39)	181.07(177.60 to 184.72)	10.32(9.91 to 10.77)	98.4 (90.71 to 107.14)	3.71 (2.61 to 5.57)	−0.36 (−0.42 to −0.30)	0.33 (0.30 to 0.35)	−0.36 (−0.43 to −0.28)	−0.91 (−1.04 to −0.78)	−5.81 (−6.78 to −4.83)
Eastern Sub-Saharan Africa	488.15(479.00 to 498.11)	352.54(344.84 to 360.90)	34.57(33.84 to 35.28)	93.14 (89.04 to 97.67)	7.89 (6.01 to 10.48)	−0.59 (−0.66 to −0.52)	−0.24 (−0.28 to −0.21)	−1.06 (−1.22 to −0.89)	−1.49 (−1.65 to −1.32)	−0.26 (−0.30 to −0.23)
High-income Asia Pacific	200.13 (194.99 to 206.1)	174.62 (169.73 to 180.26)	9.31 (9.18 to 9.45)	15.33 (14.56 to 16.17)	0.87 (0.12 to 2.61)	0.11 (0.05 to 0.16)	0.33 (0.27 to 0.40)	−0.29 (−0.36 to −0.21)	−1.54 (−1.66 to −1.42)	−0.02 (−0.04 to 0.01)
High-income North America	298.46 (293.77 to 303.56)	275.06 (270.45 to 280.05)	6.47 (6.13 to 6.8)	15.91 (15.4 to 16.42)	1.02 (0.26 to 2.77)	−0.02 (−0.16 to 0.12)	0.06 (−0.10 to 0.21)	−0.50 (−0.52 to −0.47)	−0.96 (−0.98 to −0.94)	−0.03 (−0.08 to 0.03)
North Africa and Middle East	430.29 (418.97 to 441.22)	357.05 (346.79 to 366.15)	8.94 (8.76 to 9.13)	51.46 (49.37 to 53.41)	12.83 (9.44 to 17.40)	−0.17 (−0.21 to −0.13)	0.09 (0.07 to 0.11)	−0.96 (−1.05 to −0.86)	−1.35 (−1.57 to −1.13)	−0.30 (−0.40 to −0.21)
Oceania	428.35 (400.18 to 453.54)	362.23 (335.37 to 387.47)	10.82 (10.32 to 11.36)	52.14 (47.66 to 56.79)	3.17 (2.14 to 5.04)	0.01 (−0.02 to 0.04)	0.11 (0.08 to 0.15)	−0.21 (−0.31 to −0.10)	−0.58 (−0.79 to −0.36)	−0.05 (−0.07 to −0.04)
South Asia	645.7 (636.10 to 656.13)	543.78 (535.11 to 553.21)	15 (14.38 to 15.69)	75.38 (72.69 to 78.19)	11.54 (8.80 to 15.1)	−0.60 (−0.66 to −0.53)	−0.53 (−0.60 to −0.46)	−0.65 (−0.72 to −0.58)	−1.08 (−1.16 to −0.99)	−0.01 (−0.07 to 0.04)
Southeast Asia	335.23 (329.22 to 341.77)	261.11 (256.31 to 266.10)	14.14 (13.78 to 14.54)	56.6 (53.26 to 60.32)	3.38 (2.32 to 5.15)	−0.52 (−0.55 to −0.50)	−0.29 (−0.32 to −0.26)	−1.75 (−1.84 to −1.65)	−1.13 (−1.23 to −1.03)	−0.56 (−0.60 to −0.51)
Southern Latin America	305.96 (289.88 to 321.87)	248.22 (233.31 to 264.78)	14.28 (13.74 to 14.88)	39.2 (36.04 to 42.86)	4.26 (2.14 to 7.00)	0.49 (0.45 to 0.54)	0.78 (0.74 to 0.81)	−0.43 (−0.46 to −0.39)	−0.56 (−0.68 to −0.43)	0.12 (0.01 to 0.23)
Southern Sub-Saharan Africa	521.58 (505.60 to 537.09)	338.18 (327.02 to 348.24)	21.48 (21.02 to 21.96)	152.66 (142.08 to 164.34)	9.26 (7.06 to 12.08)	−0.05 (−0.15 to 0.04)	0.16 (0.07 to 0.25)	−0.06 (−0.15 to 0.03)	−0.46 (−0.58 to -0.33)	−0.26 (−0.28 to −0.24)
Tropical Latin America	339.80 (332.82 to 347.26)	284.62 (279.51 to 290.34)	8.26 (8.14 to 8.39)	36.09 (34.92 to 37.34)	10.82 (7.57 to 14.75)	0.13 (0.06 to 0.20)	0.30 (0.19 to 0.40)	−0.80 (−0.92 to −0.67)	−0.62 (−0.82 to −0.41)	−0.16 (−0.19 to −0.13)
Western Europe	293.46 (285.54 to 300.78)	243.88 (236.50 to 250.52)	6.25 (6.14 to 6.35)	42.40 (41.14 to 43.71)	0.94 (0.17 to 2.71)	0.24 (0.23 to 0.26)	0.34 (0.31 to 0.37)	−0.48 (−0.53 to −0.43)	−0.14 (−0.21 to −0.06)	0.00 (−0.01 to 0.02)
Western Sub-Saharan Africa	466.01 (457.62 to 473.91)	348.87 (343.32 to 354.52)	24.03 (23.35 to 24.70)	77.69 (73.76 to 81.94)	15.43 (12.11 to 19.65)	−0.21 (−0.30 to −0.13)	0.01 (−0.03 to 0.05)	−0.58 (−0.66 to −0.50)	−0.95 (−1.22 to −0.68)	−0.11 (−0.14 to −0.07)

**Table 3:** ASPR of neonatal disorders and four etiologies of neonatal disorders in 2021 globally, across four major regions, 21 regions, and five different SDI countries, and trends in EAPC of ASPR of neonatal disorders and four etiologies of neonatal disorders from 1990 to 2021 globally, across four major regions, 21 regions, and five different SDI countries.

Location	2021 ASR					1990–2021 EAPC				
	NDs	NPB	NE	NS	HD	NDs	NPB	NE	NS	HD
Global	2018.39 (1854.6 to 2185.48)	1633.61 (1453.78 to 1818.24)	242.03 (210.3 to 273.72)	125.86 (97.03 to 156.32)	26.12 (23.96 to 28.77)	0.60 (0.58 to 0.62)	0.39 (0.37 to 0.41)	2.16 (2.11 to 2.21)	0.96 (0.89 to 1.03)	2.16 (2.11 to 2.21)
Four World Regions										
Africa	1717.06 (1546.79 to 1882.03)	1329.46 (1136.21 to 1512.5)	220.92 (185.1 to 259.69)	146.89 (113.94 to 183.16)	27.91 (25.61 to 30.49)	0.47 (0.39 to 0.55)	0.05 (0.03 to 0.08)	4.56 (4.20 to 4.92)	1.48 (1.30 to 1.66)	4.56 (4.20 to 4.92)
America	1698.64 (1552.3 to 1842.63)	1370.15 (1198.48 to 1538.17)	233.61 (205.11 to 263.23)	73.56 (56.54 to 92.05)	27.01 (24.84 to 29.65)	0.43 (0.41 to 0.45)	0.36 (0.35 to 0.37)	0.84 (0.71 to 0.96)	0.46 (0.26 to 0.66)	0.84 (0.71 to 0.96)
Asia	2280.51 (2081.2 to 2479.06)	1886.3 (1670.58 to 2110.78)	256.92 (222.41 to 291.73)	121.26 (93.09 to 151.05)	26.87 (24.59 to 29.74)	0.71 (0.68 to 0.74)	0.46 (0.43 to 0.49)	2.67 (2.58 to 2.76)	1.49 (1.40 to 1.58)	2.67 (2.58 to 2.76)
Europe	1491.62 (1378.07 to 1625.25)	1119.43 (979.94 to 1269.45)	188.27 (167.25 to 209.99)	173.55 (133.91 to 217.58)	16.03 (14.65 to 17.88)	0.31 (0.28 to 0.34)	0.41 (0.39 to 0.43)	0.31 (0.24 to 0.37)	−0.17 (−0.33 to −0.01)	0.31 (0.24 to 0.37)
SDI										
High SDl	1535.21 (1392.25 to 1668.87)	1262.83 (1102.19 to 1416.75)	189.24 (167.11 to 212.9)	81.32 (62.69 to 101.9)	6.04 (5.48 to 6.79)	0.25 (0.19 to 0.31)	0.26 (0.19 to 0.33)	0.34 (0.30 to 0.38)	−0.10 (−0.24 to 0.04)	0.34 (0.30 to 0.38)
High-middle SDl	1419.24 (1334.37 to 1515.94)	1010.79 (896.05 to 1130.06)	276.83 (243.01 to 310.24)	119.94 (92.37 to 150.44)	17.71 (16.16 to 19.72)	0.38 (0.34 to 0.41)	0.23 (0.18 to 0.28)	1.48 (1.35 to 1.61)	−0.43 (−0.60 to −0.25)	1.48 (1.35 to 1.61)
Middle SDl	1859.37 (1729.45 to 1998.37)	1436.26 (1284.48 to 1596.45)	293.28 (255.14 to 331.44)	112.68 (86.29 to 140.23)	26.16 (24.02 to 28.91)	0.93 (0.89 to 0.96)	0.66 (0.60 to 0.72)	2.35 (2.23 to 2.48)	1.32 (1.18 to 1.46)	2.35 (2.23 to 2.48)
Low-middle SDl	2643.47 (2373.26 to 2946.65)	2270.83 (1969.31 to 2593.52)	196.92 (167.89 to 227.41)	151.79 (117.61 to 188.01)	35.88 (32.97 to 39.43)	0.30 (0.25 to 0.34)	0.02 (−0.02 to 0.06)	4.39 (4.33 to 4.46)	1.83 (1.73 to 1.93)	4.39 (4.33 to 4.46)
Low SDI	2180.93 (1947.4 to 2447.41)	1808.94 (1557.63 to 2071.07)	207.92 (169.13 to 250.92)	143.13 (110.57 to 177.92)	31.12 (28.48 to 34.04)	0.25 (0.20 to 0.30)	−0.14 (−0.17 to −0.11)	6.82 (6.48 to 7.17)	2.04 (1.81 to 2.26)	6.82 (6.48 to 7.17)
Regions										
Andean Latin America	1416.13 (1282.6 to 1608.35)	1016.08 (864.74 to 1230.8)	286.89 (252.14 to 324.26)	72.79 (55.52 to 91.61)	45.97 (42.37 to 50.2)	0.16 (0.08 to 0.24)	−0.50 (−0.62 to −0.38)	2.98 (2.71 to 3.24)	3.63 (3.36 to 3.90)	2.98 (2.71 to 3.24)
Australasia	1427.45 (1237.48 to 1695.53)	1224.14 (1017.35 to 1504.46)	133.33 (117.9 to 150.94)	70.96 (54.85 to 89.5)	2.07 (1.84 to 2.36)	−0.18 (−0.22 to −0.14)	−0.03 (−0.07 to 0.01)	−0.75 (−0.88 to −0.63)	−1.29 (−1.52 to −1.07)	−0.75 (−0.88 to −0.63)
Caribbean	2147.08 (1873.69 to 2577.77)	1756.25 (1485.04 to 2204.44)	275.72 (242.12 to 310.23)	87.05 (67.16 to 107.74)	37.62 (34.38 to 41.83)	0.20 (0.15 to 0.26)	0.05 (−0.02 to 0.13)	1.02 (0.88 to 1.15)	1.05 (0.84 to 1.26)	1.02 (0.88 to 1.15)
Central Asia	1315.44 (1218.45 to 1439.86)	810.67 (696.02 to 947.8)	302.02 (265.94 to 339.6)	159.46 (122.93 to 198.88)	49.62 (45.27 to 54.38)	0.44 (0.38 to 0.50)	−0.10 (−0.19 to −0.01)	2.28 (2.17 to 2.40)	0.81 (0.66 to 0.97)	2.28 (2.17 to 2.40)
Central Europe	1618.73 (1494.79 to 1747.75)	1085.85 (939.08 to 1238.14)	264.66 (235.22 to 295.66)	254.42 (196.31 to 319.52)	22.56 (20.65 to 25.02)	0.32 (0.27 to 0.37)	0.48 (0.46 to 0.50)	0.42 (0.36 to 0.49)	−0.30 (−0.49 to −0.12)	0.42 (0.36 to 0.49)
Central Latin America	1548.73 (1443.19 to 1673.75)	1076.3 (942.49 to 1212.76)	352.59 (309.56 to 397.76)	85.58 (65.7 to 107.06)	41.11 (37.73 to 45.02)	0.77 (0.74 to 0.79)	0.76 (0.69 to 0.83)	0.92 (0.70 to 1.13)	0.59 (0.33 to 0.86)	0.92 (0.70 to 1.13)
Central Sub-Saharan Africa	1268.77 (1056.61 to 1633.51)	940.62 (718.34 to 1298.83)	185.13 (148.53 to 223.89)	126.14 (98.07 to 157.81)	21.79 (19.97 to 23.96)	0.27 (0.11 to 0.44)	−0.37 (−0.44 to −0.31)	7.58 (6.84 to 8.34)	2.62 (2.30 to 2.94)	7.58 (6.84 to 8.34)
East Asia	1041.82 (993.55 to 1100.3)	651.77 (578.04 to 734.39)	332.07 (290.05 to 375.5)	48.82 (37.43 to 61.1)	12.58 (11.44 to 14.1)	0.20 (0.12 to 0.28)	−0.74 (−0.87 to −0.61)	2.91 (2.65 to 3.17)	0.47 (0.23 to 0.71)	2.91 (2.65 to 3.17)
Eastern Europe	1338.96 (1252.69 to 1438.84)	836.41 (733.32 to 947.1)	221.67 (192.81 to 251.48)	253.3 (194.39 to 317.59)	33.99 (31.03 to 37.91)	0.45 (0.39 to 0.51)	0.63 (0.57 to 0.68)	0.61 (0.50 to 0.72)	−0.14 (−0.30 to 0.02)	0.61 (0.50 to 0.72)
Eastern Sub-Saharan Africa	1835.18 (1652.84 to 2050.52)	1353.15 (1145.4 to 1579.35)	297.04 (240.34 to 358.57)	172.78 (134.02 to 215.28)	22.4 (20.54 to 24.63)	0.63 (0.53 to 0.72)	0.01 (−0.00 to 0.02)	6.17 (5.79 to 6.55)	1.95 (1.70 to 2.20)	6.17 (5.79 to 6.55)
High-income Asia Pacific	1159.05 (1071.12 to 1258.56)	865.59 (756.92 to 982.2)	244.73 (215.85 to 274.27)	47.81 (36.57 to 60.48)	4.08 (3.69 to 4.59)	0.43 (0.38 to 0.49)	0.53 (0.45 to 0.60)	0.33 (0.28 to 0.38)	−0.49 (−0.71 to −0.26)	0.33 (0.28 to 0.38)
High-income North America	1775.81 (1577.86 to 1970.37)	1572.85 (1355.95 to 1780.31)	158.68 (138.27 to 178.9)	42.93 (33.06 to 53.79)	5.3 (4.78 to 5.98)	−0.07 (−0.19 to 0.04)	−0.04 (−0.18 to 0.09)	−0.17 (−0.19 to −0.15)	−0.80 (−0.89 to −0.72)	−0.17 (−0.19 to −0.15)
North Africa and Middle East	1935.58 (1736.23 to 2169.63)	1607.2 (1385.47 to 1856.56)	167.53 (148.32 to 187.48)	128.67 (98.72 to 160.42)	39.52 (36.23 to 43.43)	0.33 (0.30 to 0.36)	0.16 (0.10 to 0.22)	2.08 (1.90 to 2.27)	0.77 (0.46 to 1.08)	2.08 (1.90 to 2.27)
Oceania	1869.7 (1521.3 to 2532.48)	1650.53 (1294.57 to 2324.25)	103.28 (84.66 to 123.61)	100.53 (76.72 to 124.53)	20.54 (18.56 to 22.91)	0.14 (0.11 to 0.17)	0.05 (0.03 to 0.07)	1.70 (1.43 to 1.96)	0.50 (0.34 to 0.65)	1.70 (1.43 to 1.96)
South Asia	3521.06 (3142.48 to 3910.85)	3113.16 (2715.63 to 3514.86)	217.95 (185.47 to 251.69)	169.99 (131.24 to 210.84)	37.83 (34.67 to 41.65)	0.47 (0.43 to 0.52)	0.23 (0.19 to 0.28)	5.30 (5.22 to 5.39)	1.84 (1.73 to 1.95)	5.30 (5.22 to 5.39)
Southeast Asia	1608.9 (1473.91 to 1745.61)	1217.58 (1061.66 to 1381.91)	248.5 (217.35 to 280.67)	130.78 (100.38 to 163.27)	18.74 (17.12 to 20.95)	0.25 (0.22 to 0.28)	0.02 (−0.03 to 0.06)	1.28 (1.15 to 1.41)	0.75 (0.61 to 0.88)	1.28 (1.15 to 1.41)
Southern Latin America	1699.83 (1540.06 to 1931.54)	1270.88 (1077.66 to 1519.83)	314.8 (280.98 to 349.95)	103.88 (79.69 to 130.55)	17.42 (15.75 to 19.35)	0.81 (0.78 to 0.84)	0.96 (0.91 to 1.01)	0.58 (0.52 to 0.64)	0.07 (−0.14 to 0.28)	0.58 (0.52 to 0.64)
Southern Sub-Saharan Africa	1968.48 (1775.27 to 2197.95)	1400.76 (1190.2 to 1643.63)	278.27 (238.4 to 321.1)	279.44 (214.23 to 346.37)	23.34 (21.39 to 25.72)	0.78 (0.68 to 0.88)	0.47 (0.45 to 0.49)	2.57 (2.04 to 3.11)	1.16 (0.82 to 1.49)	2.57 (2.04 to 3.11)
Tropical Latin America	1744.56 (1559.79 to 1925.34)	1461.38 (1259.6 to 1662.98)	158.8 (137.83 to 180.55)	93.17 (71.45 to 116.26)	36.7 (33.74 to 40.16)	1.26 (1.17 to 1.36)	1.26 (1.10 to 1.41)	1.62 (1.37 to 1.88)	0.85 (0.57 to 1.13)	1.62 (1.37 to 1.88)
Western Europe	1431.71 (1301.56 to 1579.33)	1150.89 (999.78 to 1320.39)	163.79 (145.86 to 182.91)	117.36 (90.57 to 146.97)	3.93 (3.55 to 4.42)	0.37 (0.34 to 0.41)	0.45 (0.43 to 0.47)	0.04 (−0.02 to 0.09)	0.12 (−0.04 to 0.28)	0.04 (−0.02 to 0.09)
Western Sub-Saharan Africa	1596.29 (1425.54 to 1771.33)	1272.33 (1080.22 to 1456.19)	185.71 (155.42 to 218.81)	120.45 (93.77 to 150.18)	24.68 (22.66 to 26.92)	0.59 (0.52 to 0.66)	0.21 (0.19 to 0.23)	5.05 (4.58 to 5.54)	1.51 (1.23 to 1.78)	5.05 (4.58 to 5.54)

#### 3.2.2 Deaths

**Figure 3:**
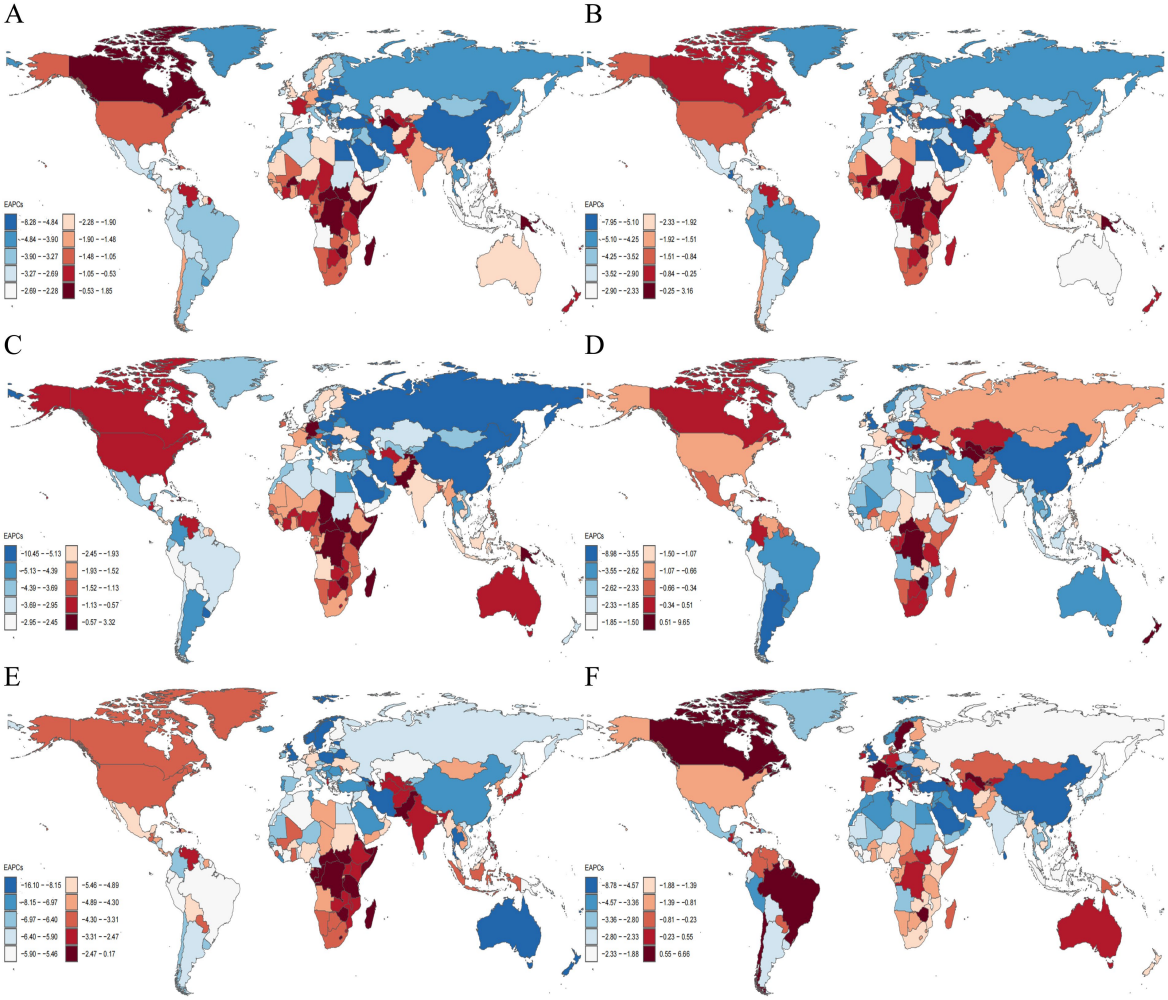
Distribution of ASDR EAPC for neonatal disorders and five etiologies of neonatal disorders globally from 1990 to 2021: **(A)** NDs, **(B)** NPB, **(C)** NE, **(D)** NS, **(E)** HD, **(F)** Other neonatal disorders.

In 2021, Africa recorded the highest ASDR for NDs at 44.12 (36.84–52.33; Table 4). Between 1990 and 2021, the ASDR for NDs in Africa, the Americas, Asia, and Europe exhibited a downward trend, with Europe experiencing the most significant decline (EAPC = −4.19, −4.29 to −4.10), while Africa had the smallest decline (EAPC = −1.13, −1.22 to −1.04; Table 4; Figure 3A). In 2021, Africa also had the highest ASDR for neonatal conditions, including NPB, NE, NS, and other neonatal disorders, with rates of 15.64 (12.91–18.55), 16.31 (13.44–19.60), 6.64 (5.48–8.00), and 5.11 (3.02–6.72), respectively. Conversely, Asia reported the highest ASDR for HD, at 0.81 (0.61–1.04; Table 4). Overall, from 1990 to 2021, the ASDR for five NDs etiologies across Africa, the Americas, Asia, and Europe demonstrated a consistent declining trend (Table 4; Figures 3B–F).

**Table 4:** The ASDR of neonatal disorders and five etiologies of neonatal disorders globally, in four major regions, 21 regions, and five different SDI countries in 2021, and the EAPC trends of ASDR for neonatal disorders and five etiologies of neonatal disorders globally, in four major regions, 21 regions, and five different SDI countries from 1990 to 2021.

Location	2021 ASR						1990–2021 EAPC					
	NDs	NPB	NE	NS	HD	Other neonatal disorders	NDs	NPB	NE	NS	HD	Other neonatal disorders
Global	29.57 (25.37 to 34.26)	11.94 (10.05 to 14.16)	9.75 (8.26 to 11.71)	3.76 (3.18 to 4.40)	0.54 (0.42 to 0.69)	3.58 (2.58 to 4.34)	−1.49 (−1.57 to −1.40)	−1.67 (−1.72 to −1.62)	−1.24 (−1.37 to −1.10)	−0.93 (−1.06 to −0.80)	−3.44 (−3.57 to −3.31)	−1.63 (−1.76 to −1.50)
Four World Regions												
Africa	44.12 (36.84 to 52.33)	15.64 (12.91 to 18.55)	16.31 (13.44 to 19.60)	6.64 (5.48 to 8.00)	0.41 (0.31 to 0.58)	5.11 (3.02 to 6.72)	−1.13 (−1.22 to −1.04)	−1.05 (−1.12 to −0.97)	−0.90 (−1.05 to −0.75)	−1.10 (−1.18 to −1.02)	−4.06 (−4.23 to −3.90)	−1.70 (−1.81 to −1.60)
America	11.07 (9.06 to 13.38)	5.13 (4.22 to 6.17)	2.32 (1.88 to 2.83)	2.12 (1.68 to 2.59)	0.06 (0.05 to 0.08)	1.44 (1.20 to 1.74)	−2.64 (−2.71 to −2.57)	−3.28 (−3.36 to −3.20)	−2.72 (−2.79 to −2.66)	−1.65 (−1.89 to −1.40)	−5.04 (−5.14 to −4.94)	−0.60 (−0.77 to −0.42)
Asia	27.08 (23.15 to 31.84)	12.16 (9.92 to 14.62)	8.11 (6.54 to 10.10)	2.58 (2.14 to 3.06)	0.81 (0.61 to 1.04)	3.42 (2.68 to 4.34)	−1.99 (−2.11 to −1.87)	−1.91 (−2.00 to −1.82)	−2.04 (−2.21 to −1.86)	−1.68 (−1.84 to −1.52)	−3.04 (−3.19 to −2.89)	−2.09 (−2.26 to −1.93)
Europe	4.77 (4.13 to 5.51)	2.77 (2.40 to 3.18)	0.86 (0.74 to 1.00)	0.59 (0.50 to 0.69)	0.04 (0.03 to 0.05)	0.52 (0.42 to 0.62)	−4.19 (−4.29 to −4.10)	−4.35 (−4.45 to −4.25)	−4.66 (−4.80 to −4.53)	−2.47 (−2.63 to −2.31)	−6.89 (−7.06 to −6.71)	−3.52 (−3.69 to −3.35)
SDI												
High SDl	3.88 (3.48 to 4.24)	2.39 (2.13 to 2.61)	0.66 (0.58 to 0.72)	0.3 (0.26 to 0.33)	0.01 (0.01 to 0.01)	0.53 (0.47 to 0.59)	−2.55 (−2.65 to −2.44)	−2.67 (−2.79 to −2.55)	−2.59 (−2.69 to −2.49)	−3.09 (−3.32 to −2.86)	−5.35 (−5.87 to −4.84)	−1.33 (−1.49 to −1.16)
High-middle SDl	5.62 (4.93 to 6.31)	3.14 (2.74 to 3.55)	1.09 (0.93 to 1.27)	0.72 (0.62 to 0.84)	0.06 (0.05 to 0.08)	0.61 (0.49 to 0.74)	−4.63 (−4.84 to −4.42)	−4.31 (−4.50 to −4.11)	−6.17 (−6.48 to −5.85)	−2.42 (−2.59 to −2.25)	−6.93 (−7.17 to −6.70)	−3.94 (−4.17 to −3.70)
Middle SDl	16.26 (13.96 to 18.98)	8.06 (6.75 to 9.37)	4.06 (3.42 to 4.86)	2.18 (1.81 to 2.62)	0.24 (0.19 to 0.30)	1.72 (1.42 to 2.19)	−2.64 (−2.78 to −2.51)	−2.43 (−2.55 to −2.31)	−3.48 (−3.66 to −3.29)	−1.24 (−1.48 to −1.00)	−4.84 (−4.97 to −4.71)	−2.30 (−2.41 to −2.19)
Low-middle SDl	37.65 (32.30 to 44.07)	16.07 (13.13 to 19.10)	12.07 (9.90 to 14.49)	4.05 (3.32 to 4.89)	0.90 (0.67 to 1.17)	4.56 (3.51 to 5.74)	−1.72 (−1.79 to −1.66)	−1.77 (−1.84 to −1.71)	−1.28 (−1.42 to −1.13)	−1.63 (−1.71 to −1.55)	−3.46 (−3.58 to −3.34)	−2.21 (−2.32 to −2.11)
Low SDI	48.03 (39.93 to 57.41)	16.62 (13.66 to 19.98)	17.77 (14.77 to 21.58)	6.85 (5.52 to 8.54)	0.74 (0.57 to 1.10)	6.05 (3.57 to 8.12)	−1.12 (−1.20 to −1.05)	−1.13 (−1.21 to −1.05)	−0.79 (−0.92 to −0.67)	−1.13 (−1.19 to −1.07)	−3.25 (−3.41 to −3.09)	−1.60 (−1.69 to −1.51)
Regions												
Andean Latin America	12.41 (9.79 to 15.32)	5.01 (3.85 to 6.33)	3.18 (2.32 to 4.11)	3.4 (2.51 to 4.53)	0.07 (0.05 to 0.09)	0.74 (0.57 to 0.97)	−3.03 (−3.20 to −2.85)	−3.59 (−3.81 to −3.38)	−3.09 (−3.30 to −2.89)	−1.82 (−2.05 to −1.59)	−5.32 (−5.52 to −5.13)	−3.16 (−3.50 to −2.82)
Australasia	3.06 (2.58 to 3.65)	1.67 (1.42 to 1.97)	0.75 (0.62 to 0.91)	0.17 (0.14 to 0.21)	0.00 (0.00 to 0.00)	0.47 (0.38 to 0.57)	−1.86 (−2.06 to −1.66)	−2.34 (−2.82 to −1.85)	−1.01 (−1.63 to −0.39)	−2.32 (−3.08 to −1.56)	−10.89 (−14.16 to −7.50)	0.12 (−1.34 to 1.60)
Caribbean	27.66 (22.37 to 33.58)	10.60 (8.32 to 13.61)	8.03 (6.11 to 10.63)	5.81 (4.32 to 7.62)	0.23 (0.15 to 0.37)	2.98 (2.08 to 4.14)	−0.59 (−0.69 to −0.48)	−1.26 (−1.37 to −1.14)	−0.32 (−0.45 to −0.18)	0.36 (0.22 to 0.50)	−3.04 (−3.15 to −2.92)	0.02 (−0.14 to 0.18)
Central Asia	15.71 (13.57 to 18.38)	7.12 (5.96 to 8.41)	4.64 (3.91 to 5.51)	1.33 (1.12 to 1.6)	0.10 (0.08 to 0.13)	2.52 (2.07 to 3.08)	−1.40 (−1.79 to −1.01)	−0.60 (−0.88 to −0.33)	−3.06 (−3.71 to −2.39)	0.43 (0.08 to 0.78)	−3.80 (−4.43 to −3.17)	0.72 (0.39 to 1.05)
Central Europe	3.98 (3.39 to 4.65)	2.84 (2.39 to 3.30)	0.50 (0.43 to 0.59)	0.17 (0.14 to 0.20)	0.02 (0.02 to 0.03)	0.45 (0.36 to 0.55)	−4.97 (−5.14 to −4.79)	−4.50 (−4.69 to −4.31)	−6.43 (−6.68 to −6.18)	−6.14 (−6.85 to −5.43)	−7.35 (−7.57 to −7.14)	−5.10 (−5.53 to −4.67)
Central Latin America	12.66 (10.01 to 15.94)	6.10 (4.79 to 7.72)	2.42 (1.92 to 3.07)	2.98 (2.35 to 3.76)	0.08 (0.06 to 0.1)	1.08 (0.84 to 1.4)	−2.66 (−2.76 to −2.56)	−3.07 (−3.21 to −2.93)	−3.45 (−3.65 to −3.24)	−0.65 (−0.81 to −0.50)	−5.14 (−5.44 to −4.83)	−1.82 (−1.95 to −1.69)
Central Sub-Saharan Africa	37.97 (29.61 to 45.41)	10.59 (8.17 to 13.44)	17.66 (13.95 to 22.43)	3.40 (1.9 to 5.54)	0.18 (0.10 to 0.37)	6.14 (2.46 to 10.96)	−0.60 (−0.86 to −0.33)	−0.65 (−0.89 to −0.42)	−0.67 (−0.93 to −0.41)	0.18 (−0.16 to 0.52)	−1.92 (−2.29 to −1.54)	−0.63 (−0.93 to −0.32)
East Asia	5.19 (4.46 to 6.11)	2.69 (2.25 to 3.21)	1.75 (1.44 to 2.11)	0.25 (0.19 to 0.32)	0.09 (0.07 to 0.12)	0.41 (0.32 to 0.53)	−5.53 (−5.92 to −5.15)	−4.59 (−4.93 to −4.24)	−6.71 (−7.24 to −6.17)	−3.59 (−3.79 to −3.39)	−7.73 (−7.99 to −7.48)	−5.02 (−5.31 to −4.73)
Eastern Europe	4.39 (3.93 to 4.87)	2.27 (2.03 to 2.52)	0.82 (0.73 to 0.91)	1.05 (0.93 to 1.17)	0.05 (0.04 to 0.06)	0.2 (0.18 to 0.23)	−4.33 (−4.62 to −4.04)	−4.12 (−4.47 to −3.77)	−6.52 (−6.88 to −6.16)	−0.88 (−1.35 to −0.42)	−5.73 (−5.94 to −5.51)	−2.70 (−3.03 to −2.38)
Eastern Sub-Saharan Africa	43.06 (34.61 to 53.1)	11.94 (9.45 to 14.95)	15.39 (12.33 to 19.21)	8.01 (6.24 to 10.32)	0.54 (0.38 to 0.89)	7.18 (3.84 to 10.27)	−1.42 (−1.53 to −1.31)	−1.38 (−1.44 to −1.32)	−1.27 (−1.45 to −1.08)	−1.46 (−1.54 to −1.37)	−2.76 (−2.89 to −2.63)	−1.62 (−1.73 to −1.52)
High-income Asia Pacific	1.31 (1.19 to 1.45)	0.69 (0.62 to 0.77)	0.24 (0.21 to 0.26)	0.14 (0.12 to 0.17)	0.01 (0.01 to 0.01)	0.24 (0.20 to 0.27)	−4.08 (−4.24 to −3.91)	−4.36 (−4.53 to −4.18)	−4.09 (−4.39 to −3.78)	−4.13 (−4.33 to −3.93)	−3.53 (−4.36 to −2.69)	−3.03 (−3.20 to −2.86)
High-income North America	5.36 (4.79 to 5.96)	3.34 (2.98 to 3.72)	0.82 (0.73 to 0.91)	0.37 (0.33 to 0.41)	0.01 (0.01 to 0.01)	0.83 (0.73 to 0.92)	−1.22 (−1.39 to −1.06)	−1.39 (−1.56 to −1.22)	−0.93 (−1.06 to −0.80)	−0.66 (−0.99 to −0.32)	−3.73 (−4.44 to −3.02)	−0.98 (−1.25 to −0.70)
North Africa and Middle East	15.12 (12.95 to 17.52)	9.17 (7.71 to 10.79)	2.90 (2.28 to 3.56)	0.89 (0.68 to 1.13)	0.24 (0.18 to 0.38)	1.91 (1.19 to 2.56)	−3.61 (−3.76 to −3.45)	−3.80 (−4.00 to −3.60)	−2.80 (−2.93 to −2.66)	−2.94 (−3.06 to −2.81)	−5.29 (−5.44 to −5.13)	−3.65 (−3.77 to −3.53)
Oceania	22.23 (17.8 to 27.15)	10.73 (8.24 to 13.65)	4.94 (3.49 to 6.75)	1.61 (0.99 to 2.46)	0.02 (0.01 to 0.07)	4.95 (3.41 to 7.1)	−0.38 (−0.47 to −0.29)	−0.18 (−0.34 to −0.02)	−0.62 (−0.75 to −0.49)	−0.14 (−0.41 to 0.13)	−5.60 (−5.86 to −5.34)	−0.58 (−0.68 to −0.47)
South Asia	42.03 (35.57 to 49.84)	18.54 (14.77 to 22.83)	12.94 (10.23 to 16.84)	3.54 (2.86 to 4.30)	1.44 (1.06 to 1.87)	5.57 (4.22 to 7.14)	−1.61 (−1.66 to −1.55)	−1.55 (−1.66 to −1.45)	−1.25 (−1.37 to −1.14)	−1.72 (−1.80 to −1.64)	−2.64 (−2.81 to −2.47)	−2.12 (−2.20 to −2.04)
Southeast Asia	18.02 (15.15 to 21.06)	8.13 (6.64 to 9.61)	4.52 (3.21 to 5.63)	3.56 (2.72 to 4.87)	0.25 (0.20 to 0.32)	1.56 (1.08 to 2.69)	−2.21 (−2.30 to −2.12)	−2.26 (−2.31 to −2.20)	−2.20 (−2.35 to −2.05)	−1.97 (−2.12 to −1.82)	−4.72 (−4.78 to −4.67)	−1.90 (−1.94 to −1.86)
Southern Latin America	7.87 (6.18 to 9.82)	5.07 (4.00 to 6.32)	0.85 (0.67 to 1.07)	1.08 (0.84 to 1.37)	0.03 (0.02 to 0.04)	0.84 (0.66 to 1.06)	−3.32 (−3.44 to −3.20)	−3.18 (−3.29 to −3.06)	−4.66 (−4.90 to −4.41)	−3.54 (−3.81 to −3.27)	−6.25 (−6.55 to −5.95)	−1.73 (−1.89 to −1.56)
Southern Sub-Saharan Africa	36.83 (30.59 to 44.54)	15.61 (12.51 to 19.38)	8.28 (6.39 to 10.82)	4.31 (3.43 to 5.34)	0.33 (0.24 to 0.42)	8.31 (6.40 to 10.84)	−0.65 (−0.75 to −0.55)	−0.29 (−0.40 to −0.19)	−1.15 (−1.29 to −1.02)	0.46 (0.27 to 0.65)	−2.88 (−3.00 to −2.75)	−1.11 (−1.19 to −1.02)
Tropical Latin America	12.51 (10.08 to 15.53)	4.94 (3.98 to 6.07)	2.70 (2.18 to 3.34)	2.14 (1.69 to 2.67)	0.08 (0.06 to 0.11)	2.64 (2.14 to 3.29)	−3.52 (−3.74 to −3.29)	−4.92 (−5.04 to −4.79)	−2.95 (−3.23 to −2.68)	−3.24 (−3.78 to −2.70)	−5.56 (−5.80 to −5.31)	0.70 (0.20 to 1.20)
Western Europe	3.17 (2.78 to 3.54)	1.86 (1.64 to 2.07)	0.65 (0.56 to 0.74)	0.24 (0.20 to 0.27)	0.01 (0.01 to 0.01)	0.41 (0.35 to 0.48)	−2.36 (−2.51 to −2.20)	−2.80 (−2.97 to −2.64)	−2.19 (−2.41 to −1.97)	−1.71 (−1.94 to −1.48)	−6.25 (−6.93 to −5.57)	−0.13 (−0.39 to 0.13)
Western Sub-Saharan Africa	56.74 (48.04 to 66.23)	21.57 (17.90 to 25.89)	22.11 (17.98 to 26.42)	8.55 (7.07 to 10.13)	0.44 (0.33 to 0.61)	4.07 (2.81 to 5.15)	−0.89 (−0.97 to −0.82)	−0.30 (−0.40 to −0.20)	−0.95 (−1.06 to −0.84)	−1.26 (−1.33 to −1.19)	−5.20 (−5.49 to −4.91)	−1.68 (−1.77 to −1.60)

#### 3.2.3 DALYs

**Figure 4:**
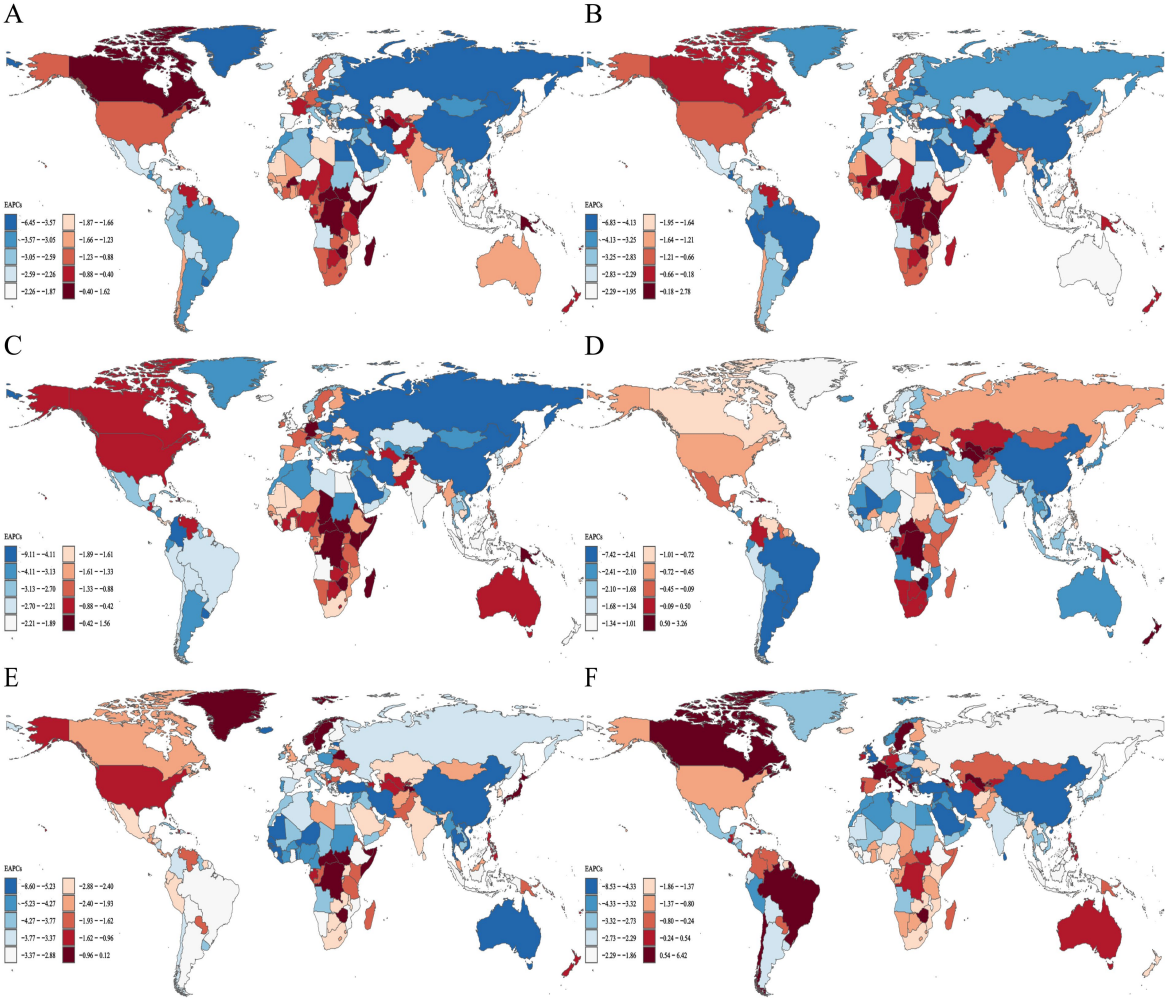
Distribution of EAPC in age-standardized DALYs rates for neonatal disorders and five etiologies of neonatal disorders globally from 1990 to 2021: **(A)** NDs, **(B)** NPB, **(C)** NE, **(D)** NS, **(E)** HD, **(F)** Other neonatal disorders.

In 2021, Africa recorded the highest age-standardized DALYs rate for NDs at 4194.32 (3531.46–4928.36; Table 5). Between 1990 and 2021, the age-standardized DALYs rates for NDs in Africa, the Americas, Asia, and Europe exhibited a downward trend, with Europe demonstrating the most significant decline (EAPC = −3.40, −3.46 to −3.34) and Africa showing the least decline (EAPC = −1.03, −1.10 to −0.95; Table 5; Figure 4A). In 2021, Africa also had the highest age-standardized DALYs rates for neonatal diseases, including NPB, NE, NS, and other neonatal disorders, with rates of 1518.78 (1293.20 to 1785.03), 1525.94 (1267.97 to 1828.71), 637.91 (527.88 to 760.43), and 463.66 (275.18 to 609.21), respectively. Furthermore, Asia reported the highest age-standardized DALYs rate for HD, at 81.70 (63.28 to 101.51; Table 5). Overall, from 1990 to 2021, the age-standardized DALYs rates for five NDs etiologies across Africa, the Americas, Asia, and Europe displayed a consistent downward trend (Table 5; Figures 4B–F).

**Table 5:** Age-standardized DALYs rates for neonatal disorders and four etiologies of neonatal disorders globally, in four major regions, 21 regions, and five different SDI countries in 2021, and the EAPC trends of age-standardized DALYs rates for neonatal disorders and four etiologies of neonatal disorders globally, in four major regions, 21 regions, and five different SDI countries from 1990 to 2021.

Location	2021 ASR						1990–2021 EAPC					
	NDs	NPB	NE	NS	HD	Other neonatal disorders	NDs	NPB	NE	NS	HD	Other neonatal disorders
Global	2941.98 (2547.76 to 3384.2)	1254.24 (1088.18 to 1465.54)	932.14 (796.29 to 1101.54)	370.55 (315.48 to 425.82)	58.33 (46.55 to 71.69)	326.72 (236.53 to 394.4)	−1.31 (−1.38 to −1.24)	−1.40 (−1.44 to −1.36)	−1.11 (−1.24 to −0.99)	−0.79 (−0.91 to −0.67)	−3.06 (−3.16 to −2.96)	−1.61 (−1.75 to −1.48)
Four World Regions												
Africa	4194.32 (3531.46 to 4928.36)	1518.78 (1293.2 to 1785.03)	1525.94 (1267.97 to 1828.71)	637.91 (527.88 to 760.43)	48.03 (38.55 to 64.63)	463.66 (275.18 to 609.21)	−1.03 (−1.10 to −0.95)	−0.94 (−1.01 to −0.86)	−0.80 (−0.93 to −0.67)	−0.96 (−1.02 to −0.90)	−3.48 (−3.60 to −3.35)	−1.69 (−1.79 to −1.58)
America	1221.54 (1032.57 to 1418.29)	598.32 (510.78 to 698.07)	262.25 (221.44 to 310.83)	211.38 (170.73 to 252)	16.41 (13.24 to 19.81)	133.18 (110.86 to 159.59)	−2.26 (−2.32 to −2.21)	−2.76 (−2.82 to −2.70)	−2.28 (−2.34 to −2.23)	−1.48 (−1.72 to −1.25)	−2.68 (−2.83 to −2.53)	−0.58 (−0.76 to −0.41)
Asia	2761.2 (2394.86 to 3190.57)	1319.1 (1115.05 to 1558.89)	785.11 (643.21 to 968.48)	262.13 (219.78 to 306.19)	81.7 (63.28 to 101.51)	313.16 (245.81 to 396.72)	−1.73 (−1.83 to −1.63)	−1.55 (−1.62 to −1.48)	−1.86 (−2.02 to −1.70)	−1.42 (−1.57 to −1.27)	−2.79 (−2.91 to −2.66)	−2.07 (−2.23 to −1.90)
Europe	627.95 (554.93 to 707.37)	353.93 (311.61 to 397.46)	116.98 (102.1 to 132.86)	98.89 (80.04 to 119.42)	9.22 (7.51 to 11.18)	48.94 (40.4 to 58.4)	−3.40 (−3.46 to −3.34)	−3.61 (−3.69 to −3.54)	−3.75 (−3.81 to −3.68)	−1.60 (−1.71 to −1.49)	−4.59 (−4.69 to −4.49)	−3.43 (−3.59 to −3.26)
SDI												
High SDl	536.18 (477.4 to 596.57)	334.94 (296.63 to 374.55)	99.37 (86.55 to 112.88)	48.74 (40.18 to 58.33)	3.05 (2.4 to 3.81)	50.07 (45.03 to 55.28)	−1.87 (−1.95 to −1.78)	−1.96 (−2.06 to −1.86)	−1.73 (−1.82 to −1.63)	−2.01 (−2.13 to −1.89)	−2.35 (−2.73 to −1.97)	−1.29 (−1.45 to −1.13)
High-middle SDl	708.68 (630.99 to 789.95)	383.41 (342.02 to 431.67)	157.82 (136.66 to 182.89)	97.21 (81.83 to 113.53)	12.38 (10.2 to 14.8)	57.87 (46.85 to 69.03)	−3.85 (−3.99 to −3.70)	−3.64 (−3.77 to −3.51)	−4.92 (−5.12 to −4.71)	−1.87 (−2.01 to −1.72)	−5.08 (−5.21 to −4.96)	−3.84 (−4.07 to −3.61)
Middle SDl	1732.97 (1503.6 to 1990.8)	887.08 (763.42 to 1022.9)	429.93 (369.87 to 501.71)	226.07 (191.58 to 265.13)	31.3 (26.24 to 37.36)	158.59 (130.77 to 200.73)	−2.27 (−2.38 to −2.17)	−2.02 (−2.11 to −1.93)	−3.06 (−3.22 to −2.91)	−0.98 (−1.21 to −0.76)	−3.98 (−4.07 to −3.90)	−2.25 (−2.36 to −2.14)
Low-middle SDl	3760.88 (3254.69 to 4308.21)	1718.94 (1449.88 to 2019.09)	1130.55 (942.77 to 1350.76)	401.64 (333.05 to 478.45)	93.29 (71.43 to 116.94)	416.45 (320.99 to 522.1)	−1.53 (−1.58 to −1.48)	−1.48 (−1.54 to −1.42)	−1.18 (−1.31 to −1.04)	−1.40 (−1.47 to −1.33)	−3.16 (−3.25 to −3.06)	−2.19 (−2.30 to −2.08)
Low SDI	4600.85 (3875.27 to 5441.74)	1667.11 (1421.01 to 1982.42)	1653.75 (1388.53 to 1994.4)	652.78 (530.62 to 799.59)	78.77 (61.87 to 110.59)	548.44 (325.7 to 733.8)	−1.01 (−1.08 to −0.94)	−0.96 (−1.04 to −0.88)	−0.71 (−0.81 to −0.60)	−0.99 (−1.03 to −0.95)	−2.93 (−3.05 to −2.80)	−1.59 (−1.67 to −1.50)
Regions												
Andean Latin America	1333.83 (1091.04 to 1597.89)	557.68 (453.33 to 672.95)	354.45 (277.35 to 446.61)	327.34 (245.36 to 427.77)	24.33 (19.06 to 30.18)	70.03 (54.15 to 89.93)	−2.70 (−2.83 to −2.56)	−3.24 (−3.42 to −3.07)	−2.60 (−2.76 to −2.44)	−1.64 (−1.86 to −1.42)	−2.62 (−2.69 to −2.55)	−3.08 (−3.41 to −2.75)
Australasia	449.81 (386.06 to 521.27)	273.81 (232.18 to 322.08)	95.88 (82.39 to 112.28)	34.54 (26.8 to 44.26)	0.91 (0.69 to 1.19)	44.66 (36.92 to 53.46)	−1.48 (−1.61 to −1.34)	−1.70 (−2.05 to −1.36)	−0.92 (−1.39 to −0.44)	−1.82 (−2.11 to −1.52)	−4.74 (−6.07 to −3.40)	0.04 (−1.36 to 1.45)
Caribbean	2774.12 (2308.21 to 3312.21)	1124.83 (900.5 to 1401.62)	793.47 (620.55 to 1028.04)	547.81 (414.15 to 707.38)	35.87 (27.33 to 48.25)	272.14 (190.89 to 376.48)	−0.52 (−0.62 to −0.43)	−1.14 (−1.25 to −1.03)	−0.21 (−0.33 to −0.09)	0.40 (0.26 to 0.53)	−1.97 (−2.08 to −1.86)	0.01 (−0.14 to 0.17)
Central Asia	1636.93 (1425.24 to 1882.14)	723.84 (618.02 to 840.05)	489.1 (417.29 to 566.43)	165.34 (137.94 to 194.78)	28.61 (23.11 to 34.98)	230.04 (189.1 to 279.61)	−1.18 (−1.53 to −0.83)	−0.56 (−0.81 to −0.32)	−2.67 (−3.26 to −2.08)	0.66 (0.35 to 0.96)	−1.94 (−2.23 to −1.65)	0.71 (0.38 to 1.04)
Central Europe	599.19 (518.26 to 680.62)	361.59 (315.07 to 411.38)	101.37 (85.1 to 118.63)	82.73 (58.04 to 113.01)	10.03 (7.84 to 12.69)	43.46 (35.51 to 52.17)	−3.77 (−3.92 to −3.62)	−3.75 (−3.94 to −3.56)	−4.31 (−4.52 to −4.11)	−2.23 (−2.50 to −1.97)	−3.46 (−3.60 to −3.32)	−4.92 (−5.33 to −4.51)
Central Latin America	1378.88 (1128.54 to 1672.05)	660.29 (541.61 to 804.47)	301.48 (250.38 to 362.38)	293.42 (233.78 to 362.05)	23.43 (18.67 to 28.45)	100.27 (78.65 to 128.36)	−2.30 (−2.40 to −2.21)	−2.70 (−2.83 to −2.57)	−2.77 (−2.97 to −2.58)	−0.55 (−0.72 to −0.39)	−2.59 (−2.82 to −2.35)	−1.78 (−1.91 to −1.65)
Central Sub-Saharan Africa	3596.56 (2845.36 to 4300.16)	1028.86 (818.16 to 1290.82)	1644.68 (1309.19 to 2071.11)	341.91 (209.93 to 537.69)	25.43 (17.13 to 42.3)	555.68 (225.1 to 989.28)	−0.50 (−0.74 to −0.26)	−0.54 (−0.75 to −0.33)	−0.59 (−0.83 to −0.34)	0.43 (0.14 to 0.72)	−1.46 (−1.74 to −1.19)	−0.62 (−0.92 to −0.32)
East Asia	627.2 (548.39 to 722.62)	305.77 (263.36 to 356.47)	232.3 (198.69 to 270.84)	37.09 (29.41 to 46.27)	13.35 (10.85 to 16.17)	38.69 (30.65 to 48.89)	−4.80 (−5.11 to −4.49)	−4.11 (−4.39 to −3.82)	−5.64 (−6.06 to −5.22)	−2.51 (−2.63 to −2.39)	−6.51 (−6.69 to −6.32)	−4.89 (−5.17 to −4.62)
Eastern Europe	593.47 (528.71 to 662.63)	279.1 (253 to 309.06)	118.52 (103.71 to 133.58)	158.75 (132.62 to 186.17)	16.63 (13.09 to 20.48)	20.48 (18.25 to 22.84)	−3.46 (−3.67 to −3.25)	−3.47 (−3.74 to −3.20)	−5.37 (−5.59 to −5.15)	−0.65 (−0.95 to −0.35)	−2.93 (−3.00 to −2.87)	−2.52 (−2.83 to −2.21)
Eastern Sub-Saharan Africa	4139.25 (3368.02 to 5046.89)	1193.44 (981.08 to 1464.23)	1469.06 (1188.42 to 1800)	768.92 (608.87 to 976.27)	58.16 (42.86 to 89.65)	649.68 (348.85 to 927.76)	−1.26 (−1.35 to −1.18)	−1.16 (−1.22 to −1.10)	−1.11 (−1.27 to −0.95)	−1.29 (−1.36 to −1.23)	−2.42 (−2.52 to −2.31)	−1.61 (−1.71 to −1.51)
High-income Asia Pacific	268.83 (225.02 to 313.87)	143.32 (117.9 to 167.25)	74.03 (59.16 to 90.02)	26.33 (20.74 to 32.91)	2.13 (1.69 to 2.65)	23.01 (20.01 to 25.89)	−2.48 (−2.63 to −2.32)	−2.66 (−2.83 to −2.49)	−1.74 (−1.98 to −1.50)	−2.78 (−2.87 to −2.69)	−1.67 (−2.12 to −1.21)	−2.90 (−3.06 to −2.75)
High-income North America	680.35 (604.95 to 759.01)	450.21 (398.6 to 502.58)	106.05 (93.89 to 118.36)	44.51 (38.95 to 50.45)	2.55 (1.96 to 3.19)	77.02 (68.84 to 85.87)	−0.96 (−1.10 to −0.83)	−1.04 (−1.18 to −0.91)	−0.71 (−0.80 to −0.61)	−0.67 (−0.93 to −0.41)	−1.18 (−1.45 to −0.91)	−0.97 (−1.23 to −0.70)
North Africa and Middle East	1606.87 (1399.35 to 1831.89)	977.35 (841.33 to 1135.46)	299.98 (247.42 to 364.85)	116.68 (92.17 to 142.41)	37.16 (28.93 to 49.71)	175.71 (110.41 to 233.66)	−3.27 (−3.39 to −3.15)	−3.48 (−3.64 to −3.32)	−2.46 (−2.58 to −2.33)	−2.09 (−2.21 to −1.97)	−4.06 (−4.15 to −3.98)	−3.61 (−3.72 to −3.49)
Oceania	2211.09 (1803.34 to 2652.71)	1103.83 (870.46 to 1366.46)	474.61 (343.67 to 635.39)	174.32 (116.56 to 252.59)	10.2 (7.29 to 15.51)	448.13 (309.79 to 641.46)	−0.33 (−0.42 to −0.25)	−0.18 (−0.32 to −0.04)	−0.51 (−0.65 to −0.37)	0.02 (−0.22 to 0.26)	−1.53 (−1.63 to −1.43)	−0.58 (−0.68 to −0.47)
South Asia	4276.74 (3675.53 to 4994.6)	2064.65 (1717.27 to 2473.36)	1205.88 (958.28 to 1561.67)	355.92 (292.81 to 427.86)	141.24 (107.01 to 179.14)	509.05 (387.42 to 651.02)	−1.38 (−1.42 to −1.33)	−1.19 (−1.29 to −1.09)	−1.16 (−1.27 to −1.06)	−1.47 (−1.55 to −1.38)	−2.48 (−2.63 to −2.32)	−2.09 (−2.17 to −2.02)
Southeast Asia	1852.5 (1576.86 to 2125.1)	851.59 (719.82 to 993.2)	468.19 (352.69 to 569.83)	358.57 (281.21 to 468.66)	30.39 (25.23 to 36.53)	143.75 (100.37 to 245.17)	−1.97 (−2.04 to −1.89)	−2.00 (−2.04 to −1.96)	−1.91 (−2.04 to −1.78)	−1.75 (−1.90 to −1.60)	−4.00 (−4.06 to −3.94)	−1.87 (−1.91 to −1.83)
Southern Latin America	943.88 (785.7 to 1113.08)	584.99 (487.46 to 694.16)	145.51 (120.34 to 172.83)	125.92 (102.89 to 150.95)	9.14 (6.93 to 11.65)	78.32 (62.16 to 98.9)	−2.75 (−2.87 to −2.63)	−2.69 (−2.81 to −2.57)	−3.22 (−3.47 to −2.98)	−2.95 (−3.17 to −2.73)	−3.27 (−3.53 to −3.01)	−1.68 (−1.84 to −1.52)
Southern Sub-Saharan Africa	3582.8 (3031.07 to 4285.94)	1523.43 (1255.22 to 1858.51)	807.61 (639.75 to 1034.42)	460.45 (372.47 to 557.75)	38.88 (30.84 to 47.88)	752.43 (580.25 to 979.61)	−0.52 (−0.62 to −0.42)	−0.19 (−0.29 to −0.10)	−0.97 (−1.10 to −0.84)	0.64 (0.50 to 0.78)	−2.30 (−2.45 to −2.16)	−1.10 (−1.18 to −1.02)
Tropical Latin America	1362.42 (1129.46 to 1622.38)	599.13 (501.58 to 709.91)	280.69 (233 to 336.31)	219.63 (179.81 to 266.33)	22.18 (17.86 to 26.86)	240.79 (196.14 to 299.82)	−3.06 (−3.24 to −2.88)	−4.12 (−4.18 to −4.07)	−2.64 (−2.89 to −2.40)	−2.96 (−3.46 to −2.45)	−3.01 (−3.11 to −2.91)	0.71 (0.21 to 1.21)
Western Europe	460.23 (405.44 to 519.07)	272.73 (236.48 to 308.19)	93.3 (81.98 to 106.2)	52.7 (40.57 to 66.46)	2.06 (1.65 to 2.57)	39.43 (33.82 to 45.25)	−1.63 (−1.76 to -1.50)	−1.96 (−2.11 to −1.82)	−1.54 (−1.71 to −1.37)	−0.74 (−0.86 to −0.63)	−3.25 (−3.73 to −2.77)	−0.15 (−0.40 to 0.10)
Western Sub-Saharan Africa	5286.87 (4504.47 to 6147.67)	2031.27 (1702.32 to 2416.97)	2034.92 (1661.59 to 2425.74)	800.47 (666.87 to 948.98)	50.32 (40.04 to 65.68)	369.89 (256.71 to 466.76)	−0.83 (−0.89 to −0.76)	−0.23 (−0.33 to −0.13)	−0.89 (−1.00 to −0.79)	−1.17 (−1.23 to −1.12)	−4.62 (−4.88 to −4.36)	−1.67 (−1.75 to −1.59)

### 3.3 Regional levels

#### 3.3.1 Incidence and prevalence

The ASIR and ASPR of NDs in South Asia for the year 2021 were 645.70 (636.10–656.13) and 3521.06 (3142.48–3910.85), respectively, establishing it as the region with the highest ASIR and ASPR of NDs among the 21 regions analyzed (Tables 2, 3). Between 1990 and 2021, a total of 11 regions exhibited a declining trend in NDs ASIR, with Southern Latin America demonstrating the most significant increase in NDs ASIR (EAPC = 0.49, 0.45–0.54), while East Asia recorded the largest decrease in NDs ASIR (EAPC = −1.40, −1.51 to −1.29; Table 2; Figure 1A). Nineteen regions displayed an increasing trend in NDs ASPR, with Tropical Latin America experiencing the most pronounced increase (EAPC = 1.26, 1.17–1.36), whereas Australia reported the largest decrease (EAPC = −0.18, −0.22 to −0.14; Table 3; Figure 2A). In 2021, the ASIR of NPB in South Asia was recorded at 543.78 (535.11–553.21), and the ASPR of NPB was 3113.16 (2715.63–3514.86), marking it as the region with the highest ASIR and ASPR of NPB among the 21 regions (Tables 2, 3). The highest ASIR rates for NE, NS, and HD in Eastern Sub-Saharan Africa, Southern Sub-Saharan Africa, and Andean Latin America were 34.57 (33.84–35.28), 152.66 (142.08–164.34), and 16.91 (12.92–21.95), respectively. In 2021, the highest ASPR for NE in Central Latin America, and NS in Southern Sub-Saharan Africa, and HD in Central Asia were 352.59 (309.56–397.76), 279.44 (214.23–346.37), and 49.62 (45.27–54.38), respectively (Tables 2, 3). From 1990 to 2021, over 50% of the regions exhibited a declining trend in NS and HD ASIR, while more than 50% of the regions showed an upward trend in NPB ASIR, and 20 regions reported a downward trend in NE ASIR (Table 2; Figures 1B–E). Furthermore, from 1990 to 2021, the ASPR of NPB, NE, NS, and HD exhibited an upward trend in over 50% of the regions (Table 3; Figures 2B–E).

#### 3.3.2 Deaths

In 2021, Western Sub-Saharan Africa exhibited the highest NDs ASDR, recorded at 56.74 (48.04–66.23) among the 21 regions (Table 4). Between 1990 and 2021, the NDs ASDR across all 21 regions demonstrated a downward trajectory, with the most significant reduction noted in East Asia (EAPC = −5.53, −5.92 to −5.15; Table 4; Figure 3A). In 2021, the ASDR in Western Sub-Saharan Africa for NPB, NE, and NS were the highest, at 21.57 (17.90–25.89), 22.11 (17.98–26.42), and 8.55 (7.07–10.13), respectively. Additionally, in 2021, the highest ASDR for HD in South Asia and other neonatal disorders in Southern Sub-Saharan Africa were recorded at 1.44 (1.06–1.87) and 8.31 (6.40–10.84), respectively (Table 4). From 1990 to 2021, the ASDR for NPB, NE, and HD across the 21 regions consistently declined, with over 50% of the regions showing a decreasing trend in the ASDR for NS and other neonatal disorders (Table 4; Figures 3B–E).

#### 3.3.3 DALYs

In 2021, among the 21 regions, Western Sub-Saharan Africa exhibited the highest age-standardized DALYs rate for NDs at 5286.87 (4504.47 to 6147.67; Table 5). From 1990 to 2021, the age-standardized DALYs rate for NDs demonstrated a declining trend across all 21 regions, with the most significant decrease observed in East Asia (EAPC = −4.80, −5.11 to −4.49; Table 5; Figure 4A). In 2021, the age-standardized DALYs rates for NE and NS in Western Sub-Saharan Africa were the highest, recorded at 2034.92 (1661.59 to 2425.74) and 800.47 (666.87 to 948.98), respectively. Furthermore, South Asia reported the highest age-standardized DALY rates for NPB, HD, and other neonatal disorders in Southern Sub-Saharan Africa in 2021, with rates of 2064.65 (1717.27 to 2473.36), 141.24 (107.01 to 179.14), and 752.43 (580.25 to 979.61), respectively (Table 5). Between 1990 and 2021, the age-standardized DALY rates for NPB, NE, and HD exhibited a downward trend in all 21 regions, while the age-standardized DALY rates for NS and other neonatal disorders showed a declining trend in over 50% of the regions (Table 5; Figures 4B–E).

### 3.4 National levels

#### 3.4.1 Incidence and prevalence

At the national level, in 2021, the Islamic Republic of Mauritania recorded the highest NDs ASIR and ASPR, at 753.99 (707.29 to 803.14) and 3818.88 (3132.76 to 4854.34), respectively. From 1990 to 2021, over 50% of countries and regions experienced a downward trend in NDs ASIR, with Nepal showing the largest decline (EAPC = −1.62, −1.70 to −1.53). Conversely, more than 50% of countries and regions observed an upward trend in NDs ASPR, with Greece experiencing the largest increase (EAPC = 3.34, 2.94 to 3.73; Figures 1A, 2A). In 2021, the Republic of Mauritania had the highest NPB ASIR, at 644.60 (599.31 to 692.39). Additionally, Romania had the highest NS ASIR and ASPR in 2021, at 201.50 (181.60 to 222.54) and 545.99 (419.66 to 681.53), respectively. The Republic of Albania had the highest HD ASIR and ASPR in 2021, at 30.74 (24.05 to 38.46) and 93.02 (84.97 to 101.45), respectively. Furthermore, the Federal Republic of Somalia recorded the highest NE ASIR in 2021, at 56.13 (52.71 to 59.44). In the same year, India had the highest NPB, while Thailand had the highest NE ASPR, with rates of 3352.59 (2907.12 to 3788.88) and 517.00 (452.87 to 590.17), respectively. The Central African Republic had the highest NE ASIR in 2021, while Australia recorded the highest HD ASPR, with rates of 75.43 (56.55 to 98.69) and 1.50 (1.32 to 1.74), respectively. From 1990 to 2021, 87 countries and regions exhibited an increasing trend in NPB ASIR, while over 50% of countries and regions demonstrated a decreasing trend in NE, NS, and HD ASIR (Figures 1B–E). Over the same period, more than 50% of countries and regions showed an upward trend in NPB, NE, and NS ASPR, while over 50% exhibited a downward trend in HD ASPR (Figures 2B–E).

#### 3.4.2 Deaths

In 2021, the Republic of Mali recorded the highest NDs ASDR at 76.58 (64.57 to 90.59). From 1990 to 2021, more than 50% of countries and regions exhibited a downward trend in NDs ASDR, with Estonia demonstrating the most significant decline (EAPC = −8.20, −8.47 to −7.92; Figure 3A). Additionally, in 2021, the Republic of Mali also reported the highest NPB ASDR at 39.46 (31.54 to 48.43). Pakistan had the highest ASDR for NE and HD in 2021, with rates of 32.21 (24.64 to 40.19) and 2.91 (1.97 to 4.12), respectively. Furthermore, Chad and the Kingdom of Lesotho recorded the highest ASDR for NS and other neonatal disorders, at 10.86 (7.52 to 16.07) and 10.95 (7.08 to 15.74), respectively. From 1990 to 2021, over 50% of countries and regions experienced a declining trend in ASDR for NPB, NE, NS, HD, and other neonatal disorders (Figures 3B–E).

#### 3.4.3 DALYs

In 2021, the Republic of Mali recorded the highest age-standardized DALYs rate for NDs at 7062.76 (5972.89 to 8338.85). From 1990 to 2021, over 50% of countries and regions experienced a decline in the age-standardized DALYs rate for NDs, with Saudi Arabia exhibiting the most significant decrease (EAPC = −6.39, −6.56 to −6.23; Figure 4A). Additionally, in 2021, the Republic of Mali had the highest age-standardized DALYs rate for NPB at 3640.04 (2940.35 to 4462.03). Furthermore, Pakistan reported the highest age-standardized DALYs rates for NE and HD in 2021, with rates of 2927.96 (2245.01 to 3644.70) and 281.29 (197.85 to 390.05), respectively. Chad and the Kingdom of Lesotho recorded the highest ASDR for NS and other neonatal disorders, at 1001.41 (699.33 to 1464.48) and 989.33 (641.52 to 1419.80), respectively. From 1990 to 2021, the age-standardized DALYs rates for NPB, NE, NS, HD, and other neonatal disorders exhibited a downward trend in more than 50% of countries and regions (Figures 4B–E).

## 4 SDI and neonatal burden of disease

In 2021, the ASIR of NDs, the ASDR of NDs, the age-standardized DALYs rate in low SDI countries, and the ASPR of NDs in low-middle SDI countries were the highest, recorded at 516.75 (509.51 to 524.01), 48.03 (39.93 to 57.41), 4600.85 (3875.27 to 5441.74), and 2643.47 (2373.26 to 2946.65), respectively (Tables 2–5). From 1990 to 2021, the ASIR of NDs exhibited an increasing trend only in high SDI countries, while a decreasing trend was observed in the other four categories of SDI countries. Conversely, the ASPR of NDs across all five SDI categories demonstrated an upward trend, whereas both the ASDR and age-standardized DALYs rate of NDs showed a declining trend (Table 2–5; Figures 1–4A).

The ASIR of NE, NS, and HD were highest in low SDI countries in 2021. In contrast, the NPB ASIR was highest in low-middle SDI countries, with respective values of 25.84 (25.47 to 26.22), 79.67 (77.16 to 82.49), 12.32 (9.50 to 16.09), and 416.03 (409.36 to 423.17; Table 2). Furthermore, in 2021, the NPB, NS, and HD ASPR were highest in low-middle SDI countries, while the NE ASPR was highest in high-middle SDI countries, with values of 2270.83 (1969.31–2593.52), 151.79 (117.61–188.01), 35.88 (32.97–39.43), and 276.83 (243.01–310.24), respectively (Table 3). From 1990 to 2021, the NPB ASIR exhibited an upward trend in high SDI countries (EAPC = 0.23, 0.16 to 0.31), whereas a downward trend was observed in the NPB ASIR across the other four SDI categories. Additionally, the NE and NS ASIR across all five SDI categories showed a downward trend. The HD ASIR in high, high-middle, middle, and low-middle SDI countries also exhibited a downward trend (Table 2; Figures 1B–E). Between 1990 and 2021, the NPB ASPR in low SDI countries (EAPC = −0.14, −0.17 to −0.11) and the NS ASPR in high-middle SDI countries (EAPC = −0.43, −0.60 to −0.25) demonstrated a declining trend (Table 3; Figures 2B,D).

In 2021, low-SDI countries had the highest ASDR for NPB, NE, NS, and other neonatal disorders, while low-middle SDI countries had the highest ASDR for HD, with rates of 16.62 (13.66 to 19.98), 17.77 (14.77 to 21.58), 6.85 (5.52 to 8.54), 6.05 (3.57 to 8.12), and 0.90 (0.67 to 1.17), respectively (Table 4). In 2021, low SDI countries exhibited the highest age-standardized DALYs rates for NE, NS, and other neonatal disorders, while lower-middle SDI countries showed the highest age-standardized DALYs rates for NPB and HD, which were 1653.75 (1388.53–1994.40), 652.78 (530.62–799.59), 548.44 (325.70–733.80), 1718.94 (1449.88–2019.09), and 93.29 (71.43–116.94), respectively (Table 5). From 1990 to 2021, the ASDR and age-standardized DALYs rates for NPB, NE, NS, HD, and other neonatal disorders all showed a declining trend across five different SDI countries (Tables 4–5; Figures 3, 4B–F).

## 5 Gender specific differences in the global burden of neonatal disorders

**Figure 5:**
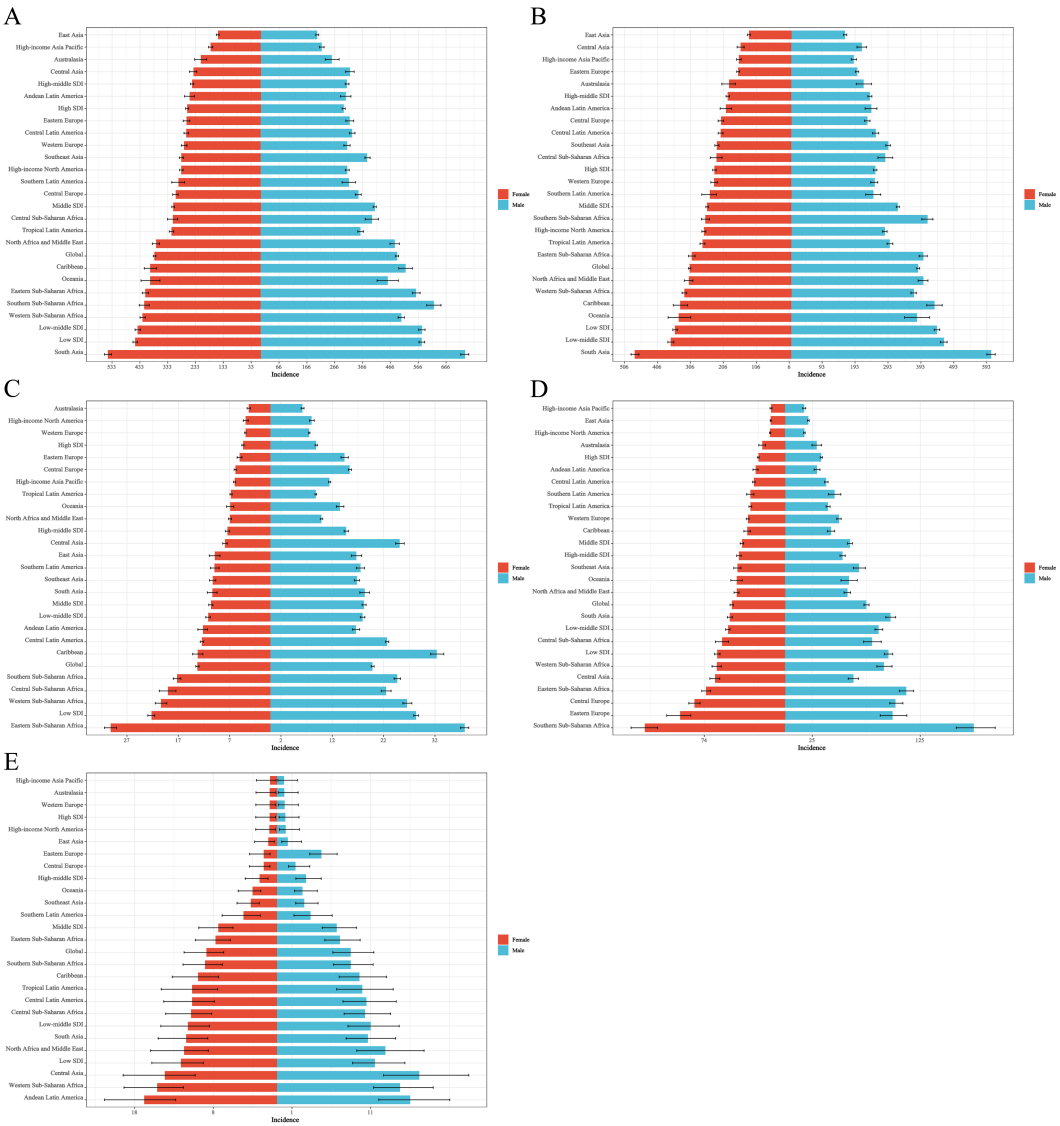
Global ASIR of neonatal disorders by gender and etiology of four neonatal disorders in 2021: **(A)** NDs, **(B)** NPB, **(C)** NE, **(D)** NS, **(E)** HD.

**Figure 6:**
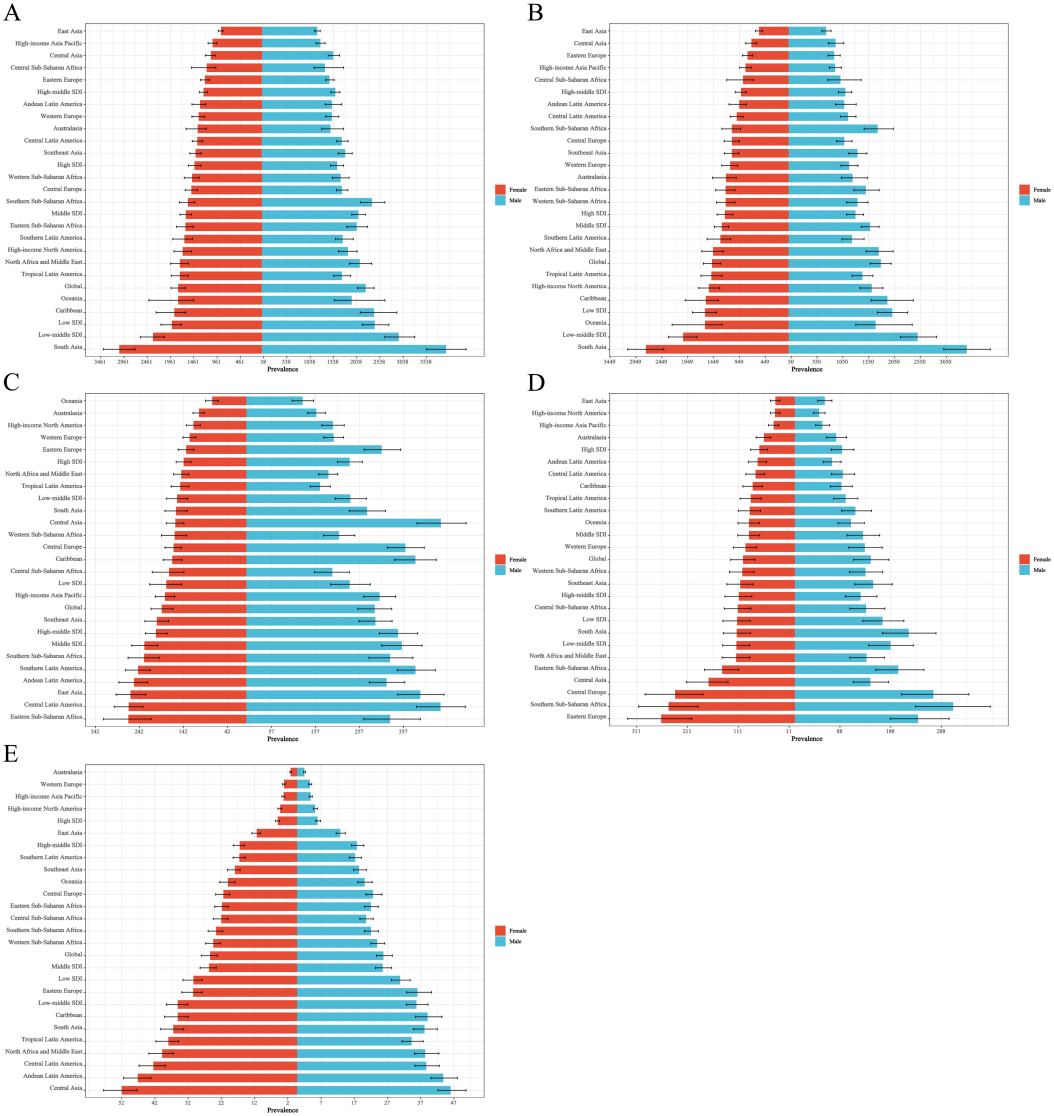
Global ASPR of neonatal disorders by gender and etiology of four neonatal disorders in 2021: **(A)** NDs, **(B)** NPB, **(C)** NE, **(D)** NS, **(E)** HD.

**Figure 7:**
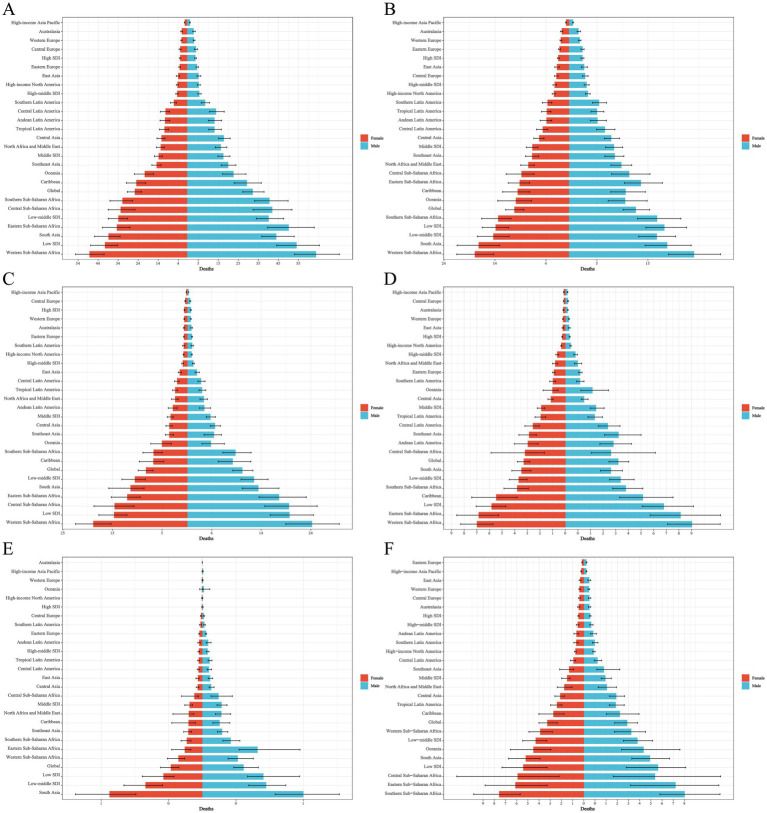
Global ASDR of neonatal disorders by sex and etiology of five neonatal disorders in 2021: **(A)** NDs, **(B)** NPB, **(C)** NE, **(D)** NS, **(E)** HD, **(F)** Other neonatal disorders.

**Figure 8:**
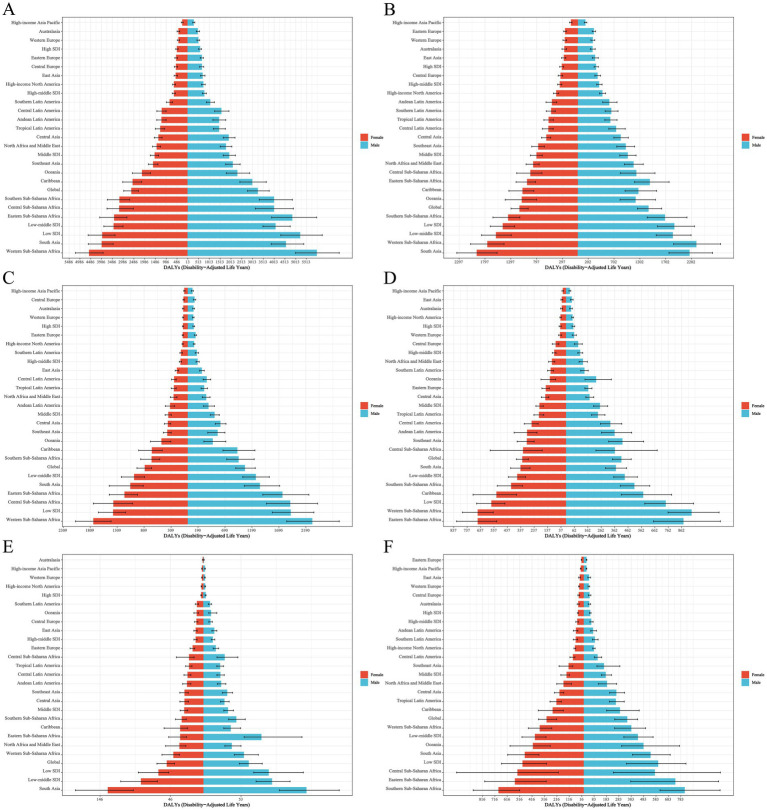
Global age-standardized DALYs rates of neonatal disorders by sex and etiology of five neonatal disorders in 2021: **(A)** NDs, **(B)** NPB, **(C)** NE, **(D)** NS, **(E)** HD, **(F)** Other neonatal disorders.

The global ASIR, ASPR, ASDR, and age-standardized DALYs rate for males in 2021 were 489.90 (484.15 to 495.69), 2235.04 (2056.50 to 2411.83), 32.81 (27.80 to 38.40), and 3258.94 (2780.65 to 3785.91), respectively. For females, these rates were 381.24 (376.42 to 385.90), 1794.20 (1644.92 to 1949.24), 26.10 (22.72 to 29.83), and 2603.09 (2274.86 to 2954.93), respectively (Figures 5–8A). In 2021, with the exception of the global male HD ASPR, which was slightly lower than that of females, the global male NPB, NE, NS, and other neonatal disorders burdens were all higher than those of females (Figures 6B–F). Additionally, in terms of ASIR, ASPR, ASDR, and age-standardized DALYs rates, the global NPB ASIR, ASPR, NE ASDR, and age-standardized DALYs rates exhibited the largest gender disparities, with male-to-female ratios of 385.35: 308.84, 1780.47: 1481.22, 11.07: 8.34, and 1060.17: 795.36, respectively (Figures 5–8B,C).

In 2021, among the four world regions, Asia exhibited the most pronounced gender disparities in NDs, NPB, NS ASIR, and ASPR, with the most significant gender difference in NE ASPR observed in Asia. In contrast, the HD ASPR for males in Africa and the Americas was slightly lower than that for females, with male-to-female ratios of 27.42: 28.37 and 25.92: 28.02, respectively. Notably, the Americas displayed the largest gender difference in NE ASIR, with a male-to-female ratio of 14.89: 9.38. Furthermore, Europe presented the greatest gender disparities in HD ASIR and ASPR in 2021, with ratios of 2.95: 1.91 and 16.52: 15.43, respectively. Among the four world regions, Africa demonstrated the largest gender disparities in ASDR for NDs, NPB, NE, NS, HD, and other neonatal disorders, with male-to-female ratios of 50.57: 37.36, 17.92: 13.25, 18.90: 13.60, 7.64: 5.60, 0.54: 0.27, and 5.56: 4.64, respectively. Additionally, Africa exhibited the highest gender disparities in age-standardized DALYs rates for NDs, NPB, NE, NS, HD, and other neonatal disorders, with male-to-female ratios of 4788.74: 3573.19, 1727.86: 1300.17, 1763.70: 1277.46, 732.88: 538.93, 59.95: 35.57, and 504.36: 421.06, respectively.

In 2021, among the 21 regions analyzed, the NDs, NPB ASPR in Tropical Latin America, Central Europe, NPB ASPR in Southern Latin America, NS ASPR in Eastern Europe, NS ASIR in Central Asia, and ASPR exhibited a lower disease burden in males compared to females (Figures 5D, 6A,B,D). Additionally, in 2021, the HD ASPR in males was slightly lower than that in females across 11 regions, reflecting minor gender differences (Figure 6E). Notably, the NDs ASIR, ASPR, and NPB ASIR in Sub-Saharan Africa displayed the largest gender disparities, with male-to-female ratios of 622.20: 417.70, 2365.18: 1587.14, and 413.32: 260.59, respectively (Figures 5, 6A,B). Furthermore, South Asia demonstrated the most significant gender disparity in NPB ASPR, NS ASIR, and ASPR, with male-to-female ratios of 3449.40: 2758.56, 97.66: 51.09, and 223.54: 113.82, respectively (Figures 5, 6A,D). In the same year, Central Asia exhibited the greatest gender difference in HD ASPR, where the female HD ASPR surpassed the male HD ASPR, resulting in a male-to-female ratio of 46.23: 52.78. Eastern Europe had the largest gender disparity in HD ASIR, with a male-to-female ratio of 5.63: 1.68 (Figures 5, 6E). Additionally, in 2021, among the 21 regions, Oceania’s NE ASDR, the high-income Asia-Pacific region’s NE, and other neonatal disease ASDR indicated that males had lower rates than females (Figures 7C,F). Finally, the gender disparities in ASDR for NDs, NPB, and NS were most pronounced in Western sub-Saharan Africa, with male-to-female ratios of 64.42: 48.71, 24.53: 18.47, and 10.02: 7.00, respectively (Figures 7A,B,D). In 2021, the largest gender disparities in ASDR for NE, HD, and other neonatal disorders in Eastern Sub-Saharan Africa were observed, with male-to-female ratios of 18.51: 12.13, 0.82: 0.26, and 8.19: 6.12, respectively (Figures 7C,E,F). Among the 21 regions in 2021, Oceania exhibited a gender disparity in age-standardized DALYs rates for NE while the high-income Asia-Pacific region demonstrated a gender disparity in age-standardized DALYs rates for other neonatal disorders, where males exhibited lower rates than females (Figures 8C,F). Additionally, in 2021, the largest gender disparities in age-standardized DALYs rates for NDs, NPB, and NE in Eastern Sub-Saharan Africa were recorded, with male-to-female ratios of 4847.56: 3400.05, 1393.23: 984.81, and 1755.69: 1169.68, respectively (Figures 8A–C). In 2021, the gender disparity in age-standardized DALYs rates was most pronounced for NS in Eastern Sub-Saharan Africa, HD in Western Sub-Saharan Africa, and other neonatal disorders, with male-to-female ratios of 937.24: 657.86, 82.54: 32.72, and 740.86: 554.43, respectively (Figures 8D–F).

In 2021, both low SDI and middle SDI countries exhibited a lower disease burden in males compared to females, with the most significant gender disparity in the NE ASIR observed in middle SDI countries, where the male-to-female ratio was 18.26: 11.56 (Figures 6E, 5C). Additionally, the largest gender disparities in NDs, NPB, and NS ASIR and ASPR were noted in low SDI countries during the same year (Figures 5, 6A,B,D). In high-middle SDI countries, the most pronounced gender disparities in NE ASPR, HD ASIR, and ASPR were recorded, with male-to-female ratios of 344.73: 205.12, 3.67: 2.20, and 18.06: 17.27, respectively (Figures 5, 6C,E). Among the five categories of SDI countries in 2021, low SDI countries displayed the greatest gender disparities in ASDR for NDs, NPB, NE, NS, HD, and other neonatal disorders, with male-to-female ratios of 54.61: 41.08, 18.72: 14.40, 20.57: 14.81, 7.79: 5.85, 0.91: 0.58, and 6.63: 5.44, respectively (Figures 7A–F). Furthermore, within the five countries classified by different SDI levels, the low SDI country demonstrated the largest gender disparity in age-standardized DALYs rates for NDs, NPB, NE, NS, HD, and other neonatal disorders, with male-to-female ratios of 5219.67: 3949.38, 1843.57: 1586.40, 1910.85: 1382.75, 743.91: 557.05, 93.05: 63.71, and 600.91: 493.06, respectively (Figures 8A–F).

## 6 Cross country inequality analysis

**Figure 9:**
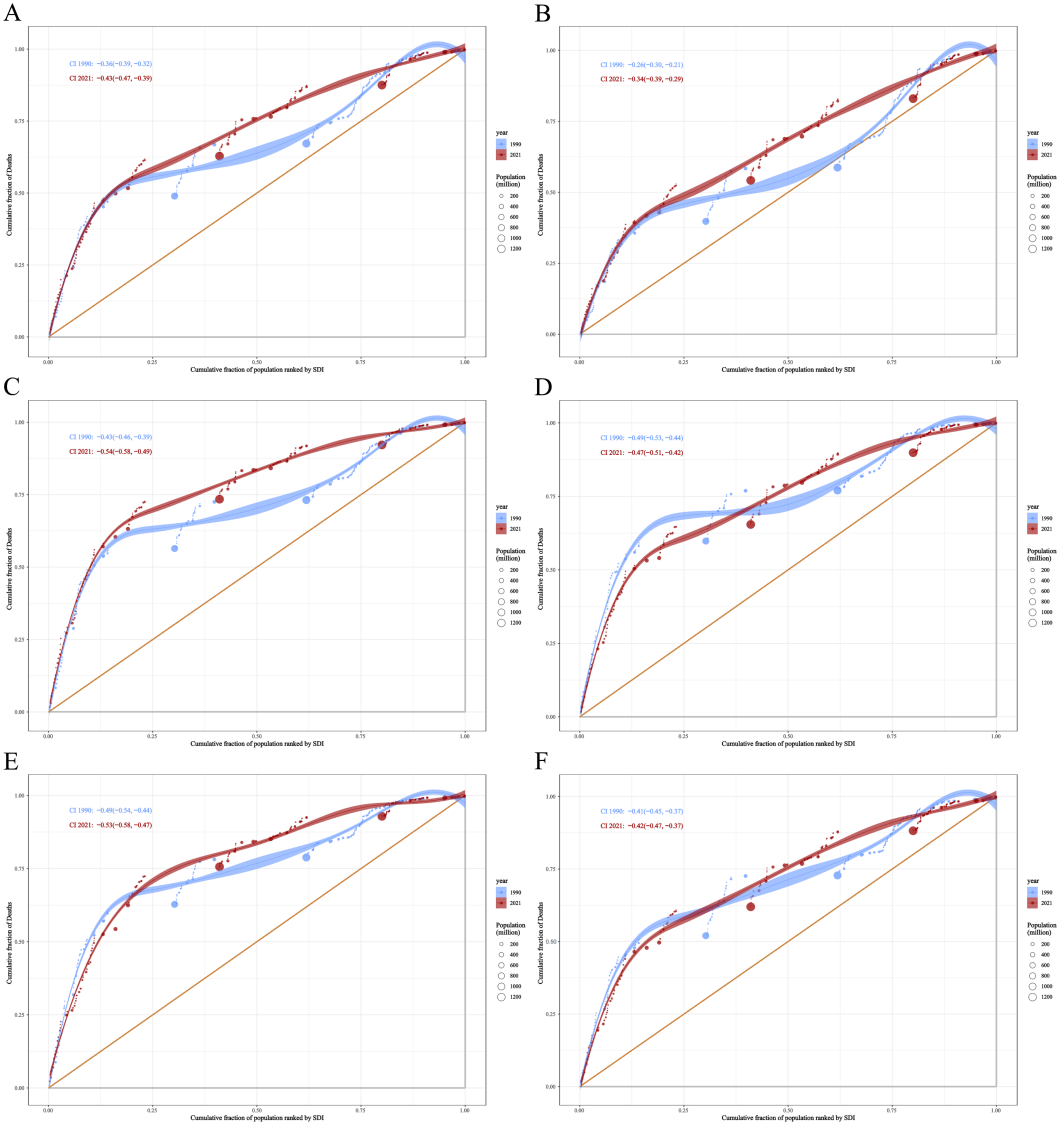
Concentration index of ASDR health inequalities for neonatal disorders and causes of five neonatal disorders **(A)** NDs **(B)** NPB, **(C)** NE, **(D)** NS, **(E)** HD, **(F)** Other neonatal disorders.

**Figure 10:**
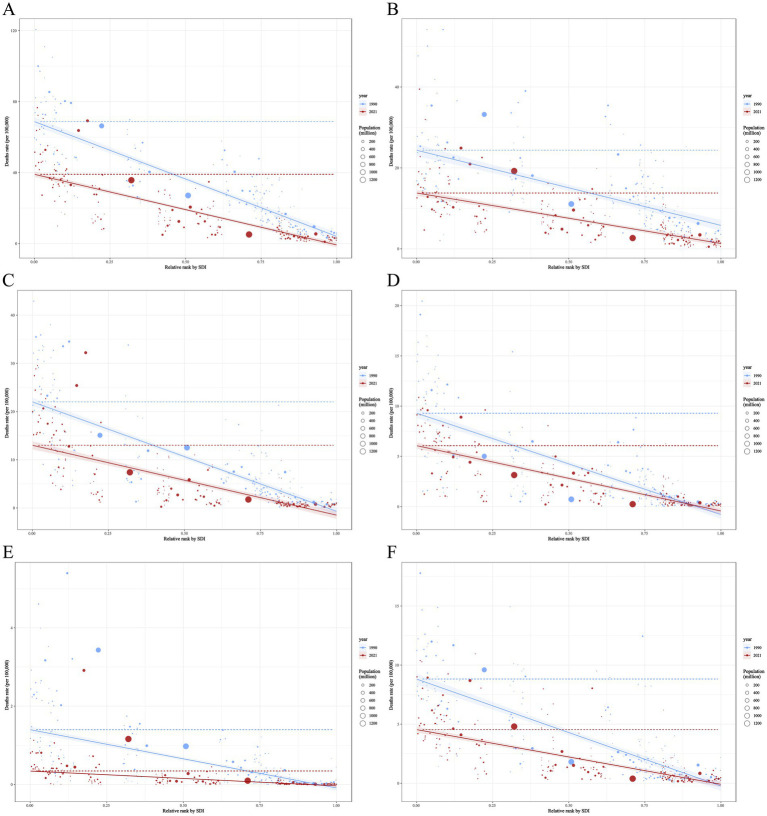
Slope index of inequality of ASDR health inequalities for neonatal disorders and causes of five neonatal disorders **(A)** NDs **(B)** NPB, **(C)** NE, **(D)** NS, **(E)** HD, **(F)** Other neonatal disorders.

This paper quantifies the disparities in the burden of NDs using data from the GBD 2021 database, employing the CI and the SII as analytical tools. From the perspective of ASDR, the CI for NDs decreased from −0.36 (95% CI = −0.39 to −0.32) in 1990 to −0.43 (−0.47 to −0.39) in 2021 (Figure 9A). Conversely, the SII for NDs increased from −64.72 (−69.57 to −59.88) in 1990 to −39.80 (−42.87 to −36.72) in 2021 (Figure 10A). This trend indicates that, between 1990 and 2021, the absolute inequality in the burden of NDs among countries with different SDI decreased, while relative inequality increased.

The ASDR CI for NPB, NE, HD, and other neonatal disorders decreased from −0.26 (−0.30 to −0.21), −0.43 (−0.46 to −0.39), −0.49 (−0.54 to −0.44), and −0.41 (−0.45 to −0.37) in 1990 to −0.34 (−0.39 to −0.29), −0.54 (−0.58 to −0.49), −0.53 (−0.58 to −0.47), and −0.42 (−0.47 to −0.37) in 2021, respectively (Figures 9A,B,E,F). The ASDR CI for NS increased from −0.49 (−0.53 to −0.44) in 1990 to −0.47 (−0.51 to −0.42) in 2021 (Figure 9D). The SII of ASDR for NPB, NE, NS, HD, and other neonatal disorders increased from −18.59 (−21.28 to −15.89), −22.72 (−24.67 to −20.77), −10.09 (−11.08 to −9.09), −1.49 (−1.68 to −1.30), and −9.05 (−9.93 to −8.16) in 1990 to −12.49 (−13.92 to −11.05), −14.51 (−16.06 to −12.95), −6.50 (−7.16 to −5.84), −0.38 (−0.42 to −0.34), and −4.63 (−5.13 to −4.13) in 2021, respectively (Figures 10B–F). This indicates that between 1990 and 2021, the absolute inequality in the burden of NPB, NE, HD, and other neonatal disorders among countries with a SDI decreased, while the absolute inequality in NS increased. Furthermore, relative inequality in NPB, NE, NS, HD, and other neonatal disorders among SDI countries also increased during this period.

## 7 Decomposition analysis

**Figure 11:**
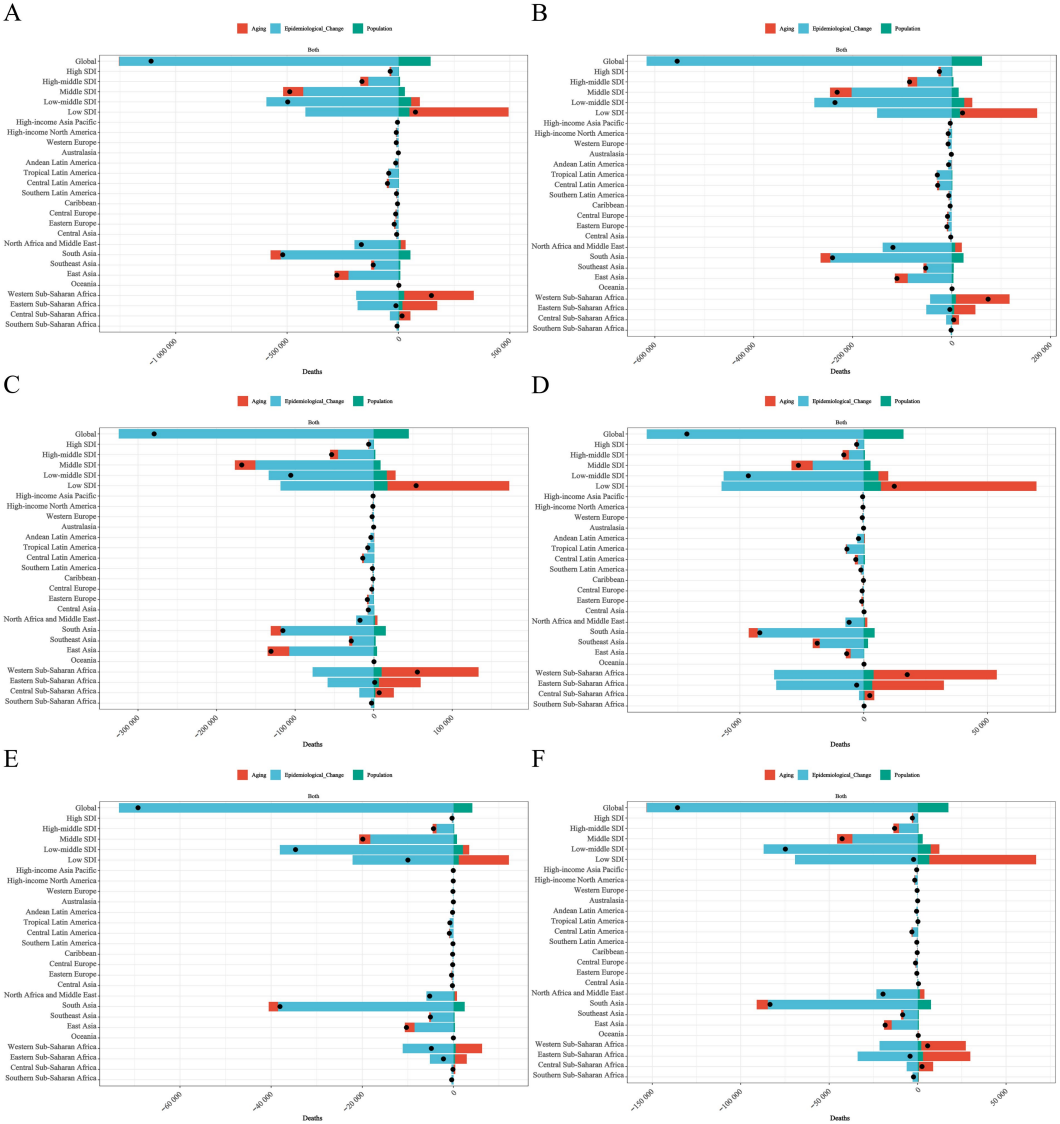
Changes in global neonatal disorders and ASDR of five causes of neonatal disorders due to population level determinants (including population aging, population growth, epidemiological changes) from 1990 to 2021 **(A)** NDs **(B)** NPB, **(C)** NE, **(D)** NS, **(E)** HD, **(F)** Other neonatal disorders.

This study utilizes decomposition analysis to assess the effects of population aging, population growth, and epidemiological changes on the ASDR of NDs globally, across 21 regions, and within five different SDI countries. At the global level, the total change in ASDR for NDs was −1,110,650.37, with population growth accounting for −12.96% and epidemiological changes for 112.96% (Figure 11A). The total ASDR change for NPB was −554,858.08, with population growth contributing −11.07% and epidemiological changes contributing 111.07% (Figure 11B). For NE, the total ASDR change was −279,476.44, with population growth contributing −16.02% and epidemiological changes contributing 116.02% (Figure 11C). The total ASDR change for NS was −71,389.04, with population aging contributing 0.01%, population growth contributing −22.60%, and epidemiological changes contributing 122.60% (Figure 11D). The total ASDR change for HD was −69,208.67, with population growth contributing −6.00% and epidemiological changes contributing 106.00% (Figure 11E). Lastly, the total ASDR change for other neonatal disorders was −135,718.16, with population growth contributing −12.88% and epidemiological changes contributing 112.88% (Figure 11F).

At the regional level across 21 areas, the total ASDR change for NDs in Western Sub-Saharan Africa was 147,980.45, representing the highest total ASDR change for NDs among all regions. Within this context, population aging contributed 211.02%, population growth contributed 17.31%, and epidemiological changes contributed −128.33% (Figure 11A). Compared to other regions, Western Sub-Saharan Africa also exhibited the highest total ASDR changes for NPB, NE, NS, and other neonatal disorders, with values of 73,899.93, 55,638.15, 17,615.20, and 5,668.89, respectively (Figures 11B,D,F). In contrast, Oceania’s total ASDR change was −5.16, marking the highest total ASDR change among HD, with population aging contributing −81.52%, population growth contributing −7.94%, and epidemiological changes contributing 189.46% (Figure 11E).

At five different SDI national levels, the total ASDR change for NDs in low SDI countries was 76,322.17, the highest among the four different SDI country categories. Population aging contributed 582.42%, population growth contributed 64.72%, while epidemiological changes contributed −547.14% (Figure 11A). In comparison to the other four SDI categories, the total ASDR changes for NPB, NE, NS, and other neonatal disorders in low SDI countries were the highest, recorded at 22,197.26, 54,011.39, 12,387.37, and −2,284.31, respectively (Figures 11B,D,F). Conversely, high SDI countries exhibited a total ASDR change for HD of −299.24, indicating the region with the highest total ASDR change for HD. Within this context, population aging contributed 11.91%, population growth contributed −3.76%, and epidemiological changes contributed 91.85% (Figure 11E).

According to the GBD 2021 database, the overall ASDR for NDs and their five etiologies has shown a global decline (Figures 11A–F). Among the 21 regions analyzed, over 50% experienced a reduction in the overall ASDR for NDs, NPB, NE, NS, and other neonatal disorders (Figures 11A–D,F). In the five countries with varying SDI levels, an increase in the overall ASDR for NDs, NPB, NE, and NS was observed only in low SDI regions; conversely, the overall ASDR for these conditions in the other four countries with different SDI levels exhibited a decline (Figures 11A–D). Globally, as well as across the 21 regions and the five countries with varying SDI levels, the overall ASDR for HD also demonstrated a decline (Figure 11E).

## 8 Risk factors for neonatal disorders

This study presents the global, five different SDI, and gender-specific ASDR and age-standardized DALYs rates of neonatal disorders caused by particulate matter pollution, short gestation, and low birth weight in 2021. In 2021, the global ASDR associated with particulate matter pollution, short gestation, and low birth weight were 8.03 (6.78 to 9.39), 16.82 (14.16 to 19.47), and 22.76 (19.63 to 26.40), respectively (Tables 6–7). Furthermore, in 2021, the global age-standardized DALYs rates attributable to particulate matter pollution, short gestational age, and low birth weight were 723.06 (610.39 to 845.18), 1693.35 (1463.77 to 1941.92), and 2227.54 (1939.96 to 2563.52), respectively (Tables 6–8).

**Table 6:** ASDR and age-standardized DALYs rates of neonatal disorders and their etiologies caused by particulate matter pollution in four global regions, 21 regions, and five different SDI countries in 2021.

Location	2021 ASDR						2021 age-standardized DALYs					
	NDs	NPB	NE	NS	HD	Other neonatal disorders	NDs	NPB	NE	NS	HD	Other neonatal disorders
Global	8.03 (6.78 to 9.39)	3.68 (3.06 to 4.39)	2.51 (2.09 to 3.03)	0.87 (0.73 to 1.04)	0.12 (0.09 to 0.16)	0.85 (0.59 to 1.06)	723.06 (610.39 to 845.18)	331.25 (275.38 to 394.95)	225.97 (187.79 to 272.36)	78.57 (65.98 to 93.19)	10.99 (8.51 to 14.47)	76.29 (53.49 to 95.40)
Sex												
Male	9.06 (7.44 to 10.84)	4.06 (3.20 to 4.94)	2.93 (2.30 to 3.65)	0.99 (0.80 to 1.21)	0.14 (0.10 to 0.19)	0.94 (0.64 to 1.2)	815.74 (669.48 to 976)	365.65 (288.28 to 445.09)	263.29 (206.68 to 328.41)	89.54 (72.30 to 109.10)	12.29 (9.13 to 16.88)	84.97 (57.66 to 107.98)
Female	6.93 (5.95 to 8.17)	3.27 (2.72 to 3.91)	2.07 (1.68 to 2.60)	0.74 (0.62 to 0.87)	0.11 (0.08 to 0.15)	0.74 (0.51 to 0.94)	623.80 (535.30 to 735.35)	294.41 (245.21 to 351.90)	185.99 (151.51 to 233.70)	66.81 (55.56 to 78.18)	9.60 (7.08 to 13.23)	66.98 (45.52 to 84.94)
Four World Regions												
Africa	12.32 (10.24 to 14.54)	4.99 (4.10 to 5.94)	4.4 (3.55 to 5.30)	1.65 (1.34 to 2.01)	0.08 (0.06 to 0.12)	1.2 (0.72 to 1.59)	1108.59 (921.93 to 1309.15)	449.25 (369.44 to 534.63)	395.61 (319.32 to 476.99)	148.41 (120.44 to 181.22)	7.14 (5.39 to 10.64)	108.17 (64.52 to 143.38)
America	1.20 (0.94 to 1.48)	0.61 (0.48 to 0.76)	0.26 (0.20 to 0.35)	0.19 (0.15 to 0.25)	0.01 (0.01 to 0.01)	0.12 (0.09 to 0.16)	107.67 (84.77 to 133.06)	54.63 (42.87 to 68.79)	23.77 (17.58 to 31.82)	17.52 (13.23 to 22.47)	0.65 (0.47 to 0.90)	11.11 (8.46 to 14.27)
Asia	7.62 (6.28 to 9.20)	3.91 (3.14 to 4.73)	2.05 (1.59 to 2.69)	0.61 (0.49 to 0.74)	0.19 (0.14 to 0.25)	0.87 (0.66 to 1.14)	686.41 (565.38 to 828.14)	351.80 (282.61 to 426.24)	184.25 (142.72 to 241.66)	54.56 (43.93 to 66.60)	17.27 (12.98 to 22.58)	78.53 (59.42 to 102.55)
Europe	0.40 (0.29 to 0.51)	0.28 (0.21 to 0.36)	0.05 (0.04 to 0.07)	0.03 (0.02 to 0.04)	0.00 (0.00 to 0.00)	0.03(0.02 to 0.05)	36.05 (26.49 to 46.38)	25.29 (18.50 to 32.18)	4.74 (3.39 to 6.22)	2.95 (2.15 to 4.01)	0.25 (0.17 to 0.36)	2.83 (1.86 to 4.16)
SDI												
High SDl	0.23 (0.18 to 0.27)	0.16 (0.14 to 0.19)	0.03 (0.02 to 0.03)	0.01 (0.01 to 0.02)	0.00 (0.00 to 0.00)	0.02 (0.01 to 0.02)	20.28 (16.65 to 23.93)	14.86 (12.21 to 17.56)	2.48 (1.96 to 3.07)	1.15 (0.92 to 1.45)	0.05 (0.04 to 0.06)	1.75 (1.30 to 2.21)
High-middle SDl	0.72 (0.59 to 0.85)	0.48 (0.39 to 0.58)	0.11 (0.09 to 0.14)	0.05 (0.04 to 0.07)	0.01 (0.01 to 0.01)	0.07 (0.04 to 0.09)	64.81 (52.88 to 76.6)	43.44 (35.56 to 51.88)	9.93 (7.69 to 12.92)	4.90 (3.87 to 6.07)	0.67 (0.48 to 0.9)	5.87 (4.00 to 7.93)
Middle SDl	3.19 (2.68 to 3.78)	1.91 (1.59 to 2.27)	0.67 (0.54 to 0.84)	0.31 (0.25 to 0.38)	0.04 (0.03 to 0.06)	0.26 (0.21 to 0.35)	287.58 (241.35 to 339.90)	171.84 (142.92 to 204.61)	60.48 (48.84 to 75.75)	27.65 (22.55 to 33.94)	3.85 (2.82 to 5.24)	23.76 (18.48 to 31.19)
Low-middle SDl	10.46 (8.61 to 12.67)	5.18 (4.19 to 6.47)	3.00 (2.37 to 3.83)	0.96 (0.77 to 1.17)	0.21 (0.15 to 0.28)	1.12 (0.83 to 1.49)	941.85 (775.15 to 1140.54)	466.79 (377.70 to 582.14)	269.87 (213.59 to 344.40)	86.11 (69.15 to 105.19)	18.65 (13.68 to 25.05)	100.42 (74.32 to 134.32)
Low SDI	14.33 (11.82 to 16.99)	5.68 (4.59 to 6.81)	5.11 (4.16 to 6.22)	1.80 (1.45 to 2.25)	0.17 (0.13 to 0.26)	1.57 (0.96 to 2.11)	1289.97 (1064.27 to 1529.20)	511.14 (413.59 to 613.22)	459.96 (374.76 to 559.48)	162.04 (130.58 to 202.81)	15.61 (11.75 to 23.16)	141.22 (86.53 to 190.15)
Regions												
Andean Latin America	1.70 (1.08 to 2.56)	0.87 (0.54 to 1.31)	0.39 (0.22 to 0.64)	0.37 (0.22 to 0.59)	0.01 (0.00 to 0.01)	0.07 (0.04 to 0.1)	153.54 (97.70 to 230.84)	78.22 (48.25 to 118.00)	35.15 (19.64 to 57.23)	33.33 (19.63 to 53.25)	0.76 (0.45 to 1.25)	6.09 (3.70 to 9.29)
Australasia	0.13 (0.02 to 0.31)	0.08 (0.02 to 0.19)	0.03 (0.00 to 0.07)	0.01 (0.00 to 0.01)	0.00 (0.00 to 0.00)	0.02 (0.00 to 0.05)	11.89 (1.74 to 28.31)	7.43 (1.43 to 16.77)	2.42 (0.20 to 6.31)	0.50 (0.11 to 1.03)	0.01 (0.00 to 0.02)	1.54 (0.13 to 4.12)
Caribbean	6.81 (4.70 to 9.07)	2.62 (1.72 to 3.79)	2.17 (1.35 to 3.36)	1.19 (0.72 to 1.80)	0.05 (0.03 to 0.11)	0.78 (0.44 to 1.19)	613.40 (423.16 to 816.49)	236.27 (154.74 to 341.48)	195.12 (121.33 to 302.24)	107.25 (65.21 to 162.36)	4.90 (2.66 to 9.51)	69.87 (39.86 to 107.47)
Central Asia	2.63 (1.92 to 3.56)	1.47 (1.07 to 1.96)	0.64 (0.44 to 0.87)	0.14 (0.10 to 0.18)	0.01 (0.01 to 0.02)	0.38 (0.23 to 0.55)	236.82 (172.8 to 320.15)	132.56 (96.62 to 176.86)	57.16 (39.24 to 78.37)	12.28 (8.95 to 16.15)	1.05 (0.72 to 1.49)	33.78 (20.91 to 49.13)
Central Europe	0.42 (0.32 to 0.53)	0.35 (0.27 to 0.44)	0.03 (0.02 to 0.05)	0.01 (0.01 to 0.01)	0.00 (0.00 to 0.00)	0.03 (0.02 to 0.04)	38.18 (29.12 to 47.78)	31.58 (24.31 to 39.61)	3.02 (2.12 to 4.07)	0.75 (0.57 to 0.98)	0.13 (0.08 to 0.20)	2.69 (1.82 to 3.85)
Central Latin America	1.35 (0.99 to 1.78)	0.79 (0.58 to 1.03)	0.22 (0.16 to 0.29)	0.23 (0.17 to 0.31)	0.01 (0.01 to 0.01)	0.10 (0.07 to 0.13)	121.68 (88.81 to 159.89)	70.90 (52.08 to 93.07)	20.06 (14.63 to 26.33)	21.11 (15.50 to 27.53)	0.67 (0.47 to 0.89)	8.95 (6.55 to 12.02)
Central Sub-Saharan Africa	9.45 (6.67 to 12.7)	3.31 (2.24 to 4.72)	4.22 (2.82 to 6.04)	0.63 (0.34 to 1.1)	0.03 (0.02 to 0.07)	1.25 (0.47 to 2.36)	850.38 (600.47 to 1143.03)	297.83 (202.1 to 424.81)	379.67 (253.80 to 543.14)	56.85 (30.66 to 98.97)	3.12 (1.60 to 6.47)	112.91 (42.47 to 212.75)
East Asia	0.89 (0.72 to 1.10)	0.62 (0.50 to 0.76)	0.20 (0.15 to 0.26)	0.02 (0.02 to 0.03)	0.01 (0.01 to 0.01)	0.04 (0.03 to 0.06)	80.36 (64.54 to 99.19)	55.49 (44.66 to 68.54)	18.26 (13.76 to 23.49)	2.20 (1.57 to 2.85)	0.74 (0.54 to 0.98)	3.68 (2.71 to 4.97)
Eastern Europe	0.28 (0.21 to 0.37)	0.19 (0.13 to 0.25)	0.04 (0.03 to 0.05)	0.04 (0.04 to 0.05)	0.00 (0.00 to 0.00)	0.01 (0.01 to 0.01)	25.38 (18.65 to 33.01)	16.94 (12.00 to 22.48)	3.45 (2.54 to 4.65)	4.00 (3.17 to 4.94)	0.21 (0.17 to 0.26)	0.78 (0.55 to 1.03)
Eastern Sub-Saharan Africa	12.65 (9.93 to 15.61)	4.29 (3.31 to 5.5)	4.40 (3.37 to 5.51)	2.06 (1.55 to 2.6)	0.09 (0.06 to 0.16)	1.82 (0.98 to 2.64)	1138.43 (893.73 to 1405.35)	386.18 (298.05 to 495.52)	395.88 (303.49 to 496.24)	184.99 (139.63 to 233.57)	7.93 (5.54 to 14.68)	163.45 (88.62 to 237.98)
High-income Asia Pacific	0.09 (0.06 to 0.13)	0.06 (0.04 to 0.08)	0.01 (0.01 to 0.02)	0.01 (0.00 to 0.01)	0.00 (0.00 to 0.00)	0.01 (0.01 to 0.02)	8.21 (5.56 to 11.49)	5.25 (3.40 to 7.65)	1.11 (0.80 to 1.48)	0.63 (0.40 to 0.96)	0.03 (0.02 to 0.04)	1.19 (0.82 to 1.66)
High-income North America	0.19 (0.13 to 0.25)	0.14 (0.09 to 0.18)	0.02 (0.02 to 0.03)	0.01 (0.01 to 0.01)	0.00 (0.00 to 0.00)	0.02 (0.02 to 0.03)	17.21 (11.93 to 22.87)	12.31 (8.41 to 16.44)	2.01 (1.37 to 2.77)	0.81 (0.60 to 1.05)	0.01 (0.01 to 0.02)	2.06 (1.36 to 2.81)
North Africa and Middle East	3.37 (2.66 to 4.20)	2.24 (1.74 to 2.83)	0.65 (0.45 to 0.89)	0.12 (0.09 to 0.16)	0.04 (0.03 to 0.08)	0.31 (0.17 to 0.47)	303.08 (239.40 to 377.89)	201.25 (156.29 to 255.18)	58.69 (40.41 to 80.06)	10.87 (7.80 to 14.51)	4.07 (2.54 to 6.99)	28.20 (15.20 to 42.45)
Oceania	6.46 (4.25 to 8.81)	3.76 (2.42 to 5.45)	1.35 (0.79 to 2.02)	0.39 (0.21 to 0.65)	0.00 (0.00 to 0.02)	0.96 (0.53 to 1.58)	581.73 (382.39 to 792.64)	338.61 (217.86 to 490.72)	121.35 (71.39 to 181.44)	34.96 (19.06 to 58.51)	0.33 (0.11 to 1.46)	86.47 (47.44 to 142.13)
South Asia	12.75 (10.41 to 15.54)	6.37 (5.01 to 7.91)	3.52 (2.68 to 4.82)	0.97 (0.77 to 1.22)	0.35 (0.26 to 0.47)	1.53 (1.14 to 2.02)	1148.10 (937.41 to 1398.69)	573.68 (451.33 to 711.78)	316.98 (241.00 to 433.87)	87.36 (68.96 to 109.46)	32.00 (23.81 to 42.17)	138.08 (102.34 to 182.04)
Southeast Asia	3.66 (2.94 to 4.45)	2.08 (1.68 to 2.52)	0.73 (0.50 to 0.96)	0.56 (0.43 to 0.74)	0.04 (0.03 to 0.06)	0.25 (0.17 to 0.43)	329.59 (264.31 to 400.78)	186.93 (150.97 to 227.2)	66.02 (45.30 to 86.52)	50.16 (38.41 to 66.79)	3.73 (2.77 to 5.05)	22.75 (15.42 to 38.70)
Southern Latin America	0.68 (0.28 to 1.10)	0.51 (0.22 to 0.82)	0.05 (0.02 to 0.09)	0.06 (0.03 to 0.09)	0.00 (0.00 to 0.00)	0.05 (0.02 to 0.08)	60.97 (25.66 to 98.62)	46.32 (19.80 to 73.65)	4.79 (2.09 to 7.97)	5.20 (2.50 to 8.27)	0.16 (0.06 to 0.28)	4.51 (1.90 to 7.52)
Southern Sub-Saharan Africa	7.30 (5.57 to 9.56)	3.77 (2.84 to 5.04)	1.43 (1.00 to 2.01)	0.69 (0.51 to 0.96)	0.05 (0.04 to 0.07)	1.36 (0.95 to 1.94)	657.50 (501.38 to 859.97)	339.63 (256.04 to 453.79)	128.57 (89.93 to 180.74)	61.78 (46.02 to 85.98)	4.83 (3.48 to 6.56)	122.68 (85.06 to 174.31)
Tropical Latin America	0.88 (0.63 to 1.16)	0.43 (0.30 to 0.57)	0.17 (0.12 to 0.23)	0.11 (0.08 to 0.15)	0.01 (0.00 to 0.01)	0.16 (0.11 to 0.21)	79.37 (56.58 to 104.25)	38.78 (27.29 to 51.71)	15.51 (10.68 to 20.98)	10.19 (7.35 to 13.42)	0.50 (0.35 to 0.70)	14.39 (10.31 to 19.14)
Western Europe	0.17 (0.12 to 0.24)	0.12 (0.09 to 0.17)	0.03 (0.02 to 0.04)	0.01 (0.01 to 0.01)	0.00 (0.00 to 0.00)	0.02 (0.01 to 0.02)	15.58 (10.86 to 21.35)	10.96 (7.78 to 14.95)	2.34 (1.50 to 3.35)	0.78 (0.52 to 1.10)	0.02 (0.02 to 0.04)	1.48 (0.92 to 2.18)
Western Sub-Saharan Africa	16.23 (13.84 to 18.82)	6.89 (5.65 to 8.16)	6.03 (4.91 to 7.25)	2.18 (1.79 to 2.64)	0.10 (0.08 to 0.14)	1.02 (0.68 to 1.31)	1460.55 (1246.20 to 1694.11)	620.04 (509.12 to 734.24)	542.98 (441.53 to 652.26)	196.06 (160.90 to 237.16)	9.44 (7.18 to 12.90)	92.03 (61.37 to 118.15)

**Table 7:** ASDR and age-standardized DALYs rate of neonatal disorders and five etiologies of neonatal disorders caused by short gestational age in four global regions, 21 regions, and five SDI countries in 2021.

Location	2021 ASDR						2021 age-standardized DALYs					
	NDs	NPB	NE	NS	HD	Other neonatal disorders	NDs	NPB	NE	NS	HD	Other neonatal disorders
Global	16.82 (14.16 to 19.47)	11.93 (10.06 to 14.10)	2.75 (2.18 to 3.44)	0.99 (0.80 to 1.19)	0.15 (0.11 to 0.20)	0.99 (0.67 to 1.27)	1693.35 (1463.77 to 1941.92)	1253.53 (1089.75 to 1462.17)	247.85 (195.73 to 309.53)	88.90 (72.03 to 106.89)	13.76 (10.31 to 18.30)	89.31 (60.67 to 114.35)
Sex												
Male	18.71 (15.41 to 22.03)	13.08 (10.64 to 15.76)	3.22 (2.40 to 4.20)	1.12 (0.87 to 1.42)	0.17 (0.13 to 0.24)	1.12 (0.76 to 1.46)	1874.11 (1587.64 to 2190.23)	1368.00 (1158.38 to 1620.89)	289.44 (215.93 to 378.31)	100.75 (78.03 to 127.81)	15.40 (11.33 to 21.21)	100.52 (67.95 to 131.33)
Female	14.80 (12.50 to 17.22)	10.70 (8.87 to 12.42)	2.26 (1.70 to 2.98)	0.85 (0.68 to 1.06)	0.13 (0.09 to 0.19)	0.86 (0.56 to 1.13)	1499.85 (1283.91 to 1726.50)	1131.03 (959.56 to 1291.81)	203.31 (153.29 to 268.39)	76.21 (60.95 to 95.58)	11.99 (8.33 to 17.21)	77.31 (50.15 to 101.83)
Four World Regions												
Africa	22.84 (18.70 to 26.98)	15.63 (12.90 to 18.48)	4.28 (3.14 to 5.37)	1.65 (1.27 to 2.04)	0.08 (0.06 to 0.13)	1.20 (0.66 to 1.69)	2166.12 (1801.10 to 2543.61)	1517.82 (1294.99 to 1777.63)	384.76 (282.58 to 483.03)	148.75 (114.17 to 183.66)	7.09 (5.16 to 11.26)	107.71 (59.70 to 151.84)
America	6.09 (5.05 to 7.35)	5.12 (4.23 to 6.17)	0.40 (0.29 to 0.53)	0.31 (0.23 to 0.40)	0.01 (0.01 to 0.02)	0.25 (0.18 to 0.32)	685.33 (582.75 to 797.37)	598.27 (511.24 to 697.41)	36.12 (25.96 to 47.34)	27.78 (21.09 to 35.85)	0.98 (0.68 to 1.40)	22.19 (15.91 to 28.72)
Asia	16.94 (14.26 to 20.04)	12.15 (9.96 to 14.51)	2.59 (1.96 to 3.45)	0.81 (0.65 to 0.99)	0.25 (0.19 to 0.34)	1.14 (0.84 to 1.49)	1749.30 (1509.46 to 2033.76)	1318.31 (1116.48 to 1552.18)	232.93 (176.33 to 310.78)	72.67 (58.22 to 89.28)	22.81 (16.83 to 30.34)	102.58 (75.24 to 133.87)
Europe	2.95(2.55 to 3.39)	2.77 (2.39 to 3.18)	0.07 (0.04 to 0.10)	0.06 (0.04 to 0.08)	0.00 (0.00 to 0.01)	0.05 (0.03 to 0.07)	370.06 (325.08 to 415.98)	353.91 (311.61 to 397.81)	6.45 (3.68 to 9.36)	5.19 (3.65 to 6.79)	0.35 (0.21 to 0.52)	4.15 (2.30 to 6.17)
SDI												
High SDl	2.53 (2.27 to 2.76)	2.39 (2.13 to 2.60)	0.06 (0.04 to 0.08)	0.03 (0.02 to 0.03)	0.00 (0.00 to 0.00)	0.05 (0.03 to 0.07)	347.61 (307.94 to 387.14)	334.93 (296.56 to 374.39)	5.47 (3.42 to 7.56)	2.53 (1.89 to 3.11)	0.07 (0.05 to 0.09)	4.61 (2.84 to 6.46)
High-middle SDl	3.41 (2.99 to 3.85)	3.14 (2.74 to 3.55)	0.10 (0.07 to 0.14)	0.08 (0.06 to 0.10)	0.01 (0.01 to 0.01)	0.08 (0.05 to 0.12)	408.21 (365.05 to 456.91)	383.35 (341.96 to 431.50)	9.13 (6.32 to 12.65)	7.27 (5.44 to 9.16)	0.89 (0.65 to 1.22)	7.57 (4.8 to 10.72)
Middle SDl	9.57 (8.15 to 11.14)	8.06 (6.75 to 9.36)	0.70 (0.50 to 0.95)	0.39 (0.30 to 0.48)	0.06 (0.04 to 0.08)	0.36 (0.26 to 0.48)	1023.01 (889.14 to 1177.20)	886.75 (764.52 to 1022.05)	63.26 (45.11 to 85.56)	35.13 (26.99 to 43.63)	5.22 (3.72 to 7.10)	32.64 (23.8 to 43.25)
Low-middle SDl	22.41 (18.82 to 26.4)	16.06 (13.19 to 19.09)	3.53 (2.65 to 4.61)	1.18 (0.94 to 1.48)	0.26 (0.19 to 0.36)	1.39 (1.00 to 1.85)	2289.95 (1965.39 to 2676.84)	1717.86 (1451.52 to 2008.82)	317.21 (238.55 to 414.81)	106.00 (84.77 to 132.81)	23.68 (16.96 to 31.97)	125.20 (90.21 to 166.29)
Low SDI	25.77 (21.08 to 30.85)	16.61 (13.66 to 19.93)	5.38 (4.15 to 6.78)	1.89 (1.43 to 2.37)	0.21 (0.15 to 0.32)	1.69 (0.99 to 2.37)	2490.52 (2072.93 to 2954.39)	1666.04 (1422.31 to 1973.34)	484.08 (373.05 to 610.14)	169.75 (128.53 to 213.06)	18.79 (13.67 to 28.72)	151.86 (88.67 to 212.99)
Regions												
Andean Latin America	5.68 (4.41 to 7.20)	5.01 (3.84 to 6.31)	0.30 (0.10 to 0.55)	0.32 (0.16 to 0.52)	0.01 (0.00 to 0.01)	0.04 (0.02 to 0.06)	617.58 (498.81 to 754.43)	557.60 (452.33 to 672.65)	27.21 (9.41 to 49.88)	29.13 (14.24 to 46.80)	0.46 (0.19 to 0.87)	3.18 (1.38 to 5.48)
Australasia	1.91 (1.62 to 2.25)	1.67 (1.42 to 1.97)	0.13 (0.09 to 0.18)	0.03 (0.02 to 0.03)	0.00 (0.00 to 0.00)	0.09 (0.06 to 0.12)	295.74 (253.65 to 347.38)	273.81 (232.00 to 322.26)	11.84 (7.80 to 15.96)	2.39 (1.76 to 3.11)	0.02 (0.01 to 0.04)	7.67 (5.10 to 10.55)
Caribbean	16.97 (13.79 to 20.81)	10.60 (8.30 to 13.58)	3.26 (2.28 to 4.53)	1.85 (1.25 to 2.56)	0.08 (0.05 to 0.15)	1.19 (0.74 to 1.78)	1697.86 (1410.49 to 2042.71)	1124.24 (905.35 to 1388.46)	293.18 (204.71 to 407.70)	166.22 (112.77 to 230.09)	7.34 (4.27 to 13.23)	106.89 (66.75 to 159.76)
Central Asia	8.18 (6.84 to 9.69)	7.11 (5.97 to 8.37)	0.58 (0.39 to 0.81)	0.13 (0.10 to 0.17)	0.01 (0.01 to 0.02)	0.34 (0.22 to 0.48)	819.19 (699.77 to 954.29)	723.70 (618.10 to 838.60)	52.18 (34.76 to 72.45)	11.89 (8.68 to 15.53)	0.88 (0.52 to 1.42)	30.55 (20.13 to 43.30)
Central Europe	2.88 (2.43 to 3.35)	2.84 (2.39 to 3.30)	0.02 (0.01 to 0.03)	0.00 (0.00 to 0.01)	0.00 (0.00 to 0.00)	0.02 (0.01 to 0.03)	365.06 (317.80 to 414.54)	361.58 (314.60 to 411.61)	1.48 (0.78 to 2.33)	0.36 (0.23 to 0.51)	0.08 (0.03 to 0.16)	1.56 (0.66 to 2.97)
Central Latin America	6.62 (5.23 to 8.37)	6.10 (4.80 to 7.72)	0.17 (0.10 to 0.25)	0.24 (0.17 to 0.33)	0.01 (0.00 to 0.01)	0.10 (0.06 to 0.14)	706.56 (578.78 to 863.98)	660.24 (542.37 to 804.14)	15.25 (9.13 to 22.92)	21.55 (15.18 to 29.32)	0.64 (0.41 to 0.92)	8.89 (5.47 to 12.78)
Central Sub-Saharan Africa	16.24 (12.31 to 20.01)	10.58 (8.10 to 13.43)	3.86 (2.35 to 5.48)	0.60 (0.29 to 1.00)	0.03 (0.02 to 0.06)	1.17 (0.40 to 2.32)	1537.35 (1184.05 to 1882.70)	1027.91 (812.76 to 1280.42)	347.42 (211.30 to 492.85)	53.68 (26.35 to 90.36)	2.87 (1.43 to 5.80)	105.48 (35.76 to 208.64)
East Asia	2.72 (2.28 to 3.24)	2.69 (2.26 to 3.20)	0.02 (0.01 to 0.04)	0.00 (0.00 to 0.01)	0.00 (0.00 to 0.00)	0.01 (0.00 to 0.01)	308.43 (265.05 to 358.28)	305.67 (263.18 to 355.42)	1.67 (0.60 to 3.25)	0.39 (0.22 to 0.61)	0.12 (0.06 to 0.20)	0.59 (0.30 to 0.96)
Eastern Europe	2.47 (2.21 to 2.73)	2.27 (2.03 to 2.52)	0.07 (0.05 to 0.09)	0.11 (0.08 to 0.13)	0.01 (0.00 to 0.01)	0.02 (0.01 to 0.02)	296.81 (269.87 to 327.31)	279.09 (252.98 to 309.19)	6.24 (4.20 to 8.06)	9.51 (7.47 to 11.43)	0.50 (0.38 to 0.62)	1.47 (1.06 to 1.85)
Eastern Sub-Saharan Africa	20.56 (16.35 to 25.43)	11.93 (9.44 to 14.89)	4.55 (3.42 to 5.98)	2.13 (1.60 to 2.78)	0.09 (0.06 to 0.19)	1.86 (0.96 to 2.71)	1969.85 (1597.51 to 2414.44)	1192.53 (976.41 to 1459.17)	409.27 (308.06 to 537.62)	191.99 (144.05 to 249.73)	8.30 (5.60 to 16.81)	167.75 (86.12 to 244.11)
High-income Asia Pacific	0.75 (0.68 to 0.84)	0.69 (0.62 to 0.77)	0.03 (0.02 to 0.03)	0.01 (0.01 to 0.01)	0.00 (0.00 to 0.00)	0.03 (0.02 to 0.03)	149.14 (123.48 to 173.30)	143.31 (117.85 to 167.24)	2.47 (1.89 to 3.06)	0.97 (0.76 to 1.20)	0.05 (0.03 to 0.06)	2.34 (1.78 to 2.98)
High-income North America	3.54 (3.15 to 3.93)	3.34 (2.98 to 3.72)	0.07 (0.04 to 0.11)	0.05 (0.04 to 0.06)	0.00 (0.00 to 0.00)	0.08 (0.04 to 0.11)	467.97 (415.23 to 521.78)	450.20 (398.75 to 502.69)	6.67 (3.88 to 9.69)	4.21 (3.24 to 5.13)	0.06 (0.04 to 0.08)	6.84 (3.91 to 10.03)
North Africa and Middle East	10.54 (8.86 to 12.36)	9.17 (7.73 to 10.79)	0.85 (0.60 to 1.12)	0.14 (0.09 to 0.19)	0.05 (0.03 to 0.09)	0.34 (0.17 to 0.55)	1100.70 (943.54 to 1269.55)	976.91 (842.40 to 1138.27)	76.34 (54.33 to 100.43)	12.54 (8.10 to 17.20)	4.63 (2.72 to 8.13)	30.30 (15.11 to 49.23)
Oceania	13.82 (10.94 to 17.17)	10.73 (8.22 to 13.68)	1.54 (0.98 to 2.25)	0.45 (0.25 to 0.76)	0.00 (0.00 to 0.02)	1.10 (0.64 to 1.78)	1382.30 (1113.74 to 1692.48)	1103.70 (868.49 to 1365.65)	138.26 (88.01 to 202.42)	40.82 (22.78 to 67.94)	0.37 (0.12 to 1.58)	99.15 (58.00 to 159.98)
South Asia	27.13 (22.59 to 32.29)	18.52 (14.83 to 22.60)	4.66 (3.46 to 6.43)	1.38 (1.08 to 1.73)	0.48 (0.36 to 0.64)	2.08 (1.51 to 2.74)	2837.44 (2444.84 to 3327.29)	2063.31 (1720.78 to 2464.52)	419.42 (311.56 to 578.82)	124.22 (97.17 to 155.64)	43.43 (32.13 to 57.25)	187.05 (135.90 to 246.60)
Southeast Asia	9.44 (7.76 to 11.08)	8.12 (6.64 to 9.58)	0.53 (0.31 to 0.75)	0.55 (0.41 to 0.71)	0.03 (0.02 to 0.04)	0.21 (0.14 to 0.31)	969.51 (809.21 to 1115.97)	851.23 (720.58 to 991.27)	47.68 (27.70 to 67.32)	49.07 (36.92 to 64)	2.77 (1.79 to 4.02)	18.76 (12.82 to 27.93)
Southern Latin America	5.16 (4.05 to 6.44)	5.07 (4.00 to 6.33)	0.03 (0.01 to 0.05)	0.04 (0.02 to 0.08)	0.00 (0.00 to 0.00)	0.03 (0.01 to 0.05)	593.64 (493.48 to 706.79)	585.02 (486.45 to 694.08)	2.25 (0.74 to 4.81)	3.97 (1.73 to 6.97)	0.08 (0.03 to 0.18)	2.31 (0.90 to 4.50)
Southern Sub-Saharan Africa	19.91 (16 to 24.51)	15.60 (12.55 to 19.26)	1.71 (1.14 to 2.46)	0.86 (0.59 to 1.16)	0.07 (0.05 to 0.10)	1.66 (1.07 to 2.36)	1910.23 (1558.16 to 2328.58)	1522.77 (1253.00 to 1847.87)	154.25 (102.58 to 221.07)	77.24 (53.10 to 104.47)	6.43 (4.31 to 9.01)	149.53 (96.39 to 212.04)
Tropical Latin America	6.43 (5.17 to 7.96)	4.94 (3.98 to 6.11)	0.53 (0.37 to 0.71)	0.41 (0.30 to 0.52)	0.02 (0.01 to 0.02)	0.53 (0.37 to 0.69)	733.01 (609.33 to 871.84)	599.12 (501.67 to 710.19)	47.91 (33.11 to 63.61)	36.71 (27.32 to 47.06)	1.44 (0.99 to 1.96)	47.84 (33.73 to 61.66)
Western Europe	1.96 (1.72 to 2.18)	1.86 (1.64 to 2.07)	0.05 (0.03 to 0.08)	0.02 (0.01 to 0.02)	0.00 (0.00 to 0.00)	0.03 (0.02 to 0.05)	281.98 (246.92 to 318.43)	272.73 (236.51 to 308.34)	4.95 (2.99 to 7.06)	1.38 (0.88 to 1.90)	0.04 (0.02 to 0.06)	2.88 (1.63 to 4.13)
Western Sub-Saharan Africa	30.50 (25.2 to 36.05)	21.56 (17.91 to 25.85)	5.72 (4.15 to 7.22)	2.13 (1.67 to 2.62)	0.10 (0.07 to 0.14)	0.99 (0.62 to 1.34)	2834.99 (2365.27 to 3330.37)	2030.10 (1700.24 to 2407.78)	514.92 (373.27 to 649.73)	191.28 (150.55 to 236.04)	9.22 (6.74 to 12.94)	89.48 (55.95 to 120.68)

**Table 8:** ASDR and age-standardized DALYs rates of neonatal disorders and their etiologies caused by low birth weight in four global regions, 21 regions, and five different SDI countries in 2021.

Location	2021 ASDR						2021 age-standardized DALYs					
	NDs	NPB	NE	NS	HD	Other neonatal disorders	NDs	NPB	NE	NS	HD	Other neonatal disorders
Global	22.76 (19.63 to 26.40)	11.93 (10.06 to 14.1)	6.15 (5.20 to 7.33)	2.21 (1.88 to 2.60)	0.32 (0.25 to 0.41)	2.15 (1.55 to 2.62)	2227.54 (1939.96 to 2563.52)	1253.53 (1089.75 to 1462.17)	553.03 (468.11 to 659.68)	198.94 (169.45 to 234.07)	28.70 (22.38 to 36.63)	193.35 (139.45 to 236.05)
Sex												
Male	25.36 (21.56 to 29.89)	13.08 (10.64 to 15.76)	7.07 (5.78 to 8.53)	2.49 (2.07 to 3.01)	0.35 (0.27 to 0.47)	2.36 (1.67 to 2.97)	2472.64 (2125.59 to 2897.50)	1368.00 (1158.38 to 1620.89)	636.26 (520.22 to 767.67)	223.84 (186.24 to 270.52)	31.87 (24.58 to 42.14)	212.67 (150.43 to 267.08)
Female	19.97 (17.32 to 22.82)	10.70 (8.87 to 12.42)	5.16 (4.27 to 6.33)	1.91 (1.62 to 2.22)	0.28 (0.21 to 0.38)	1.92 (1.37 to 2.38)	1965.13 (1711.55 to 2231.66)	1131.03 (959.56 to 1291.81)	463.87 (383.90 to 569.37)	172.26 (145.68 to 199.30)	25.30 (18.94 to 34.15)	172.66 (123.03 to 213.72)
Four World Regions												
Africa	32.12 (27.15 to 37.86)	15.63 (12.90 to 18.48)	9.76 (7.97 to 11.71)	3.79 (3.11 to 4.6)	0.18 (0.14 to 0.28)	2.76 (1.64 to 3.61)	3001.71 (2547.67 to 3519.28)	1517.82 (1294.99 to 1777.63)	878.14 (717.19 to 1053.80)	340.84 (279.89 to 413.67)	16.54 (12.60 to 24.75)	248.37 (147.73 to 324.81)
America	8.19 (6.70 to 9.88)	5.12 (4.23 to 6.17)	1.23 (1.00 to 1.54)	1.05 (0.83 to 1.30)	0.03 (0.03 to 0.04)	0.75 (0.61 to 0.91)	873.65 (740.85 to 1025.81)	598.27 (511.24 to 697.41)	111.08 (90.23 to 138.16)	94.15 (74.94 to 116.62)	3.07 (2.41 to 3.97)	67.08 (55.21 to 81.71)
Asia	22.13 (18.87 to 26.06)	12.15 (9.96 to 14.51)	5.53 (4.45 to 7.02)	1.65 (1.36 to 1.98)	0.51 (0.39 to 0.66)	2.29 (1.77 to 2.96)	2216.46 (1921.56 to 2575.36)	1318.31 (1116.48 to 1552.18)	497.21 (400.25 to 631.29)	148.67 (122.27 to 177.73)	46.07 (35.05 to 59.26)	206.19 (159.20 to 266.25)
Europe	3.65 (3.16 to 4.21)	2.77 (2.39 to 3.18)	0.38 (0.32 to 0.45)	0.26 (0.22 to 0.31)	0.02 (0.01 to 0.02)	0.22 (0.18 to 0.27)	432.96 (383.87 to 488.09)	353.91 (311.61 to 397.81)	34.29 (28.98 to 40.16)	22.99 (19.47 to 27.56)	1.60 (1.27 to 1.99)	20.17 (16.09 to 24.73)
SDI												
High SDl	3.02 (2.70 to 3.32)	2.39 (2.13 to 2.60)	0.28 (0.24 to 0.32)	0.12 (0.11 to 0.13)	0.00 (0.00 to 0.00)	0.23 (0.20 to 0.26)	392.10 (350.03 to 435.11)	334.93 (296.56 to 374.39)	25.20 (21.85 to 28.56)	10.82 (9.56 to 12.12)	0.29 (0.25 to 0.33)	20.85 (18.16 to 23.74)
High-middle SDl	4.23 (3.72 to 4.77)	3.14 (2.74 to 3.55)	0.47 (0.39 to 0.57)	0.32 (0.27 to 0.37)	0.03 (0.02 to 0.04)	0.28 (0.22 to 0.35)	482.13 (429.84 to 538.63)	383.35 (341.96 to 431.50)	42.10 (35.28 to 51.36)	28.44 (24.05 to 33.41)	2.68 (2.15 to 3.39)	25.56 (19.66 to 31.30)
Middle SDl	12.37 (10.67 to 14.40)	8.06 (6.75 to 9.36)	2.13 (1.75 to 2.60)	1.12 (0.93 to 1.34)	0.13 (0.10 to 0.17)	0.93 (0.75 to 1.19)	1274.50 (1102.94 to 1466.91)	886.75 (764.52 to 1022.05)	191.29 (157.90 to 234.15)	100.81 (84.07 to 120.91)	12.04 (9.28 to 15.57)	83.62 (67.17 to 107.19)
Low-middle SDl	30.13 (25.86 to 35.19)	16.06 (13.19 to 19.09)	8.02 (6.51 to 9.80)	2.53 (2.06 to 3.10)	0.56 (0.42 to 0.76)	2.96 (2.26 to 3.85)	2983.76 (2589.67 to 3440.51)	1717.86 (1451.52 to 2008.82)	721.72 (585.94 to 882.03)	227.69 (185.22 to 278.85)	50.49 (37.86 to 68.06)	265.99 (202.96 to 346.85)
Low SDI	35.82 (30.10 to 42.65)	16.61 (13.66 to 19.93)	11.22 (9.25 to 13.59)	4.06 (3.26 to 5.04)	0.41 (0.31 to 0.60)	3.52 (2.11 to 4.66)	3394.35 (2877.98 to 4006.16)	1666.04 (1422.31 to 1973.34)	1009.93 (832.51 to 1222.70)	365.02 (293.41 to 453.39)	36.70 (27.84 to 53.60)	316.65 (189.75 to 419.08)
Regions												
Andean Latin America	8.40 (6.59 to 10.36)	5.01 (3.84 to 6.31)	1.52 (1.10 to 2.06)	1.51 (1.11 to 2.04)	0.03 (0.02 to 0.05)	0.32 (0.23 to 0.42)	862.45 (697.31 to 1041.51)	557.60 (452.33 to 672.65)	137.15 (99.04 to 185.58)	136.31 (99.81 to 183.19)	2.93 (2.00 to 4.13)	28.46 (20.88 to 37.44)
Australasia	2.35 (1.99 to 2.78)	1.67 (1.42 to 1.97)	0.37 (0.30 to 0.45)	0.08 (0.07 to 0.10)	0.00 (0.00 to 0.00)	0.24 (0.19 to 0.29)	335.51 (288.12 to 390.62)	273.81 (232.00 to 322.26)	33.12 (27.08 to 40.06)	7.35 (6.08 to 8.83)	0.06 (0.04 to 0.09)	21.16 (17.10 to 25.79)
Caribbean	21.97 (17.75 to 26.46)	10.60 (8.30 to 13.58)	5.54 (4.06 to 7.46)	3.67 (2.63 to 4.91)	0.15 (0.10 to 0.24)	2.01 (1.34 to 2.90)	2147.81 (1769.26 to 2554.55)	1124.24 (905.35 to 1388.46)	498.66 (365.5 to 671.42)	330.53 (236.90 to 441.87)	13.35 (8.70 to 21.95)	181.03 (121.01 to 261.23)
Central Asia	11.06 (9.32 to 13.00)	7.11 (5.97 to 8.37)	2.18 (1.80 to 2.64)	0.54 (0.44 to 0.65)	0.05 (0.03 to 0.06)	1.18 (0.91 to 1.46)	1078.61 (921.94 to 1254.72)	723.70 (618.10 to 838.60)	196.52 (162.24 to 237.13)	48.44 (39.85 to 58.70)	4.05 (3.00 to 5.29)	105.90 (82.25 to 131.41)
Central Europe	3.26 (2.76 to 3.78)	2.84 (2.39 to 3.30)	0.18 (0.15 to 0.22)	0.05 (0.04 to 0.06)	0.01 (0.01 to 0.01)	0.17 (0.13 to 0.22)	399.04 (347.50 to 453.14)	361.58 (314.60 to 411.61)	16.53 (13.36 to 19.82)	4.56 (3.65 to 5.63)	0.75 (0.54 to 1.06)	15.61 (12.06 to 19.63)
Central Latin America	9.28 (7.37 to 11.73)	6.10 (4.80 to 7.72)	1.19 (0.93 to 1.50)	1.42 (1.13 to 1.80)	0.04 (0.03 to 0.05)	0.53 (0.42 to 0.68)	946.32 (774.37 to 1166.73)	660.24 (542.37 to 804.14)	106.90 (84.09 to 135.33)	127.82 (101.32 to 161.64)	3.56 (2.75 to 4.63)	47.80 (37.43 to 61.58)
Central Sub-Saharan Africa	25.33 (20.42 to 30.44)	10.58 (8.10 to 13.43)	10.01 (7.92 to 12.67)	1.60 (0.95 to 2.66)	0.08 (0.05 to 0.17)	3.05 (1.25 to 5.53)	2354.78 (1925.37 to 2821.34)	1027.91 (812.76 to 1280.42)	900.93 (712.26 to 1139.75)	144.00 (85.33 to 239.30)	7.54 (4.23 to 15.06)	274.40 (112.79 to 497.3)
East Asia	3.53 (3.01 to 4.17)	2.69 (2.26 to 3.20)	0.60 (0.48 to 0.75)	0.08 (0.06 to 0.10)	0.03 (0.02 to 0.04)	0.13 (0.10 to 0.17)	381.33 (331.70 to 440.89)	305.67 (263.18 to 355.42)	54.19 (43.58 to 67.30)	7.40 (5.54 to 9.24)	2.48 (1.89 to 3.22)	11.59 (8.72 to 15.03)
Eastern Europe	3.17 (2.85 to 3.52)	2.27 (2.03 to 2.52)	0.35 (0.31 to 0.40)	0.44 (0.39 to 0.50)	0.02 (0.02 to 0.03)	0.08 (0.07 to 0.09)	360.01 (328.44 to 396.63)	279.09 (252.98 to 309.19)	31.69 (27.81 to 35.92)	39.99 (35.40 to 45.16)	2.11 (1.85 to 2.41)	7.13 (6.19 to 8.20)
Eastern Sub-Saharan Africa	30.16 (24.56 to 37.23)	11.93 (9.44 to 14.89)	9.48 (7.57 to 12.03)	4.59 (3.55 to 5.84)	0.20 (0.13 to 0.38)	3.97 (2.14 to 5.59)	2833.52 (2322.60 to 3469.22)	1192.53 (976.41 to 1459.17)	853.31 (681.06 to 1082.00)	413.18 (319.72 to 525.82)	17.64 (12.14 to 34.28)	356.85 (192.45 to 502.52)
High-income Asia Pacific	0.97 (0.88 to 1.08)	0.69 (0.62 to 0.77)	0.11 (0.10 to 0.12)	0.06 (0.05 to 0.07)	0.00 (0.00 to 0.00)	0.11 (0.10 to 0.13)	168.93 (142.50 to 194.60)	143.31 (117.85 to 167.24)	9.98 (8.84 to 11.17)	5.10 (4.33 to 5.99)	0.22 (0.18 to 0.26)	10.32 (8.84 to 11.79)
High-income North America	4.23 (3.77 to 4.70)	3.34 (2.98 to 3.72)	0.36 (0.31 to 0.41)	0.16 (0.15 to 0.18)	0.00 (0.00 to 0.00)	0.36 (0.32 to 0.42)	530.20 (471.22 to 588.76)	450.20 (398.75 to 502.69)	32.22 (28.02 to 36.64)	14.76 (13.05 to 16.38)	0.24 (0.19 to 0.30)	32.78 (28.48 to 37.65)
North Africa and Middle East	12.24 (10.48 to 14.33)	9.17 (7.73 to 10.79)	1.68 (1.32 to 2.05)	0.40 (0.30 to 0.51)	0.12 (0.08 to 0.20)	0.88 (0.51 to 1.22)	1253.53 (1082.88 to 1440.16)	976.91 (842.40 to 1138.27)	150.83 (118.68 to 184.38)	35.99 (27.25 to 45.67)	10.97 (7.61 to 17.86)	78.83 (45.57 to 109.35)
Oceania	16.95 (13.61 to 20.72)	10.73 (8.22 to 13.68)	3.09 (2.12 to 4.28)	0.92 (0.54 to 1.49)	0.01 (0.00 to 0.04)	2.20 (1.38 to 3.42)	1663.52 (1351.07 to 2006.75)	1103.70 (868.49 to 1365.65)	277.92 (190.98 to 385.52)	83.22 (48.39 to 133.69)	0.73 (0.25 to 3.39)	197.95 (123.76 to 307.79)
South Asia	35.43 (29.83 to 42.02)	18.52 (14.83 to 22.60)	9.44 (7.42 to 12.41)	2.54 (2.05 to 3.11)	0.94 (0.71 to 1.24)	3.98 (3.01 to 5.17)	3584.81 (3092.96 to 4209.78)	2063.31 (1720.78 to 2464.52)	849.77 (667.17 to 1116.22)	228.54 (184.37 to 279.47)	84.98 (64.01 to 111.77)	358.21 (270.49 to 465.40)
Southeast Asia	12.93 (10.84 to 15.16)	8.12 (6.64 to 9.58)	2.18 (1.51 to 2.76)	1.75 (1.37 to 2.30)	0.12 (0.09 to 0.15)	0.76 (0.54 to 1.28)	1283.24 (1,098 to 1478.69)	851.23 (720.58 to 991.27)	195.77 (135.74 to 248.75)	157.04 (123.42 to 206.51)	10.90 (8.35 to 13.89)	68.30 (48.78 to 115.46)
Southern Latin America	6.22 (4.89 to 7.81)	5.07 (4.00 to 6.33)	0.36 (0.27 to 0.46)	0.44 (0.34 to 0.58)	0.01 (0.01 to 0.02)	0.34 (0.26 to 0.45)	688.72 (569.58 to 827.73)	585.02 (486.45 to 694.08)	32.05 (24.57 to 41.74)	39.73 (30.61 to 51.77)	1.09 (0.75 to 1.53)	30.84 (23.84 to 40.57)
Southern Sub-Saharan Africa	27.70 (22.82 to 33.73)	15.60 (12.55 to 19.26)	4.83 (3.71 to 6.30)	2.37 (1.87 to 2.97)	0.19 (0.14 to 0.25)	4.71 (3.56 to 6.14)	2611.63 (2165.64 to 3152.89)	1522.77 (1253.00 to 1847.87)	434.24 (334.00 to 566.90)	213.54 (168.18 to 266.79)	17.13 (12.72 to 22.13)	423.95 (320.28 to 552.48)
Tropical Latin America	8.88 (7.14 to 11.07)	4.94 (3.98 to 6.11)	1.45 (1.16 to 1.82)	1.04 (0.82 to 1.33)	0.04 (0.03 to 0.06)	1.40 (1.13 to 1.76)	953.56 (784.96 to 1144.17)	599.12 (501.67 to 710.19)	130.40 (104.64 to 163.43)	93.96 (73.99 to 119.53)	4.05 (3.13 to 5.25)	126.04 (101.94 to 158.31)
Western Europe	2.42 (2.13 to 2.72)	1.86 (1.64 to 2.07)	0.28 (0.24 to 0.33)	0.10 (0.09 to 0.12)	0.00 (0.00 to 0.00)	0.18 (0.15 to 0.21)	323.33 (282.23 to 362.16)	272.73 (236.51 to 308.34)	25.22 (21.23 to 29.36)	9.15 (7.73 to 10.70)	0.25 (0.20 to 0.30)	15.99 (13.56 to 18.79)
Western Sub-Saharan Africa	42.31 (35.99 to 49.29)	21.56 (17.91 to 25.85)	13.26 (10.76 to 15.83)	4.98 (4.12 to 5.97)	0.24 (0.18 to 0.32)	2.27 (1.54 to 2.88)	3896.93 (3333.59 to 4519.7)	2030.10 (1700.24 to 2407.78)	1193.10 (967.89 to 1424.34)	447.72 (370.93 to 536.92)	21.42 (16.43 to 28.74)	204.59 (138.62 to 259.09)

In 2021, low birth weight emerged as the most significant risk factor, resulting in the highest ASDR and age-standardized DALYs rate for NDs, NE, NS, HD, and other neonatal disorders globally, across four world regions, in 21 regions, and in five different SDI countries (Tables 6–8). The ASDR and age-standardized DALYs rate for NPB attributable to short gestation and low birth weight were equivalent globally, across four continents, and in five different SDI countries (Tables 7–8), and were notably higher than those associated with particulate pollutants in the same contexts. Furthermore, in 2021, the ASDR and age-standardized DALYs rates for NDs, NE, NS, HD, and other neonatal disorders attributable to these three risk factors were consistently higher in males than in females, both globally and across four world regions and five different SDI countries. In 13 out of the 21 regions analyzed, the burden of NDs, NE, NS, HD, and other neonatal disorders due to these three risk factors was also found to be greater in males than in females (Tables 6–8).

## 9 Discussion

### 9.1 Global burden of neonatal disorders

This study indicates that, at the global level, from 1990 to 2021, the ASIR, ASDR, and age-standardized DALYs rate of NDs exhibited a declining trend, while the ASPR showed an increasing trend. Globally, the ASIR for NPB was the highest, consistent with previous studies, and the overall NPB ASIR demonstrated a declining trend (12). This phenomenon suggests that, at the global level, advancements in medical technology and increased professionalization of healthcare personnel have contributed to the decreasing trend in global NPB ASIR. Nevertheless, NPB remains a significant global issue and a leading cause of neonatal morbidity and mortality (24). In 2021, the global ASIR for HD was the lowest, with an EAPC of 0.02 (95% CI = −0.05 to 0.08) from 1990 to 2021. This finding suggests that health departments in various countries should prioritize the prevention of HD to further reduce its incidence and alleviate the burden of NDs. Furthermore, from 1990 to 2021, the global ASPR for NPB, NE, NS, and HD exhibited an upward trend, while the global rates of NDs, ASDR, and age-standardized DALYs for the five NDs etiologies demonstrated a downward trend. This indicates that, although the global prevalence of NDs has increased, the mortality and DALYs associated with NDs have decreased, attributed to improvements in healthcare systems and enhancements in healthcare standards across various countries.

### 9.2 Disease burden of neonatal disorders in different regions

Between 1990 and 2021, the EAPC indicated an upward trend in the ASPR of NDs across Africa, the Americas, Asia, and Europe. Conversely, the ASDR and the age-standardized DALYs rate for NDs in these regions demonstrated a downward trend. This indicates a growing prevalence of NDs, necessitating health policies that are tailored to the specific burdens of NDs in each country across these continents. For instance, in 2021, Africa exhibited the highest ASDR for NDs compared to the Americas, Asia, and Europe, which may be attributed to the continent’s slow economic development and limited healthcare resources. Prior studies have indicated that the obesity epidemic is increasingly affecting Africa, with maternal obesity linked to a heightened risk of neonatal mortality (25). In contrast, the overall burden of NDs in Europe was relatively lower compared to Africa, the Americas, and Asia in 2021, likely due to Europe’s proactive approach toward NDs. Historical data show that newborn screening programs were initiated in Europe during the 1960s, with a gradual expansion of screening scope since then (26). This initiative has laid a crucial foundation for alleviating the burden of NDs in Europe.

At the regional level, South Asia and Southern Sub-Saharan Africa bear a disproportionately high burden of NDs, influenced by various socio-economic factors. Previous studies have shown that these regions exhibit the highest rates of neonatal sepsis globally, primarily due to poverty, inadequate coverage of effective interventions, and disparities in healthcare delivery (27). In Southern Sub-Saharan Africa, bacterial sepsis is predominantly caused by Gram-negative pathogens, which exhibit significant antimicrobial resistance (28), resulting in high mortality rates associated with bacterial sepsis in the region. Furthermore, the incidence and mortality rates of neonatal surgical conditions in Southern Sub-Saharan Africa are alarmingly high, exacerbated by a limited number of pediatric specialists, insufficient specialization, and inadequate medical equipment (29). This indicates that health departments in Southern Sub-Saharan Africa should prioritize neonatal surgery and postoperative care for newborns, enhance the technical proficiency of medical personnel, and improve the standards of medical equipment. In 2021, the burden of NDs was notably lower in high-income Asia Pacific, Australia, and Eastern Europe, likely attributable to their more comprehensive healthcare systems, extended physician training periods, and higher levels of specialization. At the national level, from 1990 to 2021, over 50% of countries and regions showed a declining trend in the ASIR, ASDR, and age-standardized DALYs associated with NDs. Each country should formulate health policies tailored to the specific characteristics of the NDs burden to effectively alleviate this public health challenge.

According to the GBD 2021 database, the burden of NDs in low and low-middle SDI countries was significantly higher than that in high and high-middle SDI countries in 2021. Countries with low and low-middle SDI often exhibit relatively underdeveloped economies, lower healthcare levels, and inadequate standards of prevention and care. To alleviate the burden of NDs, it is essential for low and low-middle SDI countries to engage in collaborative exchanges with their high and high-middle SDI counterparts.

### 9.3 Gender differences in neonatal disorders burden

According to the GBD 2021 database, the global burden of NDs, NPB, NE, NS, HD, and other neonatal disorders in 2021 exhibited a higher prevalence in males than in females across four world regions, 21 regions, and five different SDI countries. Studies have indicated that the mortality rates of extremely low birth weight male and female infants with a gestational age of less than 30 weeks are similar (30); however, male infants tend to have a higher incidence of comorbidities. Conversely, other studies have shown that the gender difference in NDs is not significant in China (3). Further research is necessary to ascertain whether a gender difference exists in the burden of NDs. Additionally, the GBD 2021 database indicates that the burden of NDs attributable to risk factors in males was generally higher than that in females globally, across four world regions, 21 regions, and five countries with varying SDI levels. This underscores the need for countries worldwide to address the burden of NDs on males, particularly concerning particulate matter pollution, low birth weight, and short gestation, and to further investigate the underlying reasons for the higher burden of NDs in males.

### 9.4 Control of risk factors

From a risk factor perspective, low birth weight was identified as the primary contributor to the global ASDR and age-standardized DALYs rate for NDs in 2021. Furthermore, short gestation was found to be equivalent to low birth weight in its impact on the global ASDR and age-standardized DALYs rate for NPB. Studies conducted in 2022 indicated that postnatal growth continues to pose significant challenges for very low birth weight infants, particularly those affected by major NDs (31). Research from 2018 revealed that infants classified as small for gestational age have a higher likelihood of developing severe bronchopulmonary dysplasia compared to their appropriately sized counterparts (32). Notably, advancements in the quality of care have led to a significant reduction in both mortality and morbidity rates among preterm infants, especially those with very low birth weight (33). It is imperative for health departments worldwide to prioritize the prevention of NDs, enhance standards of care, and mitigate the burden of NDs associated with low birth weight risk factors.

### 9.5 Strategy suggestion

According to the GBD 2021 database, in 2021, the global ASDR attributable to NPB accounted for 40.4%, making NPB the leading cause of ASDR in 18 out of 21 regions. The risk factors for preterm birth are categorized into unmodifiable and modifiable factors (34). A study has provided evidence supporting the potential efficacy of stress reduction in lowering the incidence of NPB among low-risk women (35). This suggests that implementing stress reduction measures during pregnancy could decrease the incidence of NPB and alleviate the associated disease burden. The development of neonatal brain injury may be influenced by multiple factors, including the treatment of perinatal asphyxia (36). The development of neuroprotective drugs derived from natural plant products holds promise for the prevention and treatment of neonatal hypoxic–ischemic brain injury, warranting further research in this area. Regarding neonatal sepsis, studies have indicated that enhancing neonatal management is a crucial measure, which includes promoting breastfeeding and hygiene practices, among others (37, 38). Significant hyperbilirubinemia is more prevalent in infants born before full term and in neonates experiencing poor feeding and weight loss exceeding 10% (39). To mitigate the burden of hyperbilirubinemia, scientific feeding methods should be promoted to enhance neonatal weight.

### 9.6 Limitations of the study

This study has the following limitations. First, it relies on the GBD 2021 database, and the accuracy of its data may be affected by social, economic, nutritional, dietary, habit-related and other factors. Second, this study did not conduct descriptive analysis of age-related data and did not combine gender and age for further research. Third, this study only collated the global, five different SDI and gender-specific neonatal disorders ASDR and age-standardized DALYs rates caused by particulate matter pollution, short gestation, and low birth weight in 2021, and did not conduct in-depth research on other factors with relatively minor impacts, such as ambient particulate matter pollution and household air pollution from solid fuels.

## 10 Conclusion

NDs remain a significant global concern, with their impact shaped by a variety of factors. Between 1990 and 2021, the global burden of NDs exhibited an overall declining trend; however, regions such as South Asia and Southern Sub-Saharan Africa, as well as countries with low and low-middle SDI countries, continue to experience a heavier burden. Low birth weight is a critical contributor to the global burden of NDs, adversely affecting ASDR and age-standardized DALYs rates. To mitigate the burden of NDs, health departments in various countries should focus on improving healthcare systems, enhancing the quality of care, strengthening specialized training for healthcare professionals, cultivating high-quality medical talent, advancing medical technology, and implementing effective strategies to reduce the prevalence of NDs.

## Data Availability

The original contributions presented in the study are included in the article/supplementary material, further inquiries can be directed to the corresponding author.

## Footnotes

^1^ https://vizhub.healthdata.org/gbd-results/
