# Supplementary material for: Analysis of the global burden of neonatal disorders and risk factors from 1990 to 2021: findings from the global burden of disease study 2021
Source: Front Public Health. 2025 Oct 24;13:1618334. doi: 10.3389/fpubh.2025.1618334 (PMC12592090; doi:10.3389/fpubh.2025.1618334)
Supplement: Supplementary file 1 [file Data_Sheet_1.ZIP › Data/Figure 9/Other neonatal disorders/Deaths/CII2025-03-09_04-35-05.pdf]

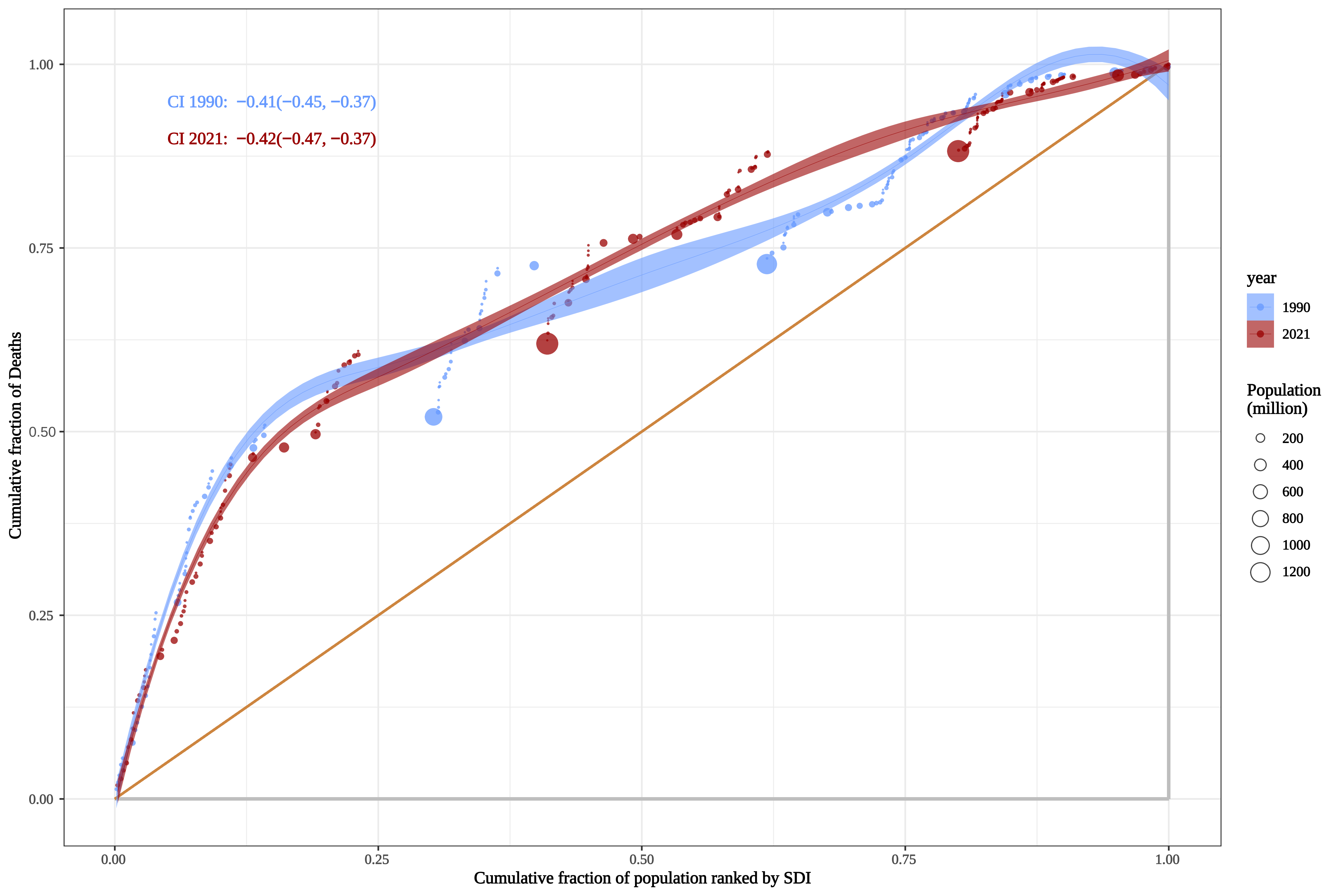

CI 1990: −0.41(−0.45, −0.37)
CI 2021: −0.42(−0.47, −0.37)
Cumulative fraction of Deaths
1.00
0.75
0.50
0.25
0.00
0.00
0.25
0.50
0.75
1.00
Cumulative fraction of population ranked by SDI
year
1990
2021
Population (million)
200
400
600
800
1000
1200
