# Supplementary material for: Analysis of the global burden of neonatal disorders and risk factors from 1990 to 2021: findings from the global burden of disease study 2021
Source: Front Public Health. 2025 Oct 24;13:1618334. doi: 10.3389/fpubh.2025.1618334 (PMC12592090; doi:10.3389/fpubh.2025.1618334)
Supplement: Supplementary file 1 [file Data_Sheet_1.ZIP › Data/Figure 9/Neonatal disorders/DALYs/CII2025-03-09_04-23-26.pdf]

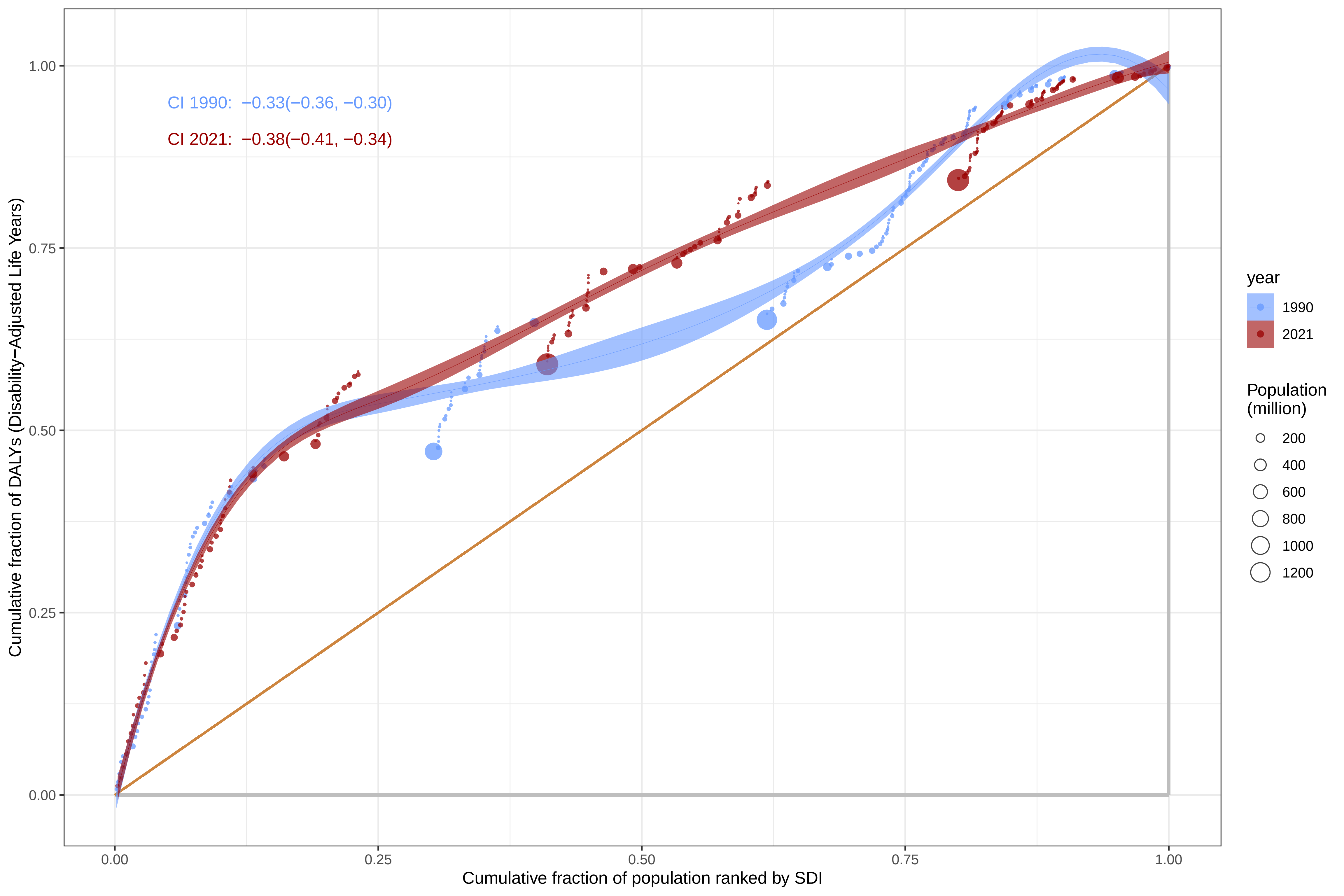

CI 1990: −0.33(−0.36, −0.30)
CI 2021: −0.38(−0.41, −0.34)
Cumulative fraction of DALYs (Disability−Adjusted Life Years)
Cumulative fraction of population ranked by SDI
0.00
0.25
0.50
0.75
1.00
year
1990
2021
Population (million)
200
400
600
800
1000
1200
