# Supplementary material for: Analysis of the global burden of neonatal disorders and risk factors from 1990 to 2021: findings from the global burden of disease study 2021
Source: Front Public Health. 2025 Oct 24;13:1618334. doi: 10.3389/fpubh.2025.1618334 (PMC12592090; doi:10.3389/fpubh.2025.1618334)
Supplement: Supplementary file 1 [file Data_Sheet_1.ZIP › Data/Figure 9/Neonatal encephalopathy due to birth asphyxia and trauma/DALYs/CII2025-03-09_04-29-09.pdf]

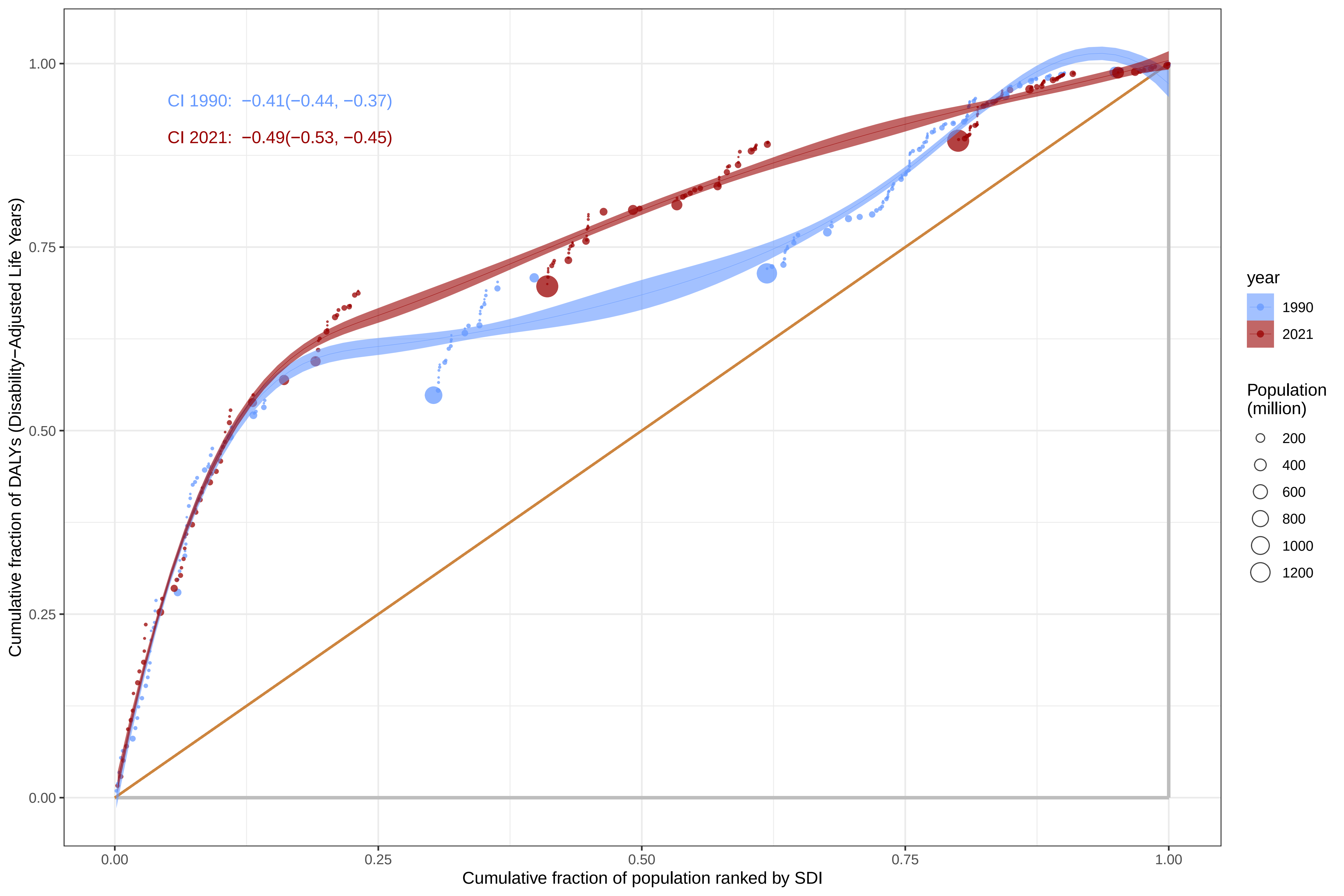

CI 1990: −0.41(−0.44, −0.37)
CI 2021: −0.49(−0.53, −0.45)
Cumulative fraction of DALYs (Disability−Adjusted Life Years)
Cumulative fraction of population ranked by SDI
0.00
0.25
0.50
0.75
1.00
year
1990
2021
Population (million)
200
400
600
800
1000
1200
