# Supplementary material for: Analysis of the global burden of neonatal disorders and risk factors from 1990 to 2021: findings from the global burden of disease study 2021
Source: Front Public Health. 2025 Oct 24;13:1618334. doi: 10.3389/fpubh.2025.1618334 (PMC12592090; doi:10.3389/fpubh.2025.1618334)
Supplement: Supplementary file 1 [file Data_Sheet_1.ZIP › Data/Figure 11/Neonatal disorders/Decomposition analysis 2025-03-16_22-11-42.pdf]

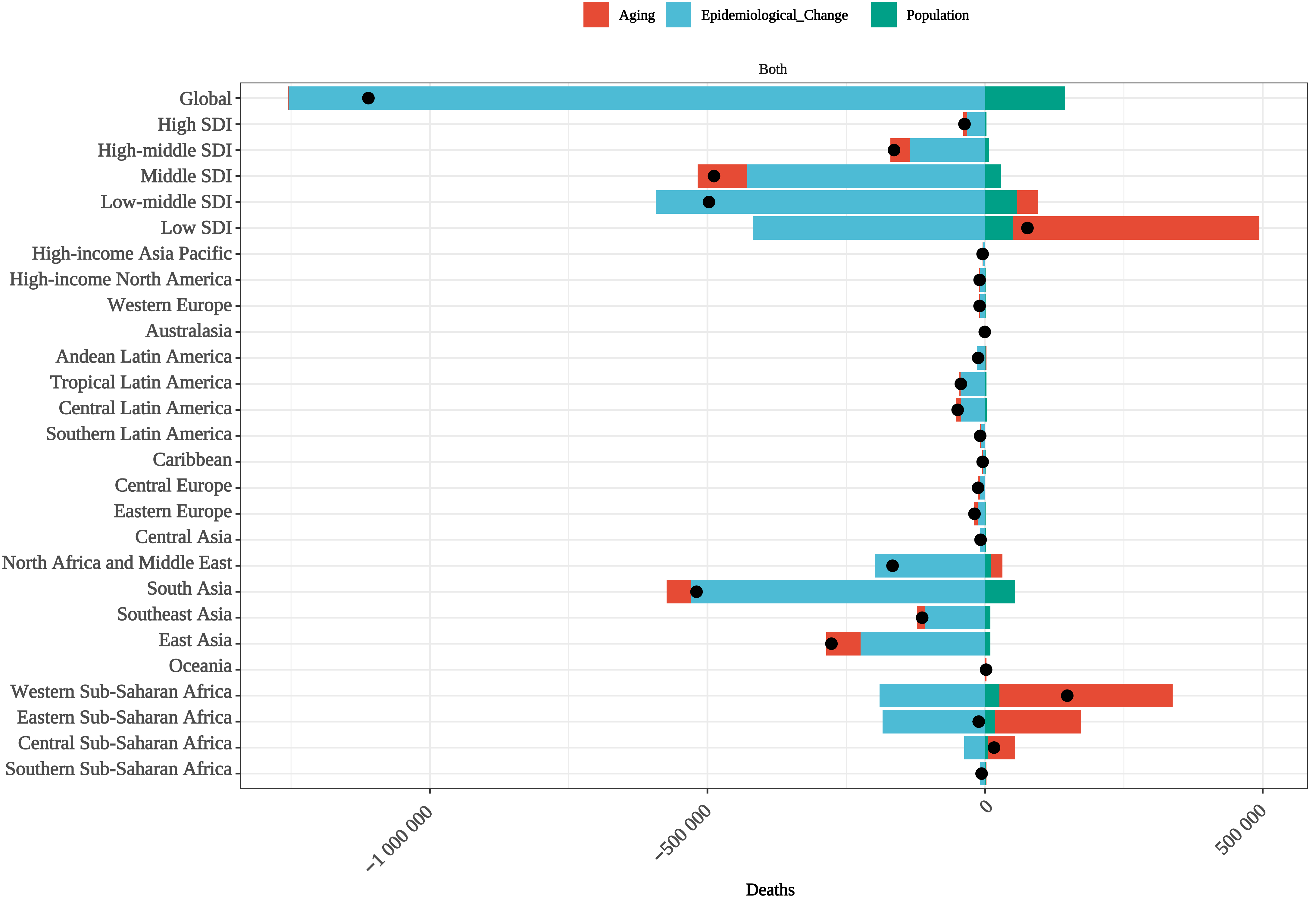

Aging
Epidemiological_Change
Population
Both
Global
High SDI
High-middle SDI
Middle SDI
Low-middle SDI
Low SDI
High-income Asia Pacific
High-income North America
Western Europe
Australasia
Andean Latin America
Tropical Latin America
Central Latin America
Southern Latin America
Caribbean
Central Europe
Eastern Europe
Central Asia
North Africa and Middle East
South Asia
Southeast Asia
East Asia
Oceania
Western Sub-Saharan Africa
Eastern Sub-Saharan Africa
Central Sub-Saharan Africa
Southern Sub-Saharan Africa
−1 000 000
−500 000
0
500 000
Deaths
