# Supplementary material for: Analysis of the global burden of neonatal disorders and risk factors from 1990 to 2021: findings from the global burden of disease study 2021
Source: Front Public Health. 2025 Oct 24;13:1618334. doi: 10.3389/fpubh.2025.1618334 (PMC12592090; doi:10.3389/fpubh.2025.1618334)
Supplement: Supplementary file 1 [file Data_Sheet_1.ZIP › Data/Figure 10/Neonatal encephalopathy due to birth asphyxia and trauma/Deaths/SII2025-03-09_15-13-02.pdf]

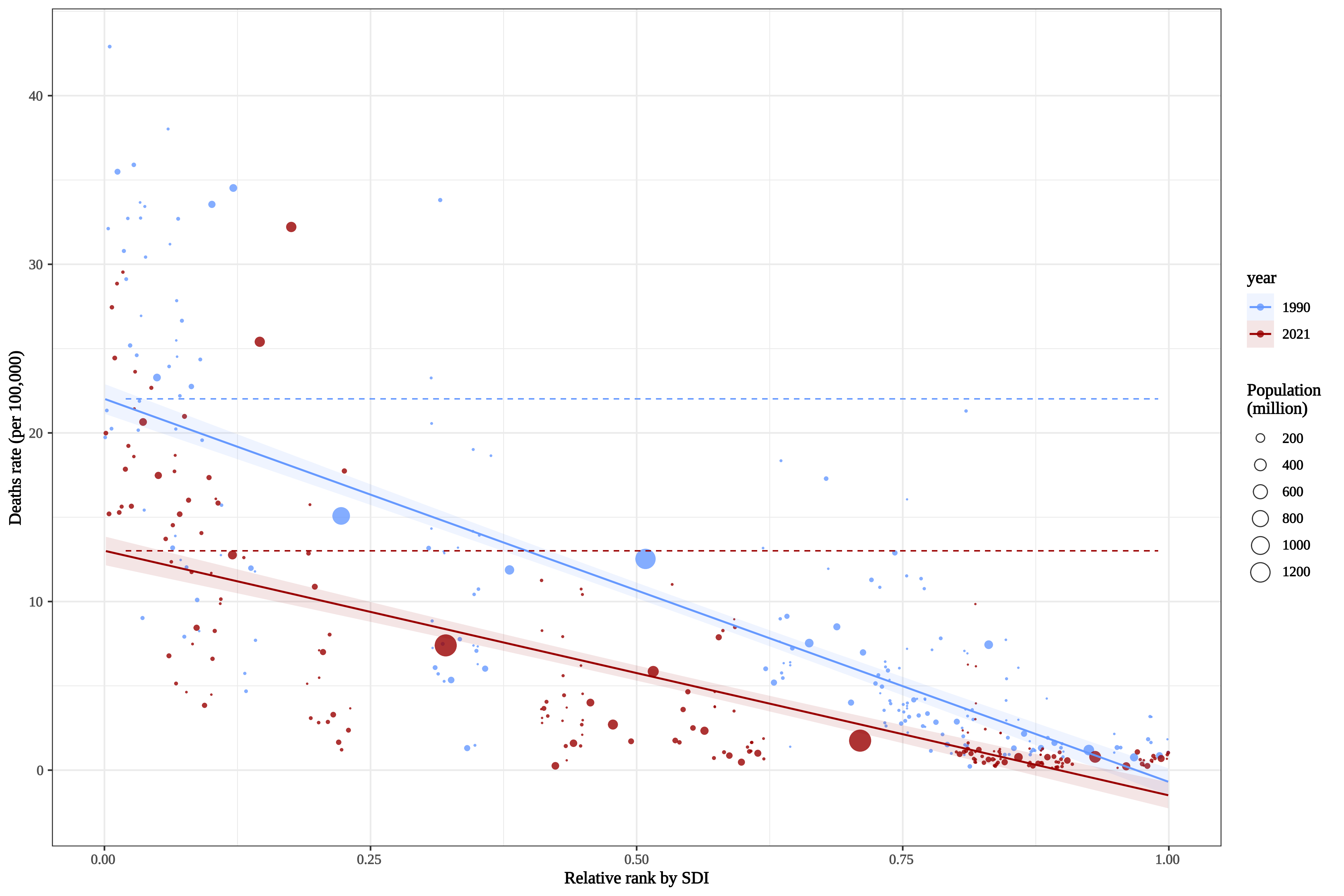

Deaths rate (per 100,000)
0
10
20
30
40
0.00
0.25
0.50
0.75
1.00
Relative rank by SDI
year
1990
2021
Population
(million)
200
400
600
800
1000
1200
